# Supplementary material for: Spleen-targeted neoantigen mRNA vaccine induces ISG15+ CD8+ T cell-mediated tertiary lymphoid structure formation in hepatocellular carcinoma
Source: Cell Rep Med. 2026 Apr 20;7(5):102754. doi: 10.1016/j.xcrm.2026.102754 (PMC13198314; doi:10.1016/j.xcrm.2026.102754)
Supplement: Document S1. Figures S1–S25 and Table S1 [file mmc1.pdf]

Cell Reports Medicine, Volume *7*

Supplemental information

Spleen-targeted neoantigen mRNA vaccine induces ISG15^{+} CD8^{+} T cell-mediated tertiary lymphoid structure formation in hepatocellular carcinoma

Xinyi Lin, Geng Chen, Ruijing Tang, Ming Wu, Da Zhang, Fangzhou Lin, Jianhua Guan, Jing Yang, Xiuqing Dong, Xiaoyuan Zheng, Liman Qiu, Haijun Yu, Zhixiong Cai, and Xiaolong Liu

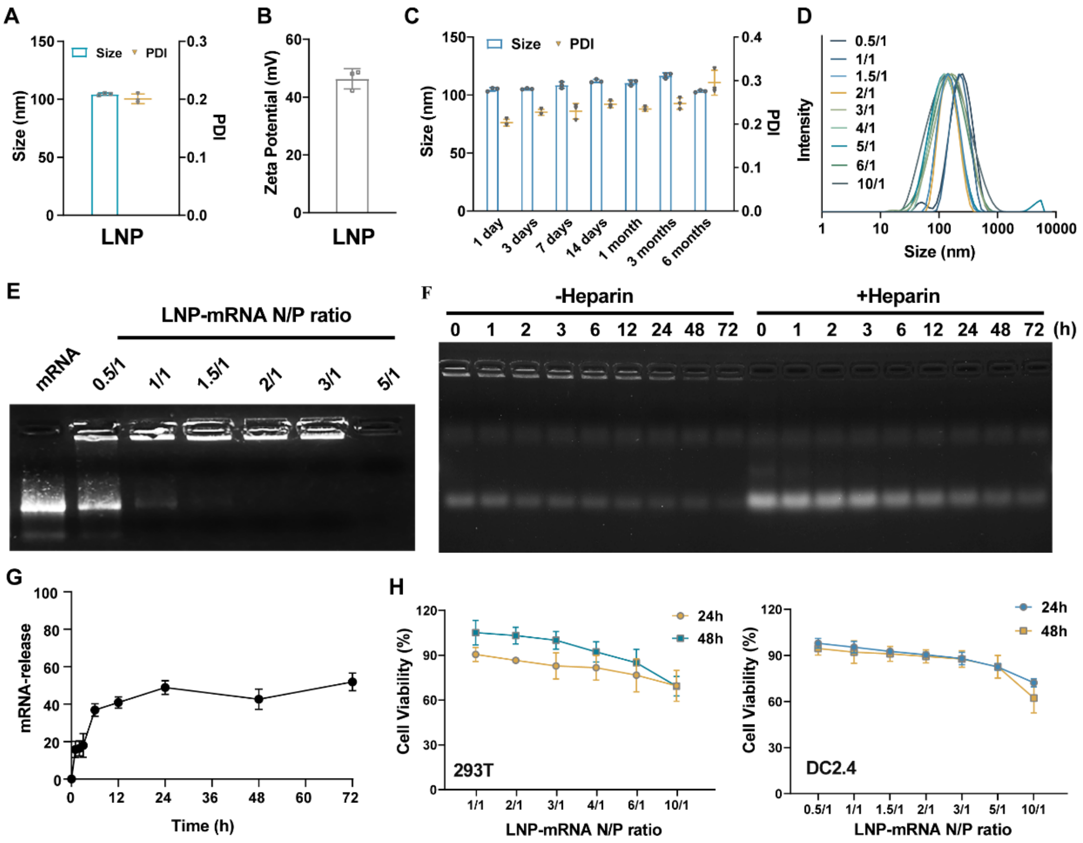

Figure S1. Synthesis and characterization of LNP-mRNA. Related to Figure 1.

(A) Particle size (104.4 nm) and polydispersity index (PDI = 0.201) of DOTMA/DOPE LNPs (n = 3 independent preparations).

(B) Zeta potential of pure LNPs (46.3 mV), indicating strong electrostatic capacity for complexing negatively charged mRNA (n = 3 independent preparations).

(C) Particle size and PDI of LNPs during storage at 4 °C for 6 months, showing minimal change and confirming colloidal stability (n = 3 independent preparations).

(D) Representative particle size distribution of LNP-mRNA complexes at different N/P ratios.

(E) Agarose gel electrophoresis of LNP-mRNA at different N/P ratios, with naked mRNA as a migration reference. Complete mRNA retention was observed at N/P ≥ 1.5.

(F) Agarose gel analysis of LNP-mRNA (N/P = 0.5) incubated in 10% serum-containing medium supplemented with RNase R (final 3 U/mL) at 37 °C with orbital shaking (200 rpm) for 0-72 h. Untreated lanes (left) show a gradual loss of both retained and migrating mRNA signals, whereas heparin-treated lanes (final 10 mg/mL) reflect the total recoverable mRNA (right). A molecular size marker was not included because the assay evaluates relative mRNA release and protection. Representative result from 3 independent biological replicates.

(G) Quantitative analysis of mRNA release measured by RiboGreen fluorescence after heparin displacement (final 10 mg/mL). An initial burst release (~35%) occurred within 6 h, reaching a plateau (~50%) by 24 h, indicating that a substantial fraction of mRNA remains LNP-associated despite serum nuclease exposure. Data are presented as mean ± SD (n = 3 biological replicates).

(H) Cytotoxicity of LNP-mRNA in 293T and DC2.4 cells assessed by CCK-8. Data are presented as mean ± SD (n = 5 biological replicates). LNP-mRNA showed good biocompatibility across N/P ratios tested, except at N/P = 10, where a moderate reduction in cell viability was observed.

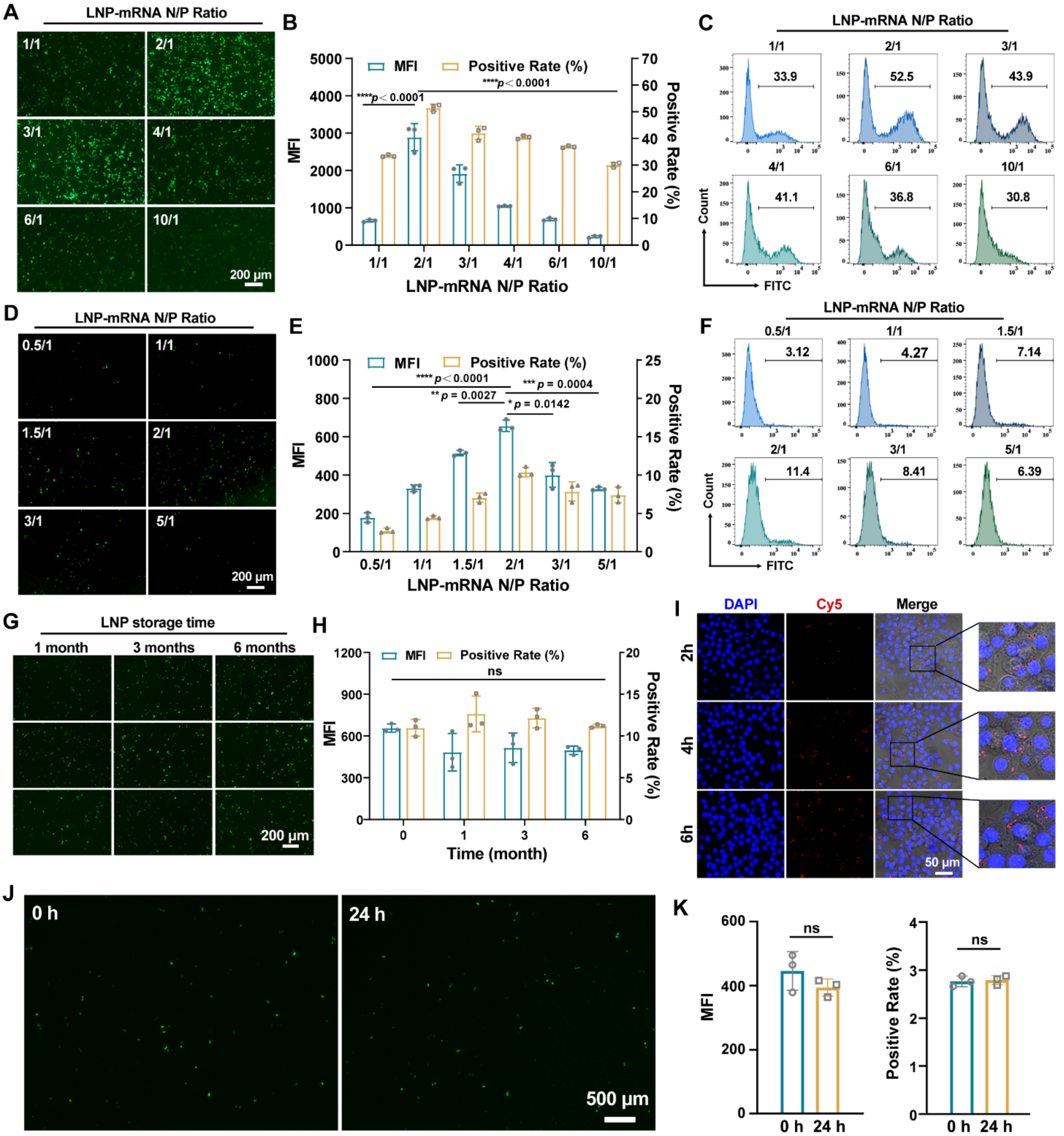

A
LNP-mRNA N/P Ratio
1/1
2/1
3/1
4/1
6/1
10/1
200 μm
B
MFI
Positive Rate (%)
****p < 0.0001
****p < 0.0001
LNP-mRNA N/P Ratio
C
LNP-mRNA N/P Ratio
33.9
52.5
43.9
41.1
36.8
30.8
Count
FITC
D
LNP-mRNA N/P Ratio
0.5/1
1.5/1
3/1
5/1
E
**** p < 0.0001
*** p = 0.0004
** p = 0.0027
* p = 0.0142
F
3.12
4.27
7.14
11.4
8.41
6.39
G
LNP storage time
1 month
3 months
6 months
H
ns
Time (month)
I
DAPI
Cy5
Merge
2h
4h
6h
50 μm
J
0 h
24 h
500 μm
K

Figure S2. In vitro performance of LNP-mRNA. Related to Figure 1.

For all in vitro experiments, mRNA encoding GFP ($mRNA^{GFP}$) was used as the reporter, and cells were analyzed 24 h post-transfection unless otherwise noted.

(A-C) Transfection of 293T cells with LNP-$mRNA^{GFP}$ at indicated N/P ratios. (A) Representative fluorescence microscopy images. Scale bar, 200 μm. (B) Flow cytometry quantification of mean fluorescence intensity (MFI) and GFP-positive cell percentage, showing peak efficiency at N/P = 2. (C) Representative FITC histograms.

(D-F) Transfection of DC2.4 cells with LNP-$mRNA^{GFP}$ at indicated N/P ratios. (D) Representative fluorescence microscopy images. Scale bar, 200 μm. (E) Flow cytometry quantification of MFI and GFP-positive cell percentage, showing peak efficiency at N/P = 2. (F) Representative FITC histograms.

(G-H) Transfection efficiency of LNP-$mRNA^{GFP}$ (N/P = 2) in DC2.4 cells using LNPs stored at 4 °C for 1-6 months. (G) Representative fluorescence microscopy images. Scale bar, 200 μm. (H) Flow cytometry quantification showing MFI and GFP-positive cell percentage, indicating that long-term storage does not impair delivery efficiency.

(I) Confocal laser scanning microscopy (CLSM) images showing intracellular uptake of LNP-$mRNA^{Cy5}$ (N/P = 2) in DC2.4 cells after 2-6 h of incubation. Scale bar, 50 μm.

(J-K) Transfection of DC2.4 cells with freshly prepared or serum-exposed LNP-$mRNA^{GFP}$ (10% serum, 4 °C, 24 h; N/P = 0.5). (J) Representative fluorescence microscopy images. Scale bar, 500 μm. (K) Flow cytometry quantification showing MFI and GFP-positive cell percentage 24 h post-transfection. Brief serum exposure did not significantly affect transfection efficiency, indicating that DOTMA/DOPE LNPs retain structural integrity and delivery function under short-term handling conditions.

Statistics: Data are presented as mean ± SD (n = 3 biological replicates for all quantifications). For N/P ratio comparisons (B, E), one-way ANOVA was performed (comparing N/P = 2 vs. other ratios). For LNP storage comparisons (H), one-way ANOVA was used to assess differences among time points (1-6 months). For serum-exposure comparisons (K), a two-tailed paired t-test was applied (fresh vs. 24 h serum-exposed within each replicate). Significance levels: *$p < 0.05$; **$p < 0.01$; ***$p < 0.001$; ****$p < 0.0001$.

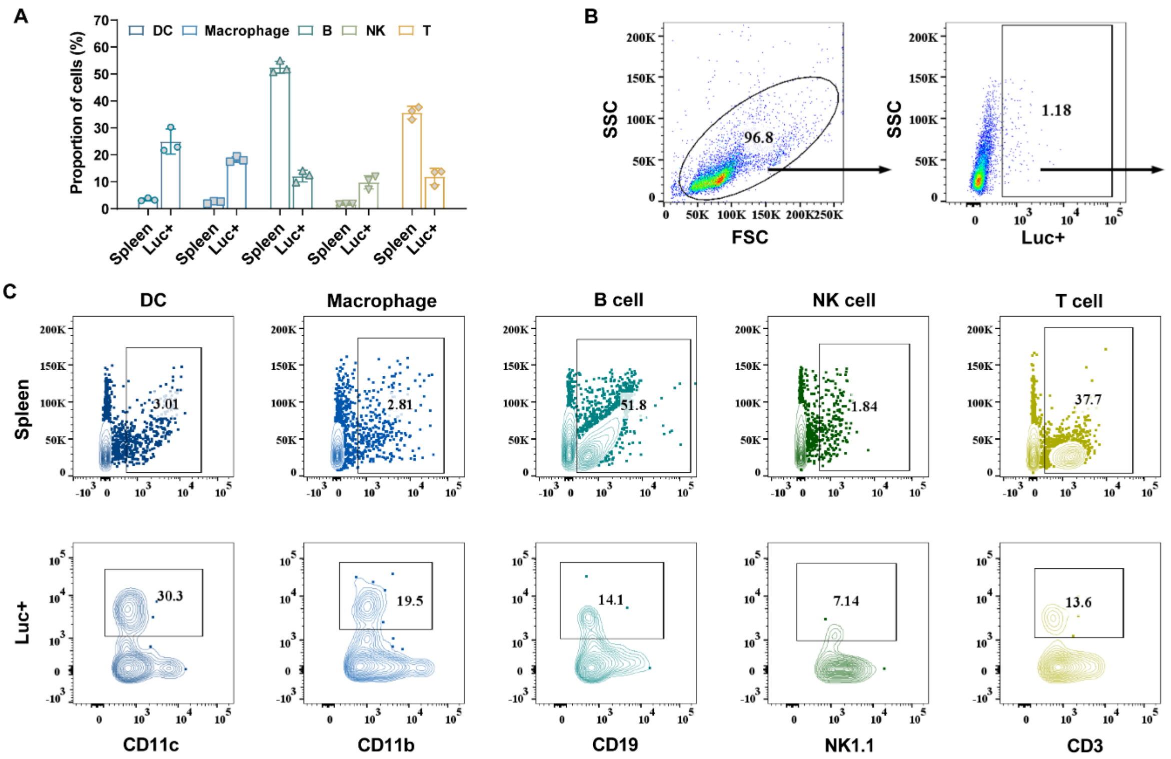

Figure S3. Cellular composition of the spleen and distribution of luciferase expression among immune subsets after LNP-mRNA administration. Related to Figure 2.

Firefly luciferase mRNA (mRNA^{Fluc}) was used as the reporter. Splenic single-cell suspensions were prepared by mechanical dissociation followed by red blood cell lysis and analyzed 6 h post-administration.

(A) Flow cytometric quantification of major splenic immune cell populations (DCs, macrophages, B cells, NK cells, and T cells) and their respective proportions within luciferase-positive (Luc+) cells. Despite their low abundance in the spleen (DCs 3.42%, macrophages 2.46%), DCs accounted for the highest proportion of Luc+ cells (24.97%), followed by macrophages (18.33%), B cells (12.15%), T cells (11.94%), and NK cells (9.85%), indicating preferential transfection of professional antigen-presenting cells by DOTMA/DOPE LNPs. Data are presented as mean ± SD (n = 3 biological replicates).

(B) Representative gating strategy for identifying Luc+ cell subsets from splenic single-cell suspensions.

(C) Representative density plots showing the indicated immune subsets in total splenic versus Luc+ populations. Luciferase expression was detected by intracellular staining with PE-conjugated anti-firefly luciferase antibody after cell fixation and permeabilization.

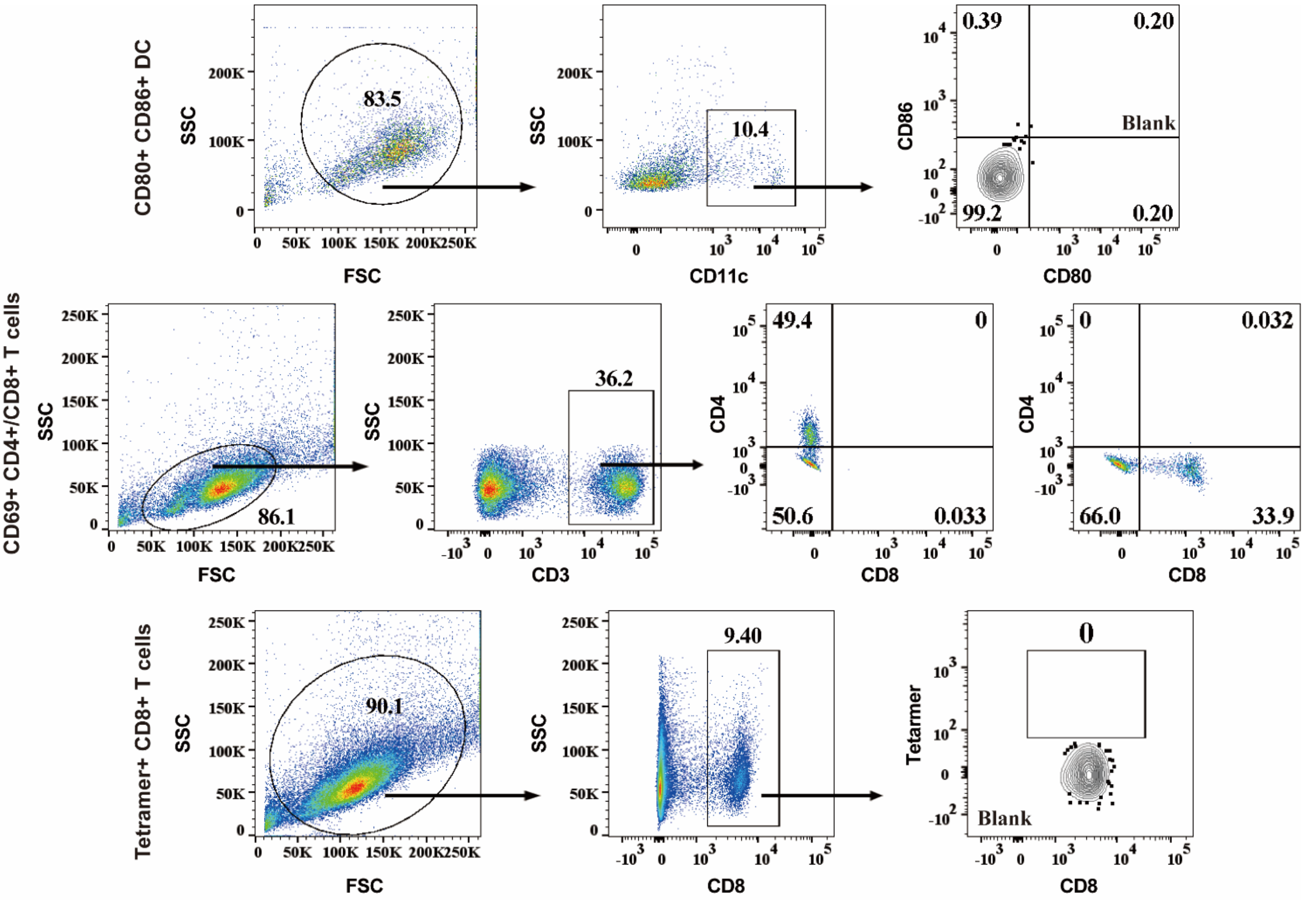

Figure S4. Flow cytometry gating strategy for assessing splenic immune cell populations. Related to Figure 2E. Representative gating sequence used to identify and quantify splenic CD80+ CD86+ DCs, CD69+ CD4+ T cells, CD69+ CD8+ T cells, and Ptpn2-specific CD8+ T cells after STNvac administration.

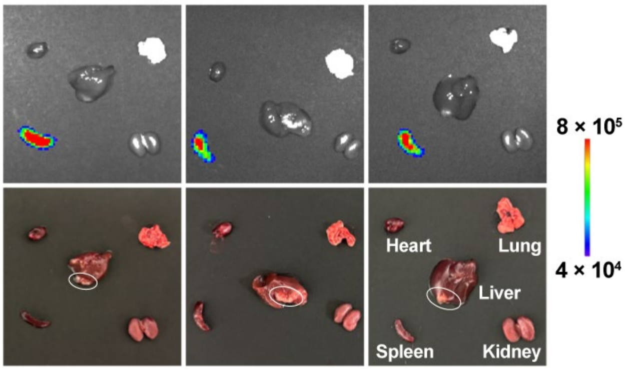

Figure S5. In vivo biodistribution of mRNA expression in orthotopic HCC-bearing mice. Related to Figure 3. Ex vivo bioluminescence imaging (top) and corresponding bright-field photographs (bottom) of major organs (heart, liver (HCC-bearing), spleen, lung, and kidney) collected 6 h after intravenous administration of LNP-mRNA^{Fluc} (N/P = 0.5; 10 µg mRNA per mouse) in orthotopic HCC-bearing C57BL/6 mice one week after tumor implantation. The tumor sites on the liver were outlined with white circles in the bright-field images. Predominant luciferase expression was observed in the spleen.

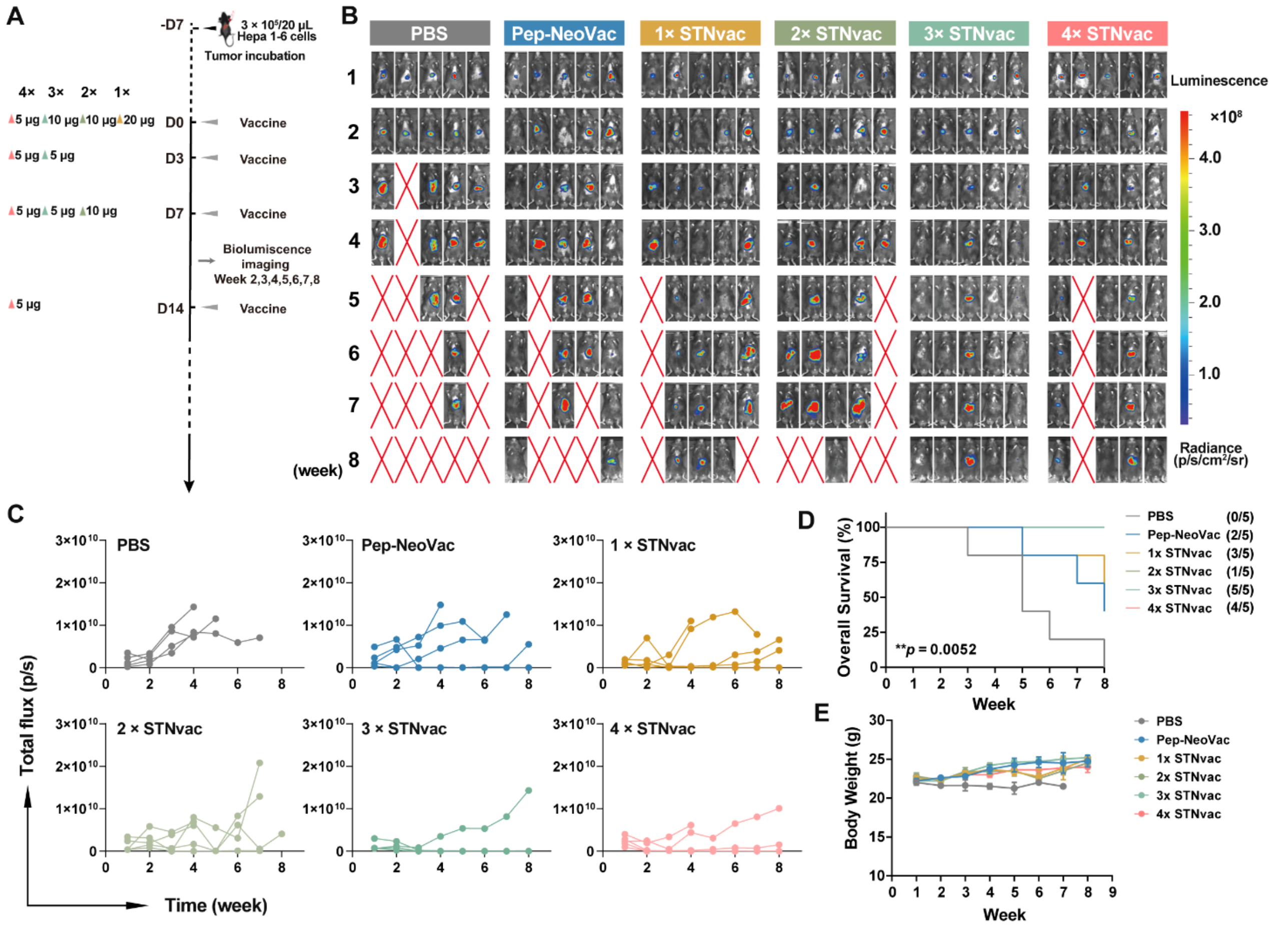

Figure S6. Optimization of STNvac vaccination regimen. Related to Figure 3.

(A) Schematic illustration of the vaccination protocol comparing single (1×), double (2×), triple (3×), and quadruple (4×) administrations with a fixed total mRNA dose of 20 μg per mouse (n = 5 mice per group). The peptide-based vaccine (Pep-NeoVac, formulated with Poly I:C) was administered subcutaneously in a three-dose regimen (2 μg per peptide, total 14 μg) at comparable intervals (day 0, 4, and 8), following the protocol established in our previous study (J Immunother Cancer, 2022, 10, e004389).

(B) Bioluminescence images showing tumor burden in orthotopic HCC-bearing mice during an 8-week observation period. Tumor growth in the PBS group was rapid, whereas varying degrees of regression were observed in all STNvac-treated groups. The 3× group showed the highest progression-free survival (PFS) rate of 80%, while the PFS rates for the PBS, 1×, 2×, and 4× groups were 0%, 20%, 20%, and 40%, respectively. Across all regimens, STNvac consistently outperformed the peptide-based vaccine, exhibiting superior tumor control. The enhanced efficacy of the three-dose regimen is attributable to a homologous prime-boost effect, in which the initial dose primes the immune system and subsequent doses reinforce and prolong the response. In this study, the three-dose schedule also surpassed the four-dose regimen, possibly because of its stronger priming and earlier intervention during initial tumor growth.

(C) Total bioluminescence flux for individual mice corresponding to (B).

(D) Survival curves of mice receiving different vaccination regimens. By the end of the monitoring period, no PBS-treated mice survived (0/5), whereas the 3× group maintained complete survival. Statistics: Log-rank test for trend; $^{**}p < 0.01$.

(E) Mean body weight of mice monitored throughout the study period, showing no significant weight loss, confirming the biosafety of STNvac.

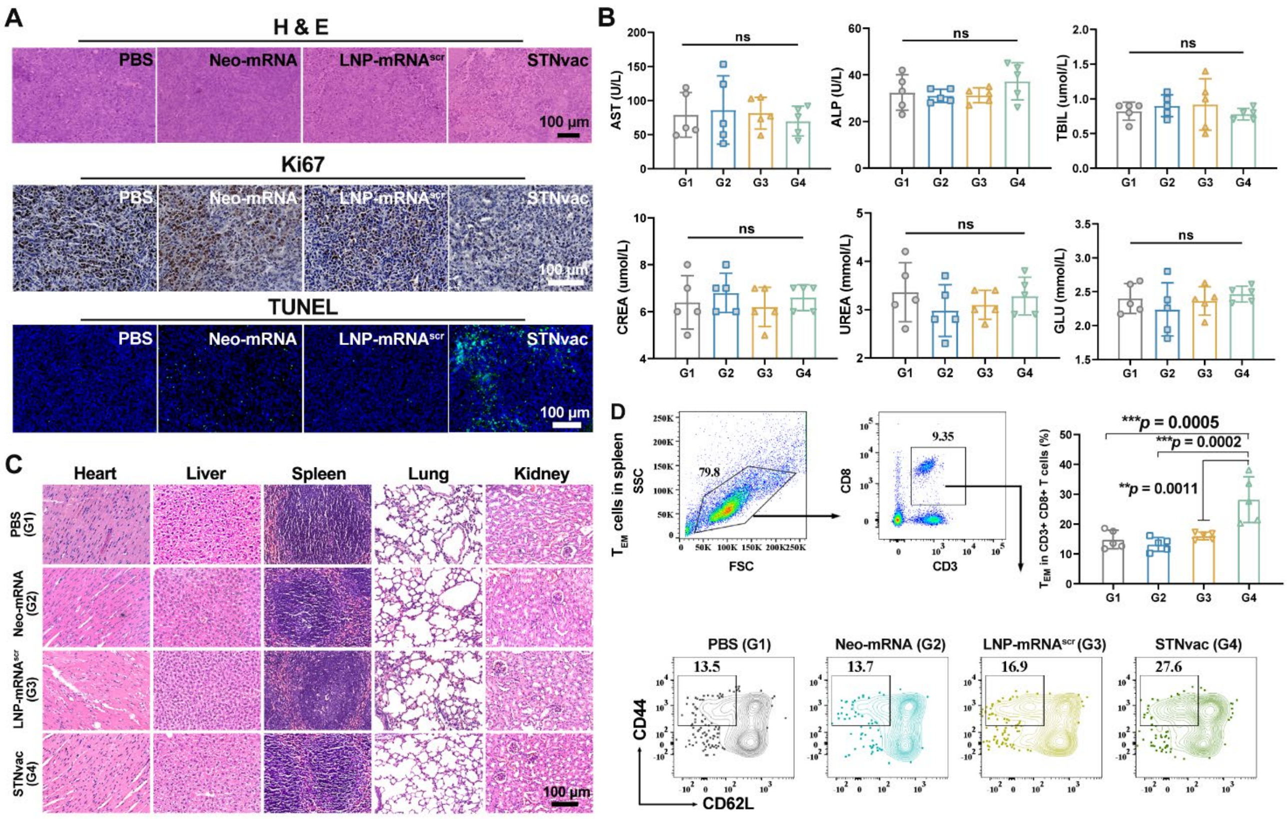

Figure S7. Antitumor effects and acute tolerability of STNvac evaluated in orthotopic HCC mouse model. Related to Figure 3.

(A) H&E, Ki67, and TUNEL staining of dissected tumor sections collected 3 days after the final vaccination (day 10), showing tissue morphology, proliferation, and apoptosis. Scale bars, 100 μm.

(B-C) Acute tolerability assessment of STNvac: (B) Serum biochemical analysis and (C) H&E staining of major organs harvested on day 10. Scale bar, 100 μm.

(D) Flow cytometry analysis of effector memory CD8+ T cells (CD8+ T_{EM}) in spleens collected on day 10. STNvac treatment significantly increased the proportion of CD8+ T_{EM} (28.2% of CD3+ CD8+ T cells) compared with PBS-treated mice (14.8%), indicating enhanced immune protection induced by STNvac.

Statistics: One-way ANOVA for (B) and (D); Data are presented as mean ± SD (n = 5 biological replicates). Significance levels: $**p < 0.01$; $***p < 0.001$.

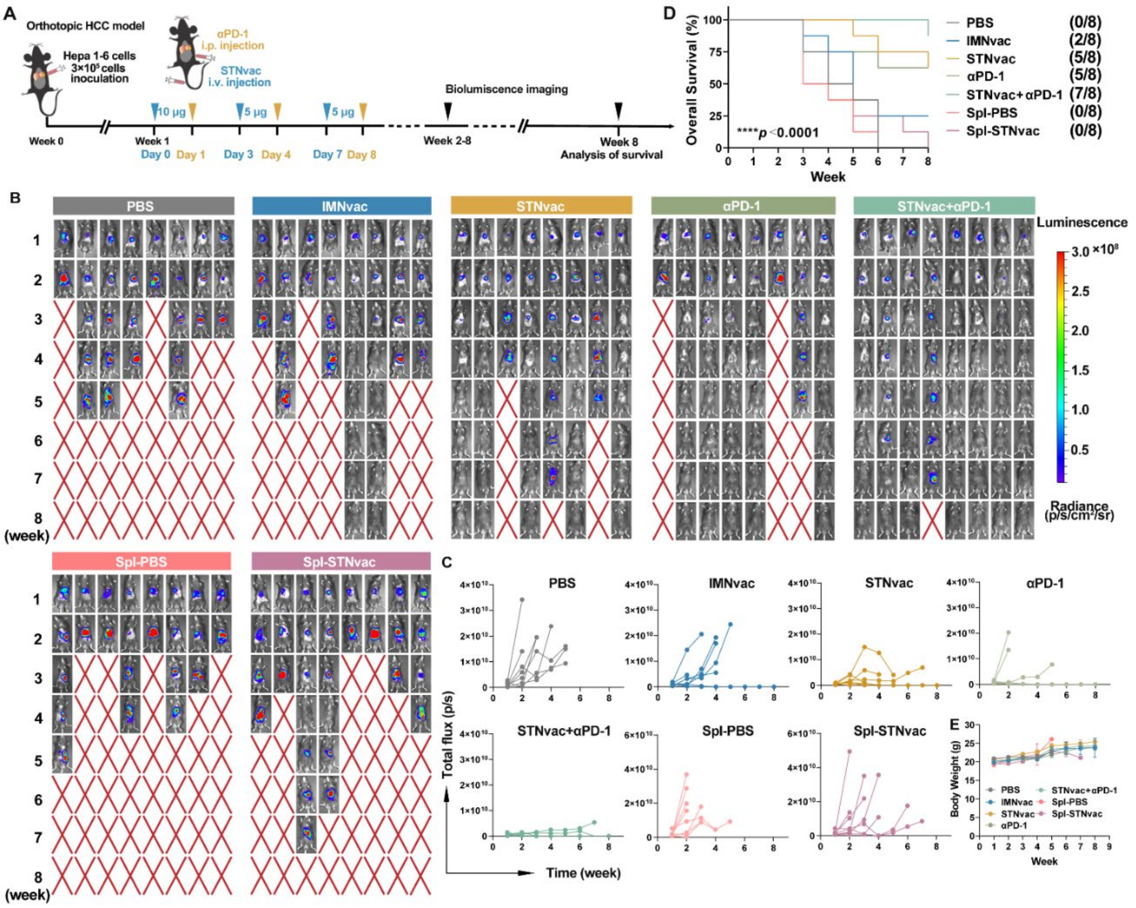

Figure S8. Therapeutic evaluation of STNvac across administration routes, with checkpoint blockade, and in splenectomized mice. Related to Figure 3. (A) Schematic illustration of the vaccination and treatment protocol including seven groups: PBS, intramuscular IMNvac (formulated with the FDA-approved SM-102-based LNP system used in Moderna's mRNA-1273 vaccine), intravenous STNvac, αPD-1 antibody, STNvac + αPD-1 combination, and splenectomized mouse models (Spl-PBS and Spl-STNvac). All mRNA vaccines were administered in a three-dose regimen (days 0, 3, 7; total 20 μg mRNA per mouse; n = 8 mice per group). (B) Bioluminescence images showing tumor burden over the 8-week observation period. Intravenous STNvac induced markedly stronger tumor suppression than intramuscular IMNvac, highlighting the advantage of spleen-targeted delivery. Rapid tumor regression appeared as early as week 2 in the STNvac+αPD-1 group, whereas STNvac monotherapy showed evident responses around weeks 3-4. Responses to αPD-1 monotherapy were heterogeneous, with responding mice showing rapid tumor regression, whereas non-responders exhibited outcomes comparable to PBS-treated controls and experienced early mortality, indicating variable therapeutic sensitivity to PD-1 blockade. In contrast, the STNvac+αPD-1 combination resulted in more consistent tumor control across mice, reflecting an increased response rate compared with αPD-1 monotherapy. In splenectomized cohorts, tumor progression was accelerated and the therapeutic benefit of STNvac observed in intact-spleen mice was largely lost, confirming spleen-dependent efficacy. (C) Total bioluminescence flux for individual mice corresponding to (B). (D) Survival curves of mice in different treatment groups. The STNvac+αPD-1 combination showed the highest overall survival (7/8), followed by STNvac and αPD-1 monotherapy (5/8). In contrast, IMNvac resulted in limited survival benefit (2/8), and all PBS-treated mice succumbed during the observation period. Splenectomy markedly diminished the benefit of STNvac, with Spl-PBS and Spl-STNvac groups succumbing by week 8. (E) Mean body weight of mice monitored throughout the study period, showing no significant loss. Statistics: Log-rank (Mantel-Cox) test for survival analysis (D). Significance levels: ****$p < 0.0001$.

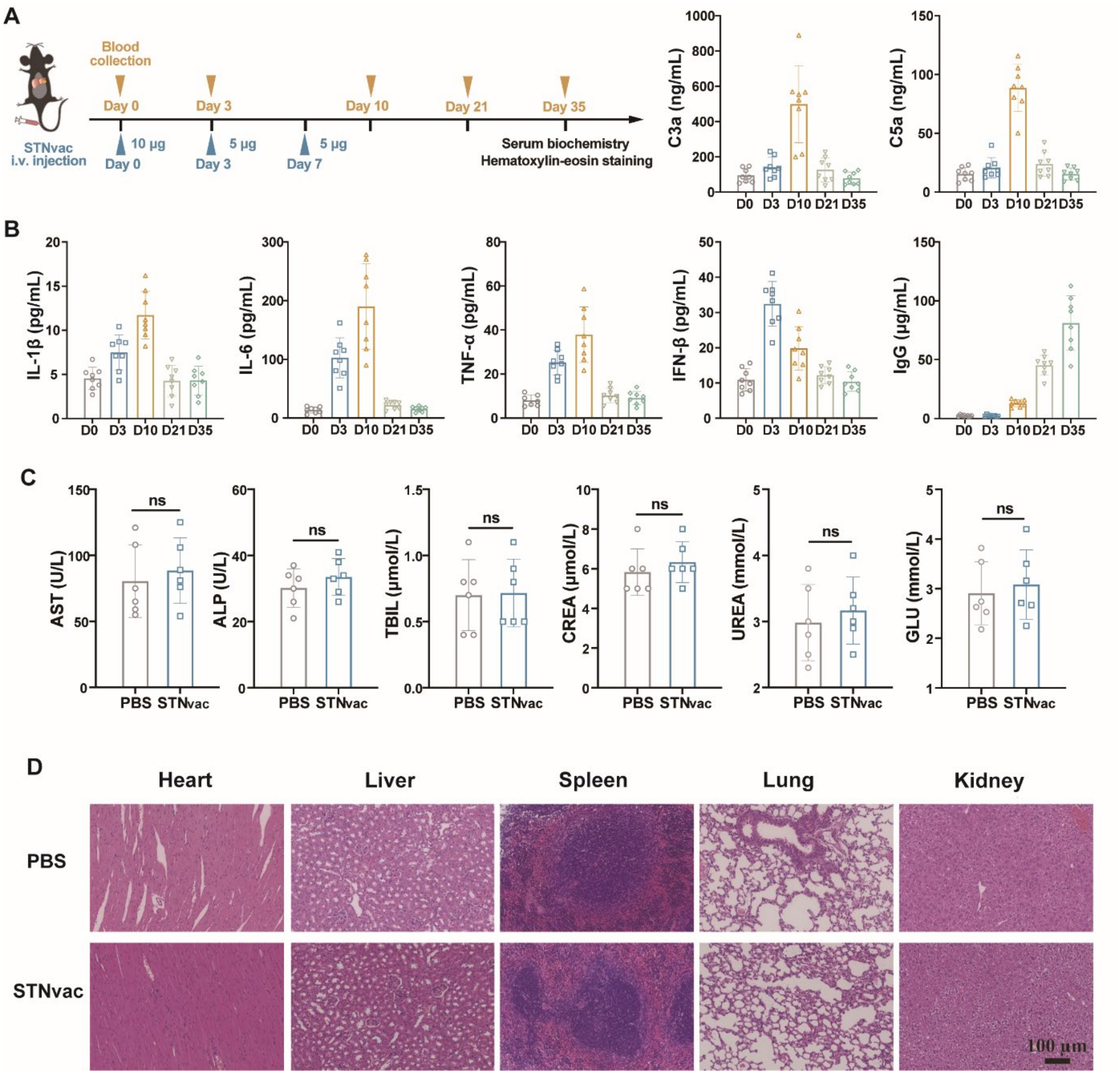

Figure S9. Systemic safety evaluation of STNvac in tumor-free mice. Related to Figure 3.

(A) Schematic illustration of the biosafety assessment protocol. Tumor-free C57BL/6 mice received three intravenous injections of STNvac on days 0, 3, and 7. Blood was collected via orbital capillary sampling at designated time points (days 0, 3, 10, 21 and 35) for cytokine and complement analysis, and at day 35 for serum biochemistry. Major organs were harvested on day 35 for H&E staining.

(B) Serum cytokine (IL-1β, IL-6, TNF-α, IFN-β) and complement (C3a, C5a) levels at different time points, together with total IgG levels. Each bar represents pooled serum obtained from parallel groups of mice (n = 8 per time point). IFN-β peaked on day 3, whereas IL-1β, IL-6, TNF-α, C3a, and C5a peaked on day 10, followed by a progressive decline to baseline by day 21 and stabilization through day 35, indicating transient and self-limited innate activation without sustained systemic inflammation. Total IgG increased beginning on day 10 and continued to rise, reaching a maximum by day 35, consistent with the induction of a sustained humoral response.

(C) Serum biochemistry of liver and kidney function at Day 35. Data represent individual mice (n = 6 biological replicates, mean ± SD). No significant differences were detected between STNvac-treated and control mice.

(D) Representative H&E staining of major organs (heart, liver, spleen, lung, kidney) from PBS control and STNvac-treated mice at day 35, showing no histopathological abnormalities. Scale bar, 100 μm.

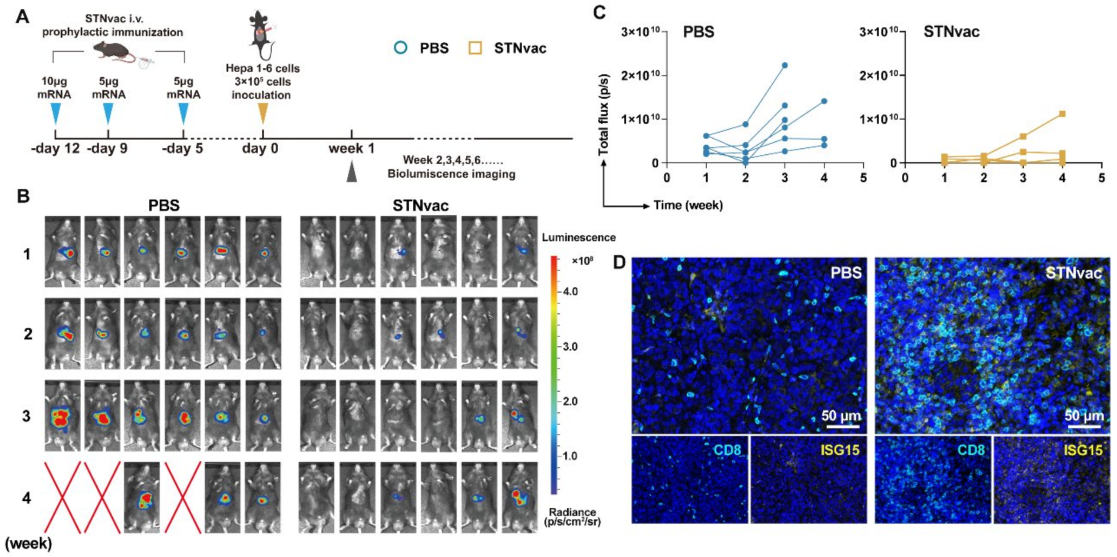

Figure S10. Prophylactic efficacy of STNvac in preventing HCC initiation. Related to Figure 3.

(A) Schematic illustration of the prophylactic vaccination and tumor challenge protocol. Healthy C57BL/6 mice were immunized with STNvac, followed by intrahepatic injection of Hepa1-6-Luc cells 5 days after the final vaccination (n = 6 mice per group).

(B) Bioluminescence imaging showing tumor burden during the 4-week observation period. Rapid tumor progression was observed in the PBS group, whereas STNvac-immunized mice exhibited markedly reduced tumor formation and overall burden, with half showing complete tumor rejection.

(C) Total bioluminescence flux for individual mice corresponding to (B).

(D) Representative multicolor immunofluorescence images of ISG15 and CD8 co-staining in residual lesions from the prophylactic model, confirming the presence of ISG15+ CD8+ T cells in STNvac-immunized mice. Scale bars, 50 μm.

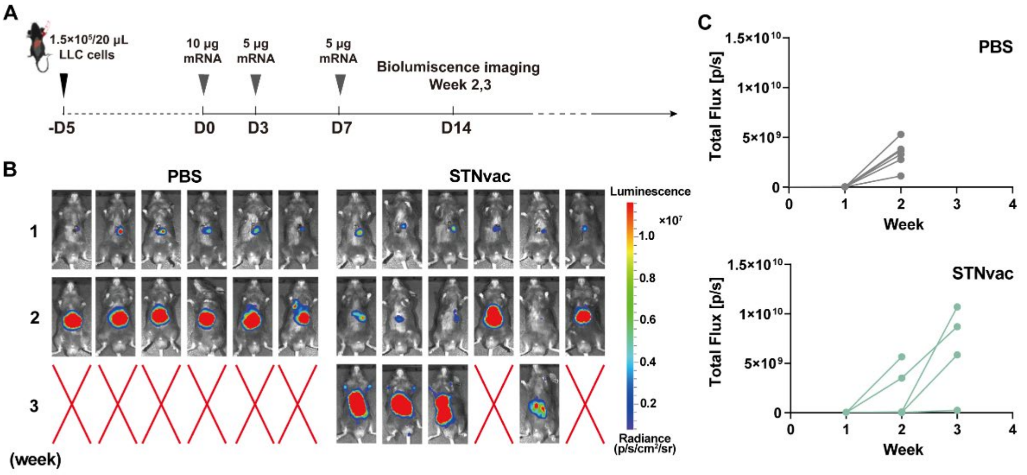

Figure S11. Therapeutic effects of STNvac in the LLC liver metastasis model. Related to Figure 3.

(A) Schematic illustration of the treatment schedule. Lewis lung carcinoma (LLC)-Luc cells were implanted into the livers of C57BL/6 mice to establish a liver metastasis model (n = 6 mice per group).

(B) Bioluminescence imaging showing tumor burden during the 3-week observation period. Rapid tumor progression was observed in the PBS group, whereas STNvac-treated mice showed delayed tumor growth and partially reduced tumor burden, though complete regression was not achieved due to the high malignancy of LLC.

(C) Total bioluminescence flux for individual mice corresponding to (B).

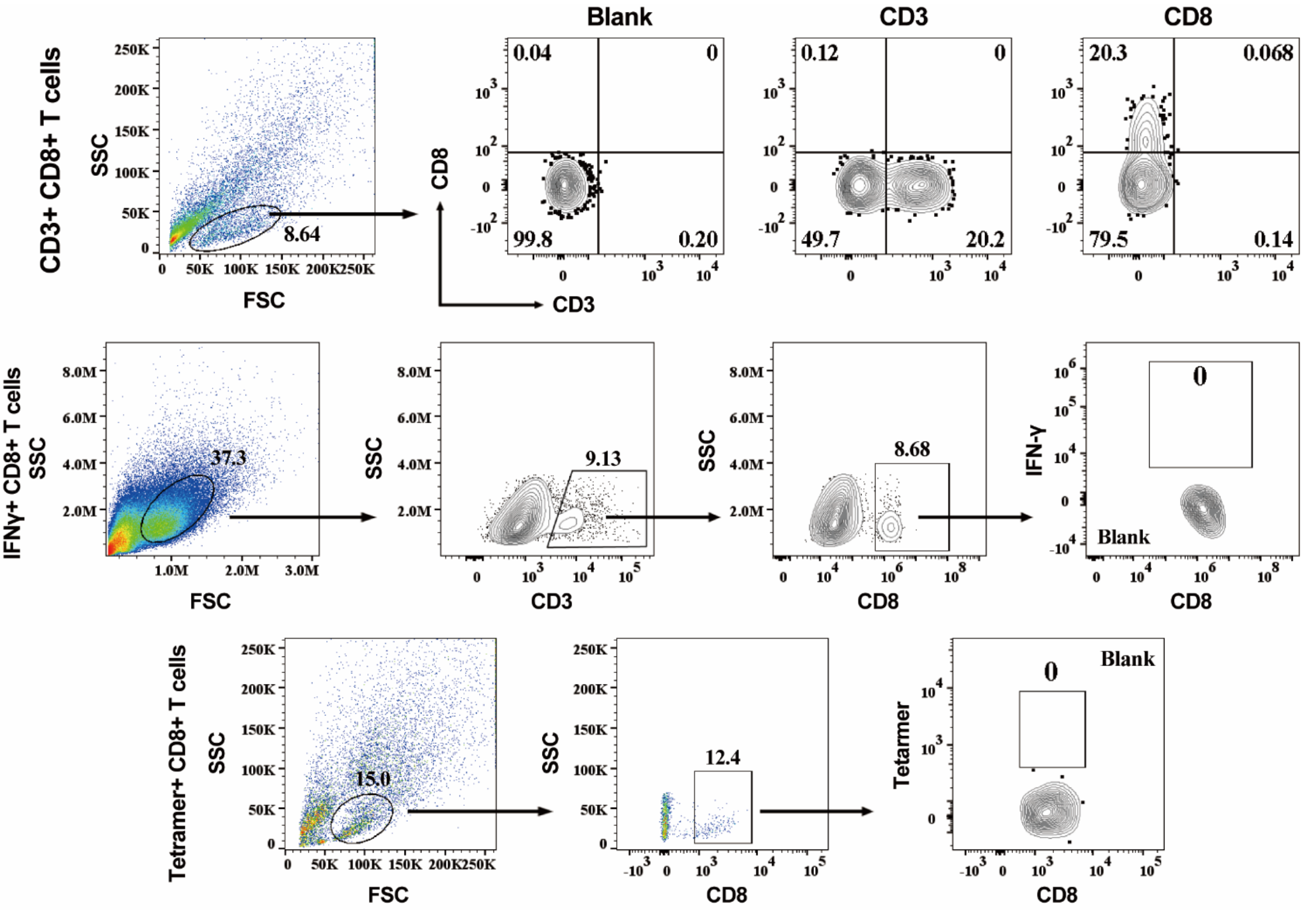

Figure S12. Flow cytometry gating strategy for analyzing T cell states within the tumor microenvironment. Related to Figure 3G.

Representative gating sequence used to identify and quantify intratumoral CD3+ CD8+ T cells, IFN-γ+ CD8+ T cells, and Ptpn2-specific CD8+ T cells in dissected tumor samples after STNvac treatment.

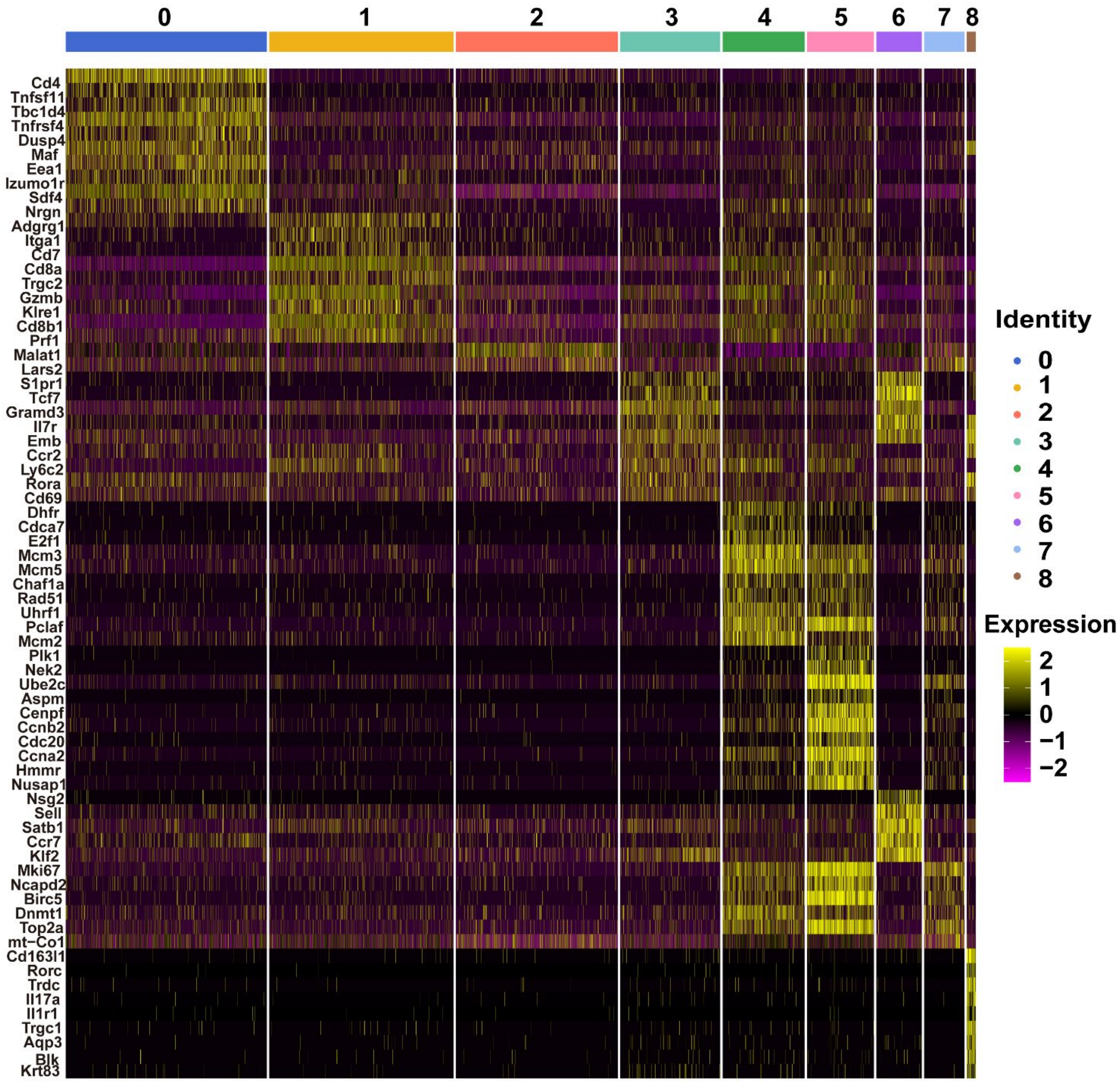

Figure S13. Heatmap showing gene expression profiles across T-cell clusters. Related to Figure 5C.
Representative heatmap illustrating the expression patterns of key marker genes across distinct T-cell clusters identified from single-cell transcriptomic analysis of PBS- and STNvac-treated tumors.

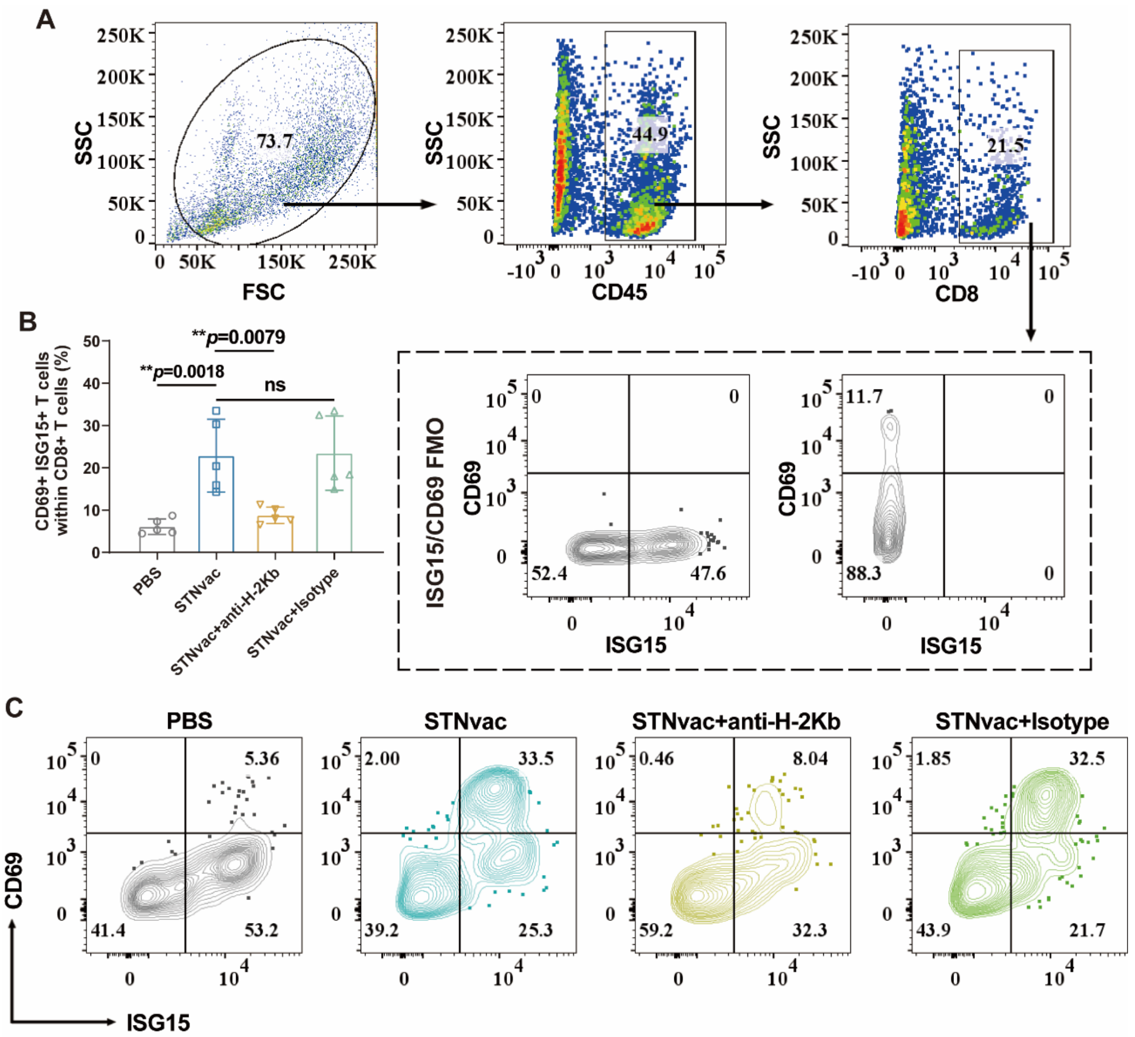

Figure S14. MHC-I blockade attenuates STNvac-induced enrichment of activated ISG15+ CD8+ T cells in tumors. Related to Figure 5.

For MHC-I blockade, anti-H-2Kb antibody or isotype control was administered intraperitoneally 24 h prior to each STNvac vaccination. Tumors were harvested 3 days after the final STNvac dose and processed into single-cell suspensions for flow cytometric analysis. An equal number of CD45+ events were acquired per sample.

(A) Gating strategy for the analysis of tumor-infiltrating lymphocytes. Fluorescence-minus-one (FMO) controls for ISG15 (secondary antibody included, primary antibody omitted) and CD69 were used to define positivity thresholds.

(B) Quantification of the frequency of ISG15+ CD69+ cells within tumor-infiltrating CD8+ lymphocytes across treatment groups, including PBS, STNvac, STNvac plus anti-H-2Kb antibody, and STNvac plus isotype control.

(C) Representative density plots of ISG15 and CD69 expressions within the CD45+ CD8+ population in each treatment group.

Statistics: One-way ANOVA. Data are presented as mean ± SD (n = 5 biological replicates). Significance levels: ** $p < 0.01$.

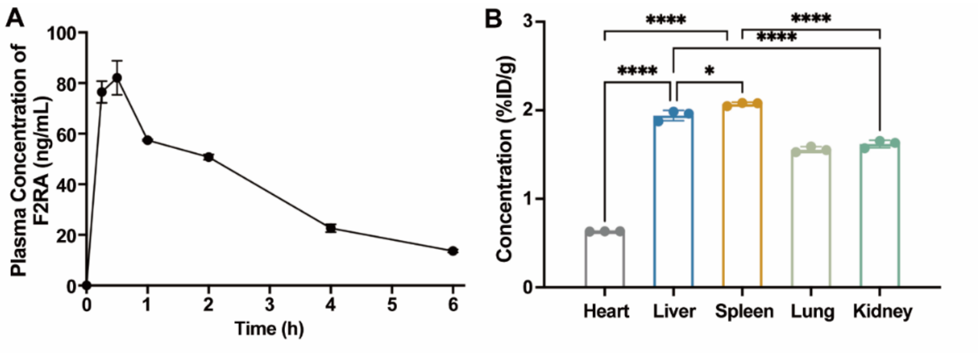

Figure S15. Pharmacokinetics and biodistribution of F2RA (SCH79797). Related to Figure 6.

(A) Plasma concentration-time profile of F2RA following intraperitoneal injection, measured by LC-MS/MS coupled with HPLC (Shimadzu UPLC 30AD with AB Sciex API5500 triple-quadrupole mass spectrometer). Plasma samples were collected at 0.25-6 h post-administration and analyzed by multiple-reaction monitoring (Q1/Q3 = 372.1/356.3 m/z) using a Shim-pack XR-ODS III C18 column (2.0 × 50 mm, 1.6 μm). Plasma levels peaked at 0.5 h, indicating rapid systemic absorption.

(B) Biodistribution of F2RA in major organs (heart, liver, spleen, lung, kidney) at 0.5 h post-injection, showing highest enrichment in the liver and spleen.

Statistics: One-way ANOVA for (B); Data are presented as mean ± SD (n = 3 biological replicates). Significance levels: $*p < 0.05$; $****p < 0.0001$.

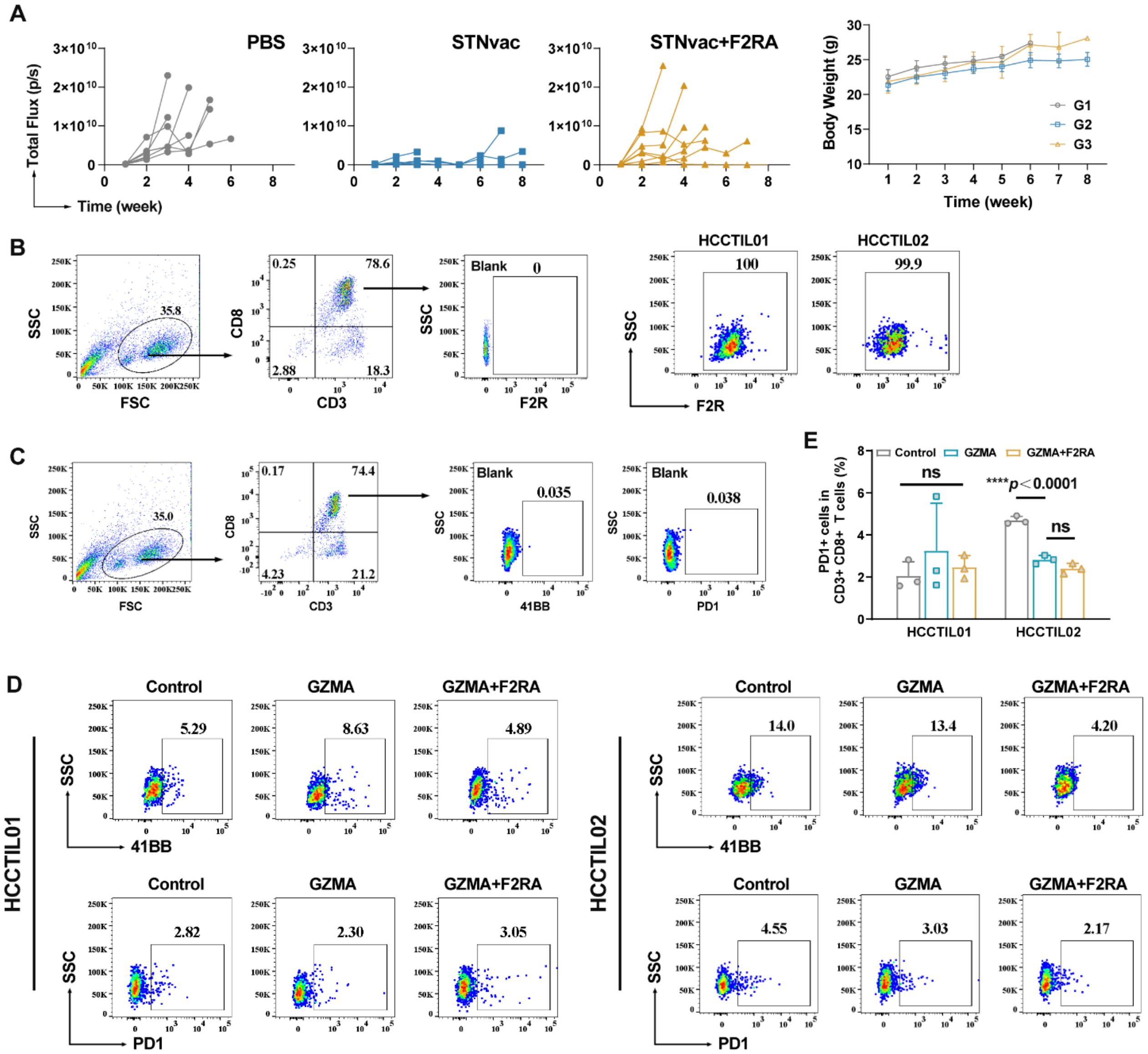

Figure S16. Effect of GZMA-F2R interaction on STNvac efficacy and human CD8+ TIL activation. Related to Figure 6.

(A) Individual tumor growth curves assessed by total fluorescence flux and mean body weight of the mice (related to Figure 6C).

(B) Flow cytometry analysis of F2R expression on CD3+ CD8+ TILs.

(C-E) Flow cytometry analysis of 41BB and PD1 expressions of the CD3+ CD8+ TILs after indicated administration (related to Figure 6H). (C) Gating strategies. (D) Representative scatter plots. (E) Quantitative analysis of 41BB+ and PD1+ CD3+ CD8+ T cell frequencies (n = 2 biological replicates, each analyzed in triplicate). Statistics: One-way ANOVA. Data are presented as mean ± SD. Significance levels: ****$p < 0.0001$.

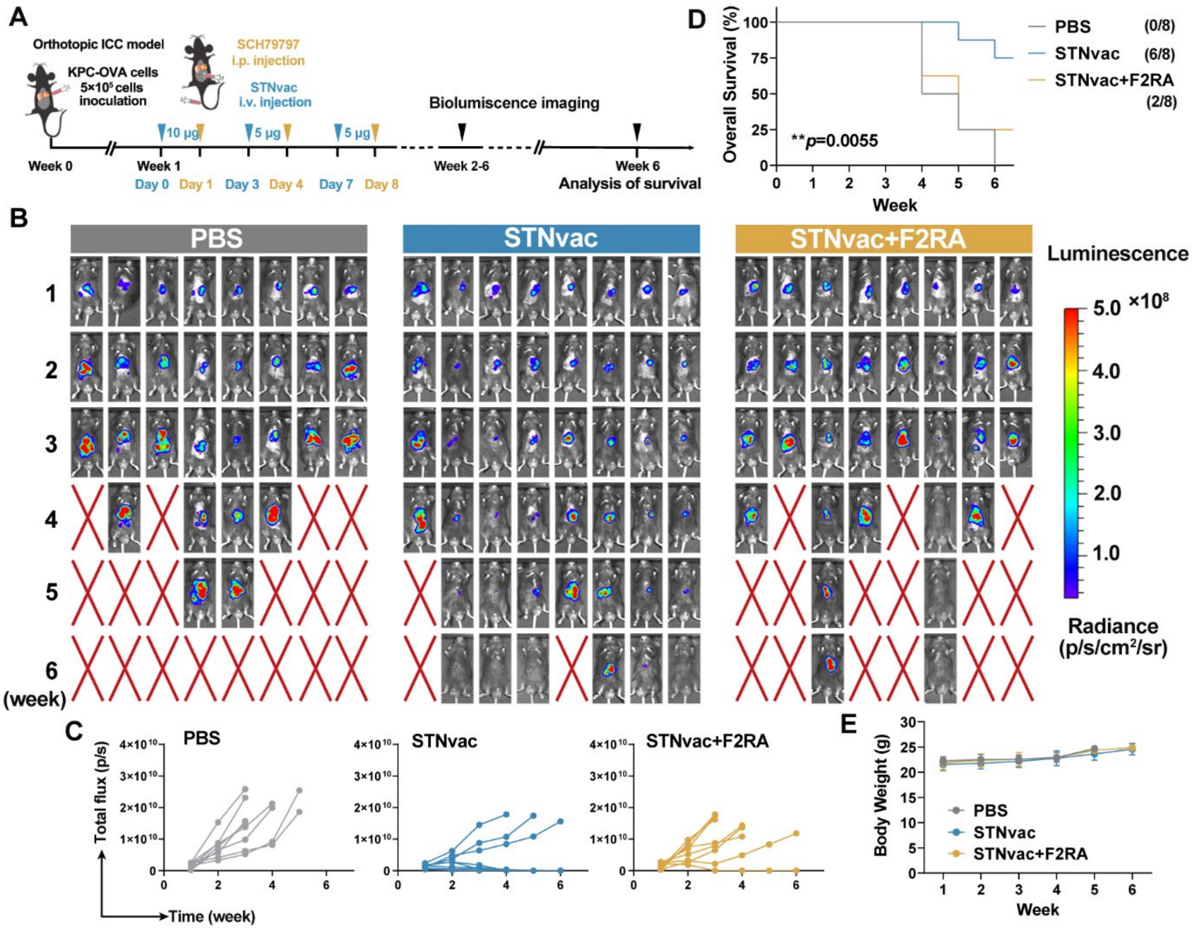

Figure S17. Validation of the GZMA-F2R pathway in an orthotopic intrahepatic cholangiocarcinoma (ICC) model derived from the KPC-OVA cell line. Related to Figure 6.

(A) Schematic illustration of the vaccination and treatment protocol. An orthotopic ICC model was established by intrahepatic implantation of KPC-OVA-Luc cells (KrasG12D; Trp53R172H; Pdx1-Cre) engineered to express the model antigen ovalbumin (OVA) and firefly luciferase (n = 8 mice per group). Mice received PBS, OVA-mRNA-loaded STNvac, or STNvac co-administered with the F2R antagonist (F2RA, SCH79797).

(B) Bioluminescence imaging showing tumor burden during the 6-week observation period. Co-administration of F2RA impaired the antitumor efficacy of OVA-STNvac, consistent with findings in the Hepa1-6 HCC model.

(C) Total bioluminescence flux for individual mice corresponding to (B).

(D) Survival curves of mice in different treatment groups, demonstrating reduced therapeutic benefit when the GZMA-F2R axis is blocked.

(E) Mean body weight of mice monitored throughout the study period, showing no significant systemic toxicity.

Statistics: Log-rank (Mantel-Cox) test for survival analysis (D). Significance levels: $^{**}p < 0.01$.

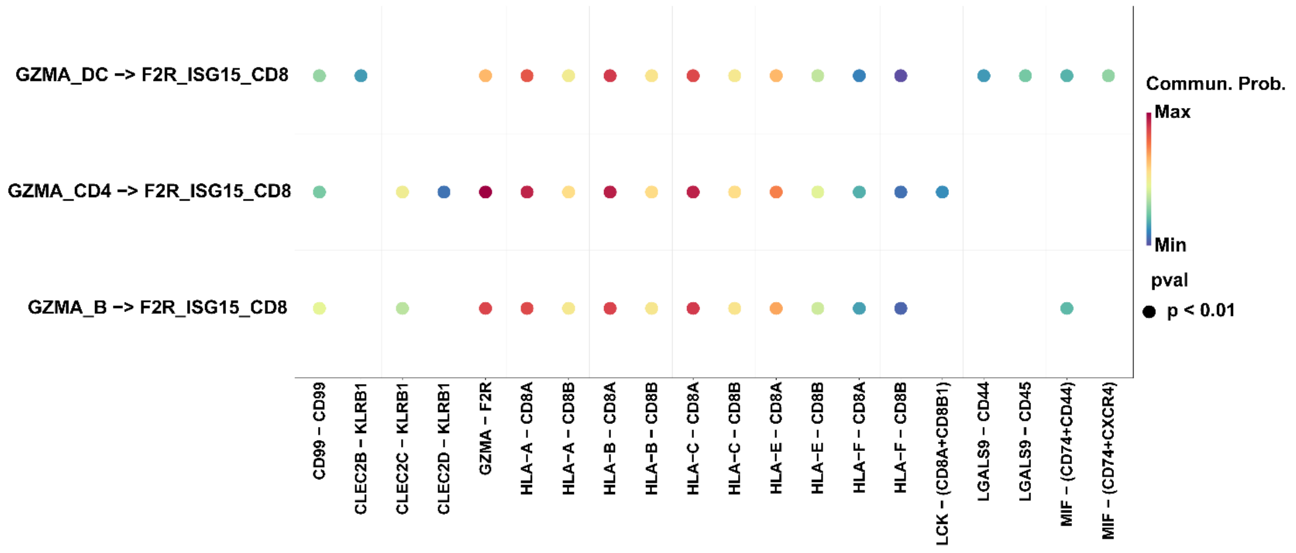

Figure S18. Predicted cell-cell interaction between F2R+ ISG15+ CD8+ T cells and GZMA+ APCs (DCs, B cells, CD4+ T cells) derived from scRNA-seq data of treatment-naive HCC patients (GEO: GSE156625). Related to Figure 6.

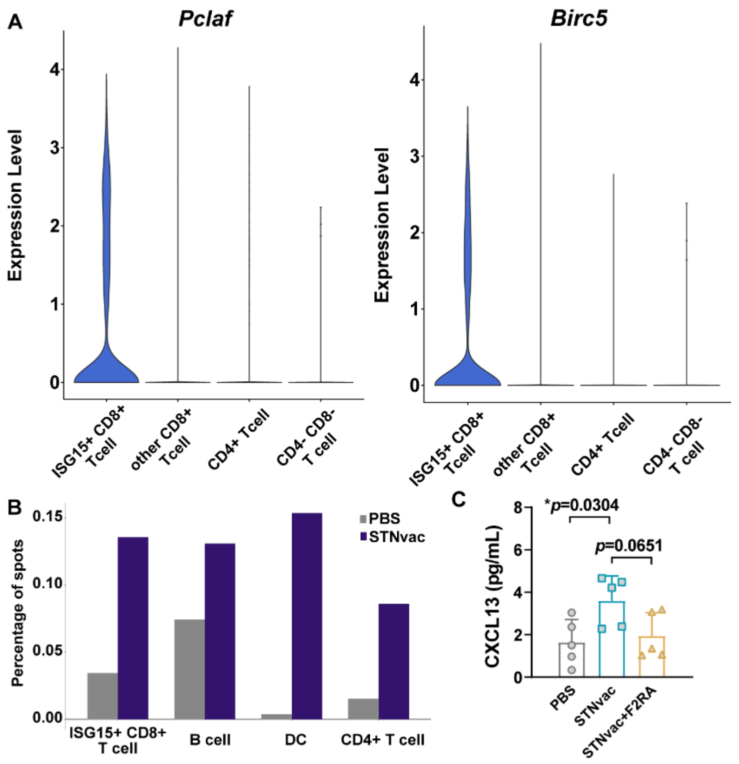

Figure S19. Spatial annotation of ISG15+ CD8+ T cells and CXCL13 expression. Related to Figures 6 and 7.

(A) Expression of ISG15+ CD8+ T cell-associated markers across T cell subsets in single-cell transcriptomic data. *Pclaf* and *Birc5* were selected as alternative signatures to represent ISG15+ CD8+ T cells in Figure 7C, as ISG15 was not included in the spatial probe list.

(B) Percentage of positive spatial transcriptomic spots annotated as ISG15+ CD8+ T cells, B cells, DCs, or CD4+ T cells in tumor tissues, corresponding to the regions shown in Figure 7C.

(C) ELISA analysis of CXCL13 in dissected tumors from PBS, STNvac and STNvac+F2RA groups, 3 days after the final treatment (related to Figure 6C). One-way ANOVA. Data are presented as mean ± SD (n = 5 biological replicates). Significance levels: *$p < 0.05$.

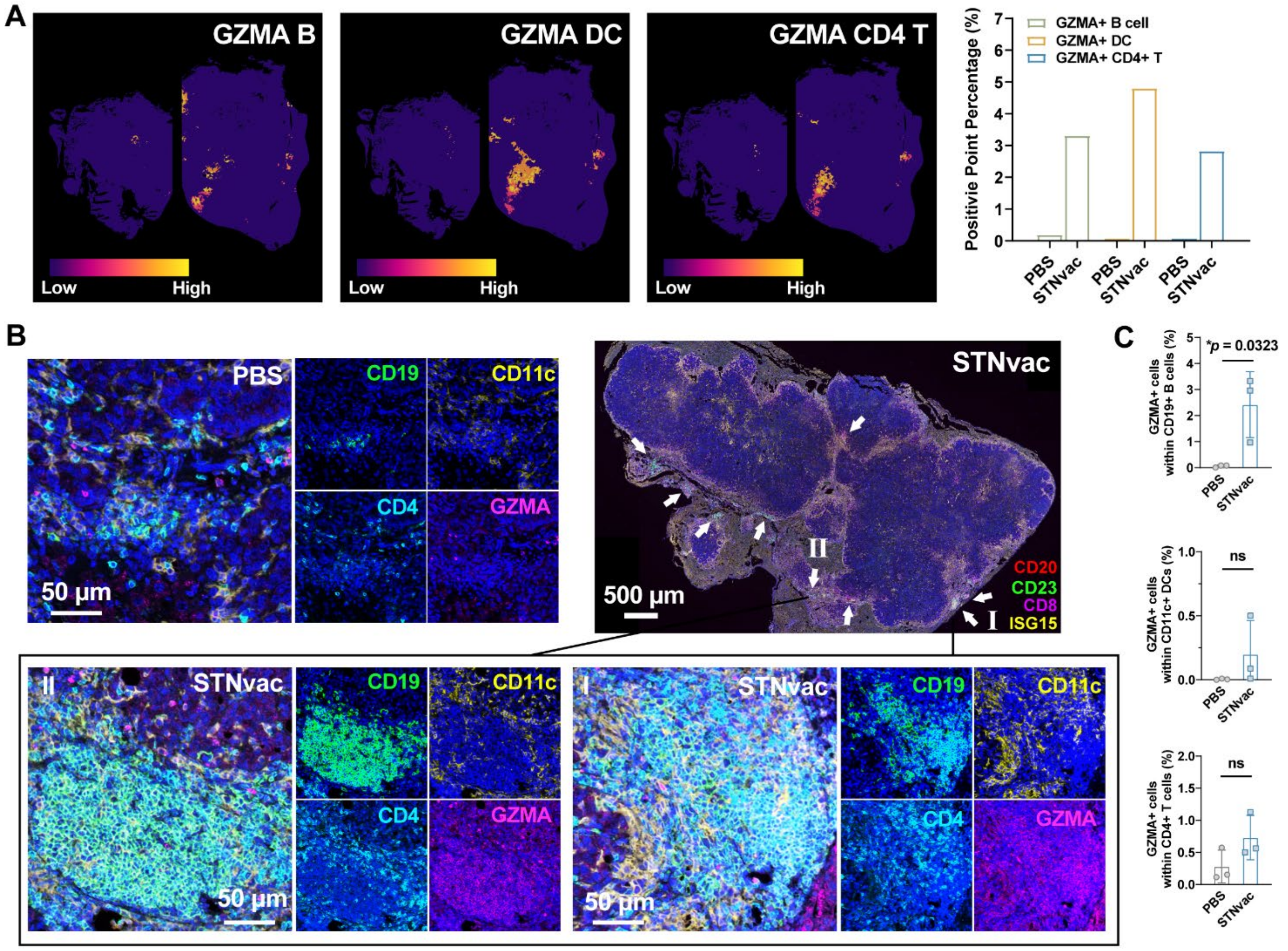

Figure S20. Spatial and histological validation of GZMA expression in APC subsets following STNvac treatment. Related to Figure 7.

(A) Spatial transcriptomic analysis of the same tumor sections shown in Figure 7A, quantifying the proportion of GZMA+ cells within B cells, DCs, and CD4+ T-cell populations.

(B) Multicolor immunofluorescence staining for CD19, CD11c, CD4, and GZMA in tumors from PBS- and STNvac-treated mice. The whole-section image of the STNvac group corresponds to that shown in Figure 7E, serving as a spatial reference to align consecutive sections. Regions I and II, indicated by white arrows, are displayed at higher magnification to illustrate the enrichment of GZMA+ APCs within and surrounding TLS regions. The PBS panel shows a magnified area corresponding to the arrow-indicated region in Figure 7E. Scale bars, 50 μm.

(C) Quantitative analysis of the percentage of GZMA+ cells within each APC subset (n = 3 biological replicates). Data are presented as mean ± SD. Statistical significance was determined by unpaired two-tailed Student's t-test (*$p < 0.05$).

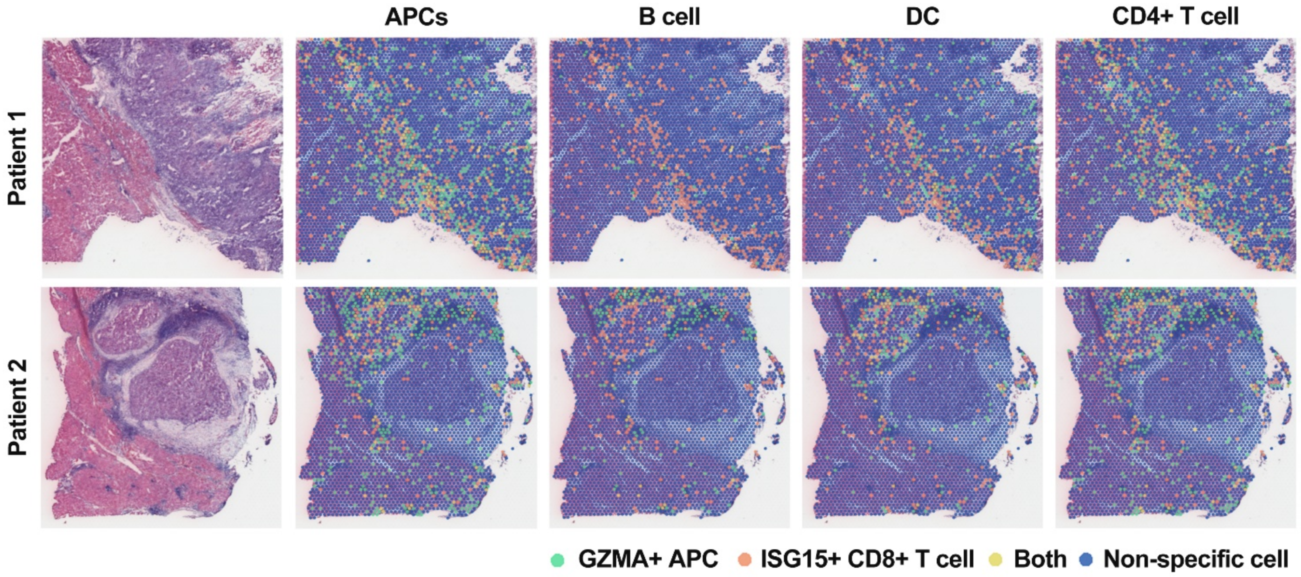

Figure S21. Spatial colocalization of ISG15+ CD8+ T cells and GZMA+ APCs in human HCC tissues. Related to Figure 7.

Spatial transcriptomic analysis of nine HCC samples from Mendeley Data: http://www.doi.org/10.17632/skrx2fz79n.1 (Liu's study; J. Hepatol., 2023, 78, 770, Ref 55). Two immune-inflamed tumors with pronounced immune-cell infiltration were selected for detailed evaluation. GZMA+ APCs (including GZMA+ DCs, B cells, and CD4+ T cells) were analyzed for their spatial relationship with ISG15+ CD8+ T cells, which were consistently localized around GZMA+ APCs at the tumor periphery, indicating conserved spatial organization between human and murine HCC.

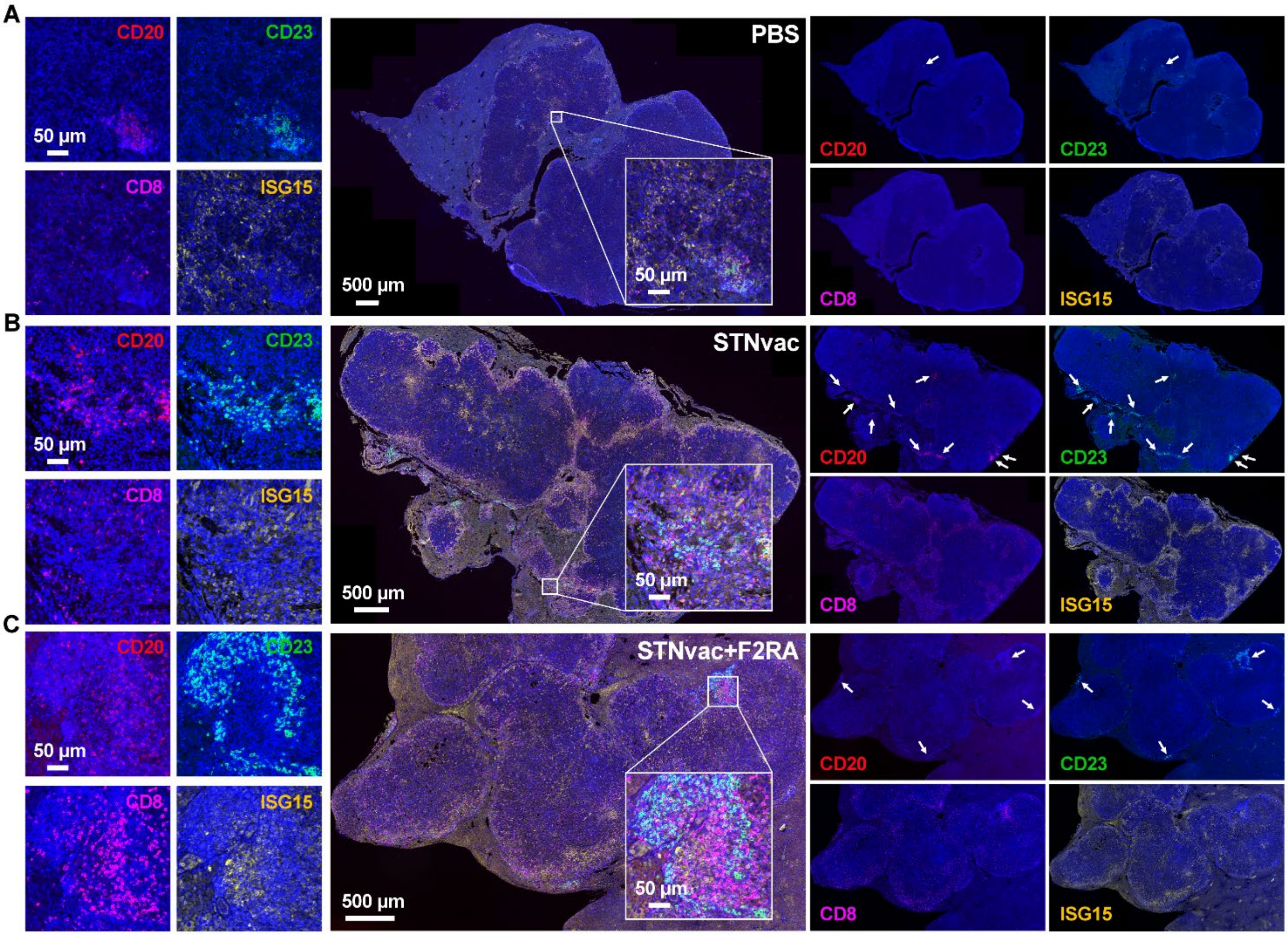

Figure S22. Representative multicolor and single-channel immunofluorescence images of TLSs and ISG15+ CD8+ T cells in tumors from different treatment groups. Related to Figure 7.

(A-C) Tumor sections from (A) PBS, (B) STNvac, and (C) STNvac+F2RA groups stained for CD20, CD23, CD8, and ISG15. Shown are representative composite and single-channel views, as well as higher-magnification images of TLS regions corresponding to those in Figure 7E.

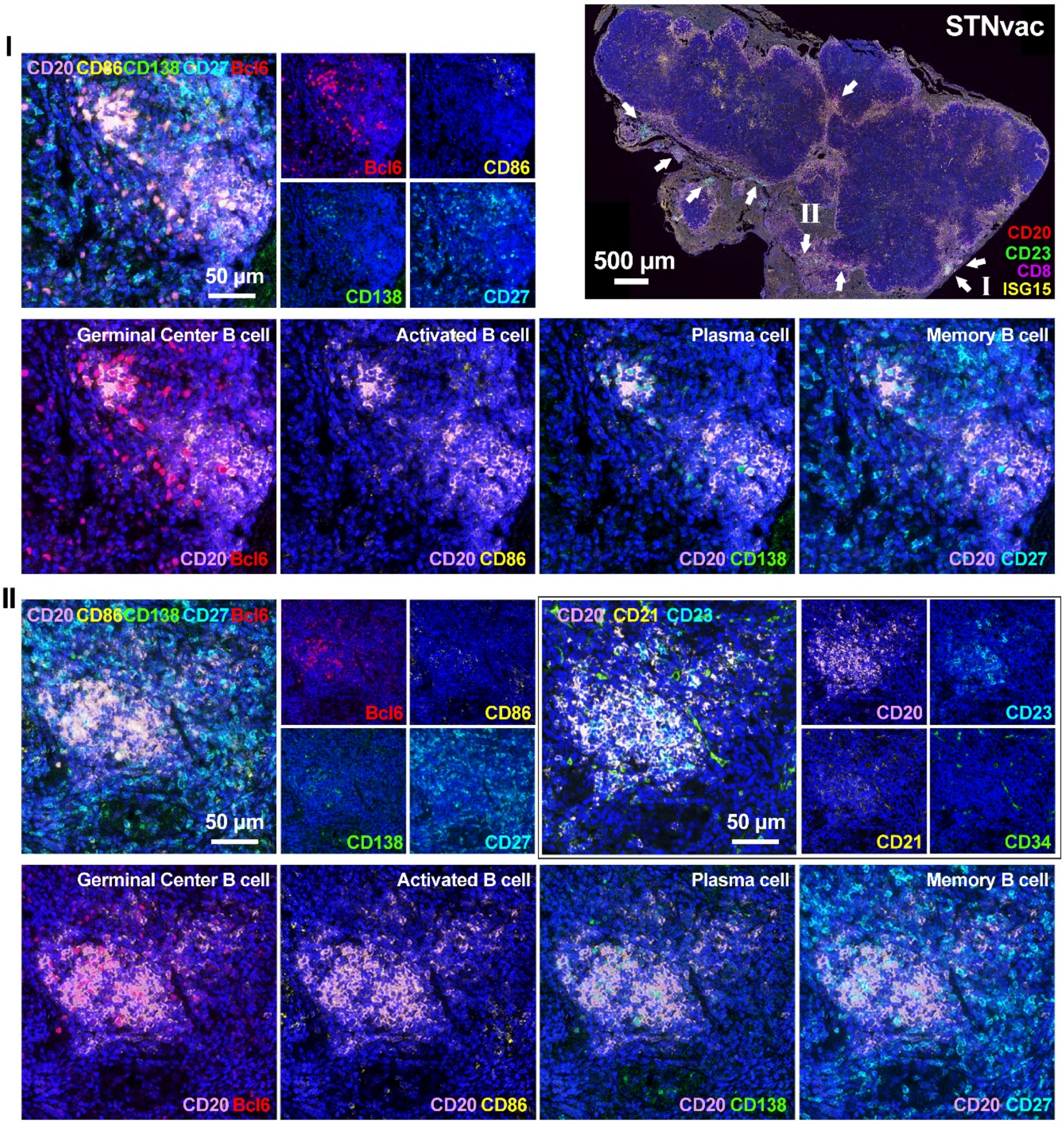

Figure S23. Detailed characterization of B-cell maturation and TLS organization in STNvac-treated tumors. Related to Figure 7.

Tumor sections from the STNvac group (consecutive to the sample shown in Figure 7E) were analyzed by multicolor immunofluorescence. The whole-section image corresponds to Figure 7E and serves as a spatial reference for aligning consecutive sections. Regions I and II are shown at higher magnification. Scale bars, 50 μm.

Region I: Five-marker staining (CD20, Bcl6, CD86, CD138, and CD27) revealed distinct B-cell subsets within TLSs. Representative merged and single-channel images are presented, together with enlarged dual-marker views highlighting CD20+ Bcl6+ germinal-center B cells, CD20+ CD86+ activated B cells, CD20- CD138+ plasma cells, and CD20+ CD27+ memory-like B cells.

Region II: The same five-color panel as Region I, together with an additional four-marker panel (CD20, CD21, CD23, and CD34), illustrates CD21+ FDC networks and CD34+ HEV-like structures at TLS boundaries.

Similar B-cell subsets, as well as CD21+ and CD34+ structures were consistently observed in additional biological replicates (data not shown), confirming the reproducibility of TLS organization.

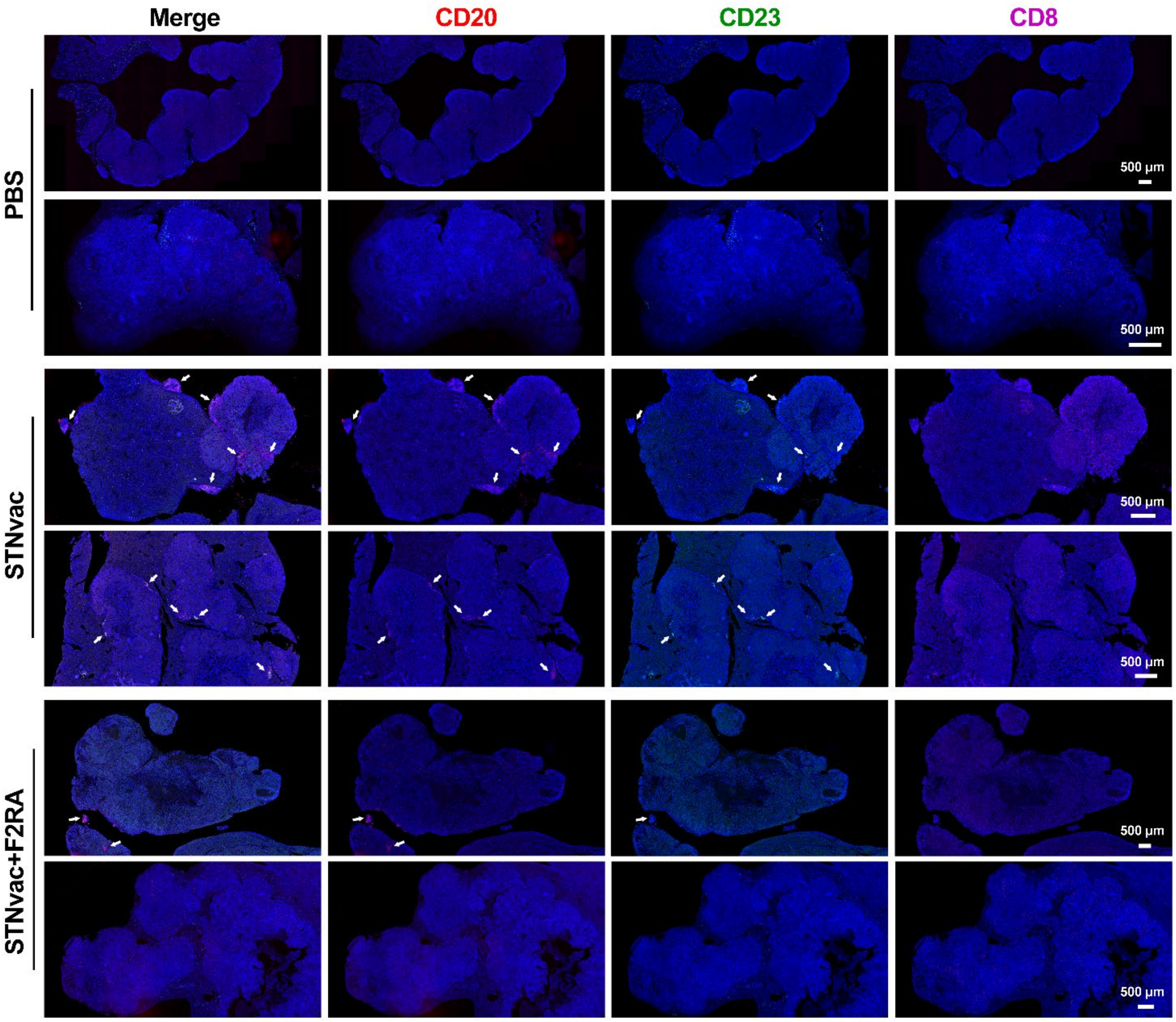

Figure S24. Multicolor immunofluorescence validation and quantitative assessment of TLSs across biological replicates. Related to Figure 7G.

Representative CD20/CD23 dual-staining images from PBS-, STNvac-, and STNvac+F2RA-treated tumors. Shown are the two additional biological replicates not depicted in Figure 7E and Figure S21; together, these three tumors were used for TLS quantification in Figure 7G. For quantification, TLSs were defined as organized CD20+ CD23+ lymphoid aggregates consistent with secondary follicular TLSs described in the literature (Science, 2022, 2528, eabf9419; Cancer Res, 2018, 78, 1308; Immunity, 2022, 55, 527), and only aggregates with a longest axis greater than 100 μm were counted to exclude small, non-structured B-cell clusters. This unified definition was used for TLS quantification in Figure 7G. In the STNvac+F2RA group, CD20+ aggregates lacking CD23 signal were observed and were therefore not classified as mature TLSs in this study. Scale bars, 500 μm.

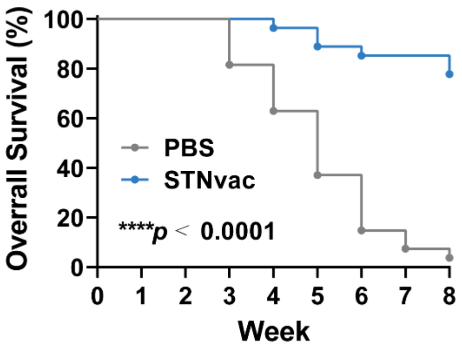

Figure S25. Pooled survival analysis of STNvac across independent orthotopic HCC experiments. Related to Figures 3 and 6.

Kaplan-Meier survival data were combined from four independent orthotopic HCC studies (Figures 3D, 6D, S6D, and S8D), each including PBS and STNvac treatment groups (total n = 27 mice per group). STNvac significantly improved overall survival compared with PBS (77.8% vs 3.7%; log-rank test, ****$p < 0.0001$), confirming the reproducibility and robust efficacy of the vaccine across independent cohorts.

Table S1. The mRNA sequence of Hepa1-6/LLC neoantigens for neoantigen vaccine preparation. Related to STAR Methods.

**Hepa 1-6 neoantigen mRNA sequence**
ATGGCCGTCATGGCGCCCCGAACCCTCCTCCTGCTACTCTCGGGGGCCCTGGCCCTGACCCAGACCTGGG<br>CGGGCTCCATGAAGGCCCGGAACTACCTGCAGTTCCTGCCCAGCAAGACCAAGGTGGCCCGGGGCCGG<br>AAGCGGCGGAGCCGGGGCGAGCACTACCGGTACAAGGTGAGCCTGCCCGGCGGCCAGCACGCCCGGGG<br>CCGGAAGCGGCGGAGCCACGTGCTGTGGGACCTGAAGCAGATGTTCCGGTGCGCCGTGCTGAAGAACC<br>GGGGCCGGAAGCGGCGGAGCTGGGACACCTGCACCACCTACAAGTGGCAGAAGACCCTGGAGGGCCAC<br>GACCGGGGCCGGAAGCGGCGGAGCCTGAGCACCTACCGGACCGCCTGCACCCTGCGGTTCGTGCAGAA<br>GCGGTGCCGGGGCCGGAAGCGGCGGAGCCTGTACACCCACTTCCTGCAGCTGCCCCTGGCCGCCACCGG<br>CTTCAGCGTGCGGGGCCGGAAGCGGCGGAGCAAGCGGTGGCTGTACTGGCAGCCCACCCTGACCAAGA<br>TGGGCTTCGTGAGCCGGGGCCGGAAGCGGCGGAGCGGATCCATCATTGCTGGCCTGGTTCTCCTTGGAG<br>CTGTGATCACTGGAGCTGTGGTCGCTGCCGTGATGTGGAGGAGGAAGAGCTCAGATAGAAAAGGAGGG<br>AGTTACACTCAGGCTGCAAGCAGTGACAGTGCCCAGGGCTCTGATGTGTCCCTCACAGCTTGTAAAGTG<br>TGATAA
**LLC neoantigen mRNA sequence**
ATGGCCGTCATGGCGCCCCGAACCCTCCTCCTGCTACTCTCGGGGGCCCTGGCCCTGACCCAGACCTGGG<br>CGGGCTCCCCCGGCGCCGCCCCCGTGGTGGTGGCCCACGCCCGGCTGAGCCGGGTGCTGCGGGGCCGGA<br>AGCGGCGGAGCCCCTACAAGTGCACCAAGTGCGGCCGGGCCTTCACCCGGAGCAGCACCCTGCGGGGC<br>CGGAAGCGGCGGAGCAACCTGACCCTGCACTACCGGACCCTGCTGGTGGACCGGCCCTACGACTGCCGG<br>GGCCGGAAGCGGCGGAGCGACCCCTGGGCCGGCTACCGGTACAGCGGCAAGCTGCGGCCCCACTACCC<br>CCGGGGCCGGAAGCGGCGGAGCGAGGAGGACACCGACACCAAGCAGATCTACTTCTACCTGTTCAAGC<br>TGCTGCGGGGCCGGAAGCGGCGGAGCCTGTTCGGCCTGATCAACGTGACCCCCAACATGCTGCCCCTGG<br>TGAAGAAGCGGGGCCGGAAGCGGCGGAGCGCCGGCAACCGGCACATCAAGTTCTGCTACCTGGACGAC<br>AGCAAGACCAGCCGGGGCCGGAAGCGGCGGAGCATCATTGCTGGCCTGGTTCTCCTTGGAGCTGTGATC<br>ACTGGAGCTGTGGTCGCTGCCGTGATGTGGAGGAGGAAGAGCTCAGATAGAAAAGGAGGGAGTTACAC<br>TCAGGCTGCAAGCAGTGACAGTGCCCAGGGCTCTGATGTGTCCCTCACAGCTTGTAAAGTGTGATAA

Note: The blue font represents the signal peptide and MITD structure, the red font represents the neoantigen sequence, and the green font represents the Furin sequence.
