# Supplementary material for: Spleen-targeted neoantigen mRNA vaccine induces ISG15+ CD8+ T cell-mediated tertiary lymphoid structure formation in hepatocellular carcinoma
Source: Cell Rep Med. 2026 Apr 20;7(5):102754. doi: 10.1016/j.xcrm.2026.102754 (PMC13198314; doi:10.1016/j.xcrm.2026.102754)
Supplement: Document S2. Article plus supplemental information [file mmc2.pdf]

Cell Reports
Medicine

Article

Spleen-targeted neoantigen mRNA vaccine induces ISG15$^+$ CD8$^+$ T cell-mediated tertiary lymphoid structure formation in hepatocellular carcinoma

Graphical abstract

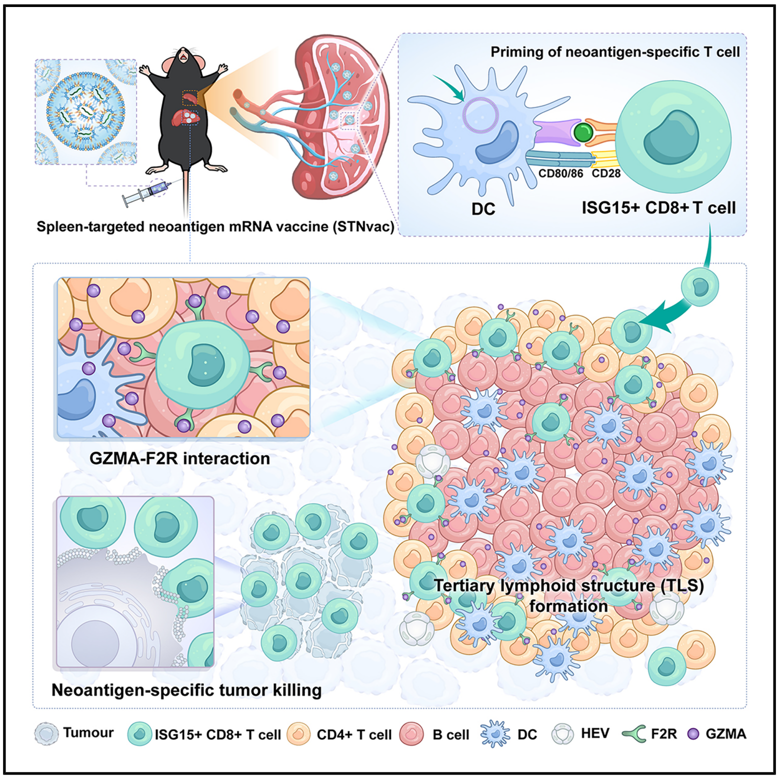

Authors

Xinyi Lin, Geng Chen, Ruijing Tang, ..., Haijun Yu, Zhixiong Cai, Xiaolong Liu

Correspondence

caizhixiong1985@163.com (Z.C.),
xiaoloong.liu@gmail.com (X.L.)

In brief

In this study, Lin et al. develop a spleen-targeted neoantigen mRNA vaccine (STNvac) for hepatocellular carcinoma. STNvac induces neoantigen-specific ISG15$^+$ CD8$^+$ T cells and promotes their activation and proliferation via GZMA-F2R interactions with antigen-presenting cells, supporting TLS formation and coordinated antitumor immunity.

Highlights

- Spleen-targeted neoantigen mRNA vaccine elicits potent antitumor immunity in HCC
- STNvac induces neoantigen-specific ISG15$^+$ CD8$^+$ T cells as key effectors
- GZMA-F2R signaling promotes ISG15$^+$ CD8$^+$ T cell activation and TLS formation

Lin et al., 2026, Cell Reports Medicine 7, 102754
May 19, 2026 © 2026 The Authors. Published by Elsevier Inc.
https://doi.org/10.1016/j.xcrm.2026.102754

CellPress

Cell Reports Medicine

CellPress
OPEN ACCESS

Article

Spleen-targeted neoantigen mRNA vaccine induces ISG15^{+} CD8^{+} T cell-mediated tertiary lymphoid structure formation in hepatocellular carcinoma

Xinyi Lin,[1,2,3,5] Geng Chen,[1,2,3,5] Ruijing Tang,[1,2,3,5] Ming Wu,[1,2,3,5] Da Zhang,[1,2,3,5] Fangzhou Lin,[1,2,3] Jianhua Guan,[1,2,3] Jing Yang,[1,2,3] Xiuqing Dong,[1,2,3] Xiaoyuan Zheng,[1,2,3] Liman Qiu,[1,2,3] Haijun Yu,[4] Zhixiong Cai,[1,2,3,*] and Xiaolong Liu[1,2,3,6,*]

[1]The United Innovation of Mengchao Hepatobiliary Technology Key Laboratory of Fujian Province, Mengchao Hepatobiliary Hospital of Fujian Medical University, Fuzhou, Fujian 350007, P.R. China

[2]Mengchao Med-X Center, Fuzhou University, Fuzhou, Fujian 350116, P.R. China

[3]The Liver Center of Fujian Province, Fujian Medical University, Fuzhou, Fujian 350007, P.R. China

[4]State Key Laboratory of Drug Research & Center of Pharmaceutics, Shanghai Institute of Materia Medica, Chinese Academy of Sciences, Shanghai 201203, China

[5]These authors contributed equally

[6]Lead contact

*Correspondence: caizhixiong1985@163.com (Z.C.), xiaoloong.liu@gmail.com (X.L.)

https://doi.org/10.1016/j.xcrm.2026.102754

SUMMARY

The efficacy of neoantigen vaccine for advanced hepatocellular carcinoma (HCC) is limited largely due to insufficient T cell mobilization and activation. Herein, we develop a spleen-targeted neoantigen mRNA vaccine (STNvac) with highly efficient spleen-selective mRNA transfection. Using a three-dose vaccination regimen, STNvac demonstrates remarkable therapeutic efficacy in orthotopic HCC model with a high likelihood of complete tumor regression and significantly improved survival rates ($p < 0.0001$). Notably, we identify a distinct ISG15^{+} CD8^{+} T cell population as crucial mediators of STNvac-induced immunity with potent antigen-processing and cytotoxic capacities. Intriguingly, STNvac promotes the formation of tertiary lymphoid structures (TLSs) through GZMA-F2R-mediated interactions between ISG15^{+} CD8^{+} T cells and antigen-presenting cells (APCs), which is also confirmed in HCC patients. Taken together, our findings demonstrate the potent antitumor efficacy of spleen-targeted mRNA vaccine and reveal its underlying immune cell interactive mechanisms, presenting high potential for clinical translation.

INTRODUCTION

Cancer immunotherapy has transformed the management of many malignancies, yet hepatocellular carcinoma (HCC) remains largely immune-refractory. Its low-to-moderate tumor mutation burden (TMB) and immunologically “cold” tumor microenvironment (TME) underlie poor response rates to programmed cell death protein 1 (PD-1)/programmed death-ligand 1 (PD-L1) blockade monotherapy in advanced HCC (<20%).[1–4] These unsatisfactory outcomes are largely attributed to insufficient T cell infiltration and limited antigen-recognition capacity.[5–7] Personalized neoantigen vaccines, incorporating patient-specific tumor antigens, offer a promising strategy to address these challenges.[8–10] Such vaccines effectively elicit and expand tumor-specific T cell responses without off-target toxicity, showing clinical benefits in melanoma,[11] glioblastoma,[12] and non-small-cell lung cancer (NSCLC).[13] Our group and others have validated the feasibility and efficacy of peptide- and DNA-based neoantigen vaccines in HCC, confirming their capacity to induce neoantigen-specific T cells and enhance intratumoral infiltration.[6,14–20] Nonetheless, the magnitude and durability of these T cell responses remain suboptimal.

Messenger RNA (mRNA) vaccines have achieved remarkable success during the COVID-19 pandemic, demonstrating strong potential as next-generation personalized cancer vaccines.[21–23] Compared with conventional platforms, mRNA vaccines elicit stronger humoral and cellular immune responses and can encode multiple antigen sequences simultaneously, allowing flexible design to enhance immunogenicity.[24] Their efficacy, however, critically depends on rationally engineered lipid nanoparticles (LNPs) that enable efficient antigen expression in target cells. With local administration routes such as intramuscular (i.m.) or subcutaneous (s.c.) injection, most mRNA vaccines are taken up by myocytes or keratinocytes rather than professional antigen-presenting cells (APCs), leading to suboptimal immune activation.[25,26] Targeting organs rich in APCs is therefore essential for effective mRNA vaccination. As the largest secondary lymphoid organ, the spleen represents an ideal target for systemic (intravenous [i.v.]) delivery. Recent studies have developed LNP formulations capable of spleen-selective

© 2026 The Authors. Published by Elsevier Inc.
This is an open access article under the CC BY license (http://creativecommons.org/licenses/by/4.0/).

mRNA translation.[27–32] The spleen-targeted formulations markedly enhance antigen presentation by splenic APCs and promote antigen-specific T cell infiltration, leading to potent antitumor effects in melanoma, lymphoma, and colorectal carcinoma models.[33–35] Early-stage clinical trials have further shown promising efficacy in patients with unresectable melanoma resistant to checkpoint inhibitors, underscoring the therapeutic potential of spleen-targeted mRNA vaccines for treating advanced and immune-resistant tumors. Nevertheless, their feasibility and efficacy in delivering highly immunogenic neoantigens for aggressive HCC, as well as the underlying molecular mechanisms shaping the HCC TME, remain largely unexplored.

Although the quantity and quality of tumor-infiltrating immune cells are key indicators of antitumor immunity, accumulating evidence highlights that their localization, spatial organization, and intercellular interactions are equally crucial.[36,37] However, these spatial aspects remain poorly understood in the context of HCC neoantigen and spleen-targeted mRNA vaccines. Tumor-infiltrating immune cells can form organized tertiary lymphoid structures (TLSs), which are associated with better prognosis and improved immunotherapy outcomes across various malignancies,[36–40] including HCC.[41] Antigen-specific immune cells have been shown to produce molecular mediators required for TLS induction, and sustained antigen recognition is essential for TLS maintenance.[37,42,43] While spleen-targeted mRNA vaccines effectively prime antigen-specific T cells and enhance their tumor infiltration, whether they can trigger TLS formation in immune-resistant HCC remains unclear. In particular, the spatiotemporal coordination of vaccine-educated immune populations within TLS niches, especially the dynamic crosstalk between antigen-specific T cells and APCs, represents a pivotal yet understudied dimension of HCC immunotherapy. Deciphering the molecular mechanisms driving vaccine-induced TLS formation and immune landscape reorganization is crucial for advancing innovative, spatially targeted immunotherapeutic strategies for HCC.

Here, we developed a spleen-targeted neoantigen mRNA vaccine (STNvac) designed to elicit robust and durable antitumor immunity against HCC (Figure 1A). STNvac employs a simplified two-component lipid nanoparticle (LNP) formulation optimized for selective spleen delivery, enabling efficient activation of splenic DCs and robust priming of neoantigen-specific T cells. In orthotopic HCC models, STNvac induced strong antitumor immune responses, leading to marked tumor regression. Integrated multicolor immunofluorescence, single-cell, and spatial transcriptomic analyses revealed a distinct $ISG15^+$ $CD8^+$ T cell subset that emerged following vaccination and interacted with intratumoral APCs through a GZMA-F2R signaling axis. STNvac treatment markedly promoted the formation of TLS at the tumor margin, where $ISG15^+$ $CD8^+$ T cells and APCs showed spatial co-localization and evidence of GZMA-F2R signaling. Functional analyses further demonstrated that this pathway contributes to T cell activation, proliferation, and TLS organization in vaccinated mice, and the same signaling interaction was also observed in HCC specimens. Collectively, these findings demonstrate the therapeutic potential of STNvacs for treating immune-resistant solid tumors and provide mechanistic insights into the spatial organization and cellular crosstalk of vaccine-induced tumor-infiltrating immune cells.

RESULTS

Synthesis and characterization of spleen-targeted LNP-mRNA

To facilitate clinical translation and simplify preparation, a two-component LNP was synthesized using the cationic lipid DOTMA and the helper phospholipid DOPE.[27] Compared with more complex formulations requiring RNA incorporation during particle assembly or additional lipid components, DOTMA/DOPE enables a convenient mix-and-use strategy, making it suitable for rapid and personalized mRNA vaccine production. The as-synthesized LNPs exhibited monodisperse vesicular morphology (Figures 1A–1C) and maintained stable size and dispersity during long-term storage at 4°C (Figures S1A–S1C), confirming good colloidal stability. Upon mRNA complexation, LNP-mRNA formed solid spherical morphology with a moderate size increase (Figures 1D, 1E, and S1D), and successful RNA encapsulation was confirmed by gel electrophoresis (Figure S1E). Under serum challenge, DOTMA/DOPE LNPs exhibited gradual mRNA release and degradation, with most RNAs remaining protected within the first 6 h (Figures S1F and S1G). Given that particle surface charge influences spleen targeting and transfection efficiency,[27,30–32] we systematically varied the LNP/mRNA ratios (denoted as the nitrogen [N] in DOTMA to phosphate [P] in mRNA ratio) to evaluate the effect of particle charge on *in vitro* transfection and *in vivo* spleen selectivity. LNP-mRNAs were negatively charged at $N/P \leq 1$ and became positively charged at $N/P \geq 1.5$ (Figure 1F). *In vitro*, LNP-mRNAs exhibited good biocompatibility (Figure S1H) and efficiently transfected 293T and DC2.4 cells, with peak efficiency observed at N/P = 2 (Figure S2). These results demonstrate that DOTMA/DOPE LNP enables stable mRNA encapsulation and efficient expression in dendritic cells (DCs).

The spleen, the largest secondary lymphoid organ enriched with DCs, serves as a major site for T cell priming and antigen-specific immune activation. Spleen-selective transfection is thus a desirable feature for intravenously administered mRNA neoantigen vaccines, as it enables antigen expression in DCs while minimizing off-target effects.[19,29] To optimize spleen-selective transfection *in vivo*, luciferase-encoding mRNA ($mRNA^{Fluc}$) was employed as a reporter to evaluate biodistribution following intravenous administration. As shown in Figures 1G–1I, luciferase expression was predominantly observed in the spleen across all N/P ratios, with the N/P = 0.5 group exhibiting the strongest and most selective signal (>99%). This distribution suggests that DOTMA/DOPE LNPs intrinsically favor spleen accumulation, which is further enhanced at low N/P ratios with mildly negative surface charge. Collectively, these results indicate that LNP-mRNA with N/P ratio of 0.5 displays superior spleen-targeting efficiency *in vivo* and can serve as a promising formulation for spleen-targeted neoantigen vaccines.

STNvac elicits antigen-specific immune responses in the spleen

DCs, as the most potent APCs, play a vital role in initiating T-cell-mediated antigen-specific immune responses and are therefore a crucial target for neoantigen vaccines (Figure 2A). To determine whether LNP-mRNA effectively transfects splenic DCs,

CellPress
OPEN ACCESS

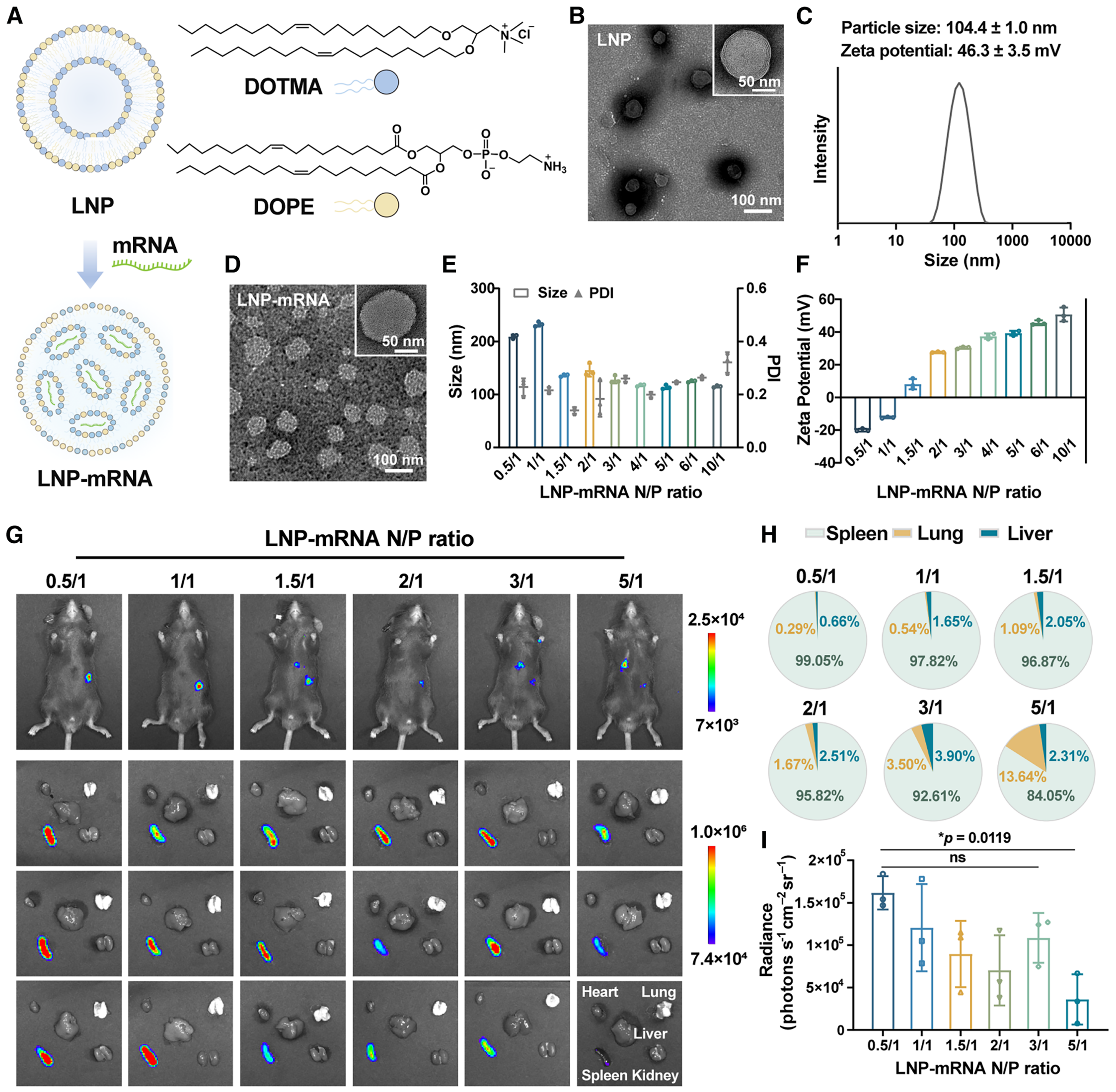

Figure 1. Synthesis and characterization of LNP-mRNA

(A) Schematic illustration of STNvac formulation composed of the cationic lipid DOTMA and helper phospholipid DOPE mixed with mRNA encoding the murine HCC neoantigens.

(B) Transmission electron microscopy (TEM) image of DOTMA/DOPE LNPs before mRNA loading, showing uniform vesicular morphology. Scale bars, 100 nm; 50 nm (inset).

(C) Particle size distribution of as-synthesized LNPs.

(D) TEM image of LNP-mRNA showing solid spherical morphology after mRNA complexation. Scale bars, 100 nm; 50 nm (inset).

(E) Hydrodynamic size of LNP-mRNA at different N/P ratios (110–240 nm) (n = 3 independent preparations).

(F) Zeta potential of LNP-mRNA at different N/P ratios (n = 3 independent preparations).

(G–I) *In vivo* biodistribution of mRNA expression 6 h after intravenous administration of LNP-mRNA^{Fluc} at various N/P ratios. (G) Bioluminescence imaging of treated C57BL/6 mice and the *ex vivo* major organs (heart, liver, spleen, lung, and kidney). (H) Pie charts showing the relative contribution of spleen, lung, and liver to the total luminescence signal. (I) Quantification of bioluminescence intensity in the spleen across different N/P ratios.

One-way ANOVA; $^*p < 0.05$. Mean ± SD (n = 3 biological replicates). See also Figures S1 and S2.

luciferase mRNA (mRNA^{Fluc}) was employed as a reporter to analyze mRNA expression among immune subsets in the spleen. Flow-cytometric profiling revealed that although DCs accounted for only a small fraction of splenic immune cells (3.42%), they exhibited the highest luciferase expression (24.97%), indicating preferential transfection of professional APCs by DOTMA/DOPE LNPs (Figure S3). To further assess mRNA uptake and localization, Cy5-labeled mRNA (mRNA^{Cy5}) was used to visualize LNP internalization in splenic tissue sections. As shown in Figure 2B, in PBS controls, CD11c$^+$ DCs were sparse and rarely present in the white pulp (T cell zone, 16.2%), whereas LNP-mRNA administration markedly increased overall CD11c signal

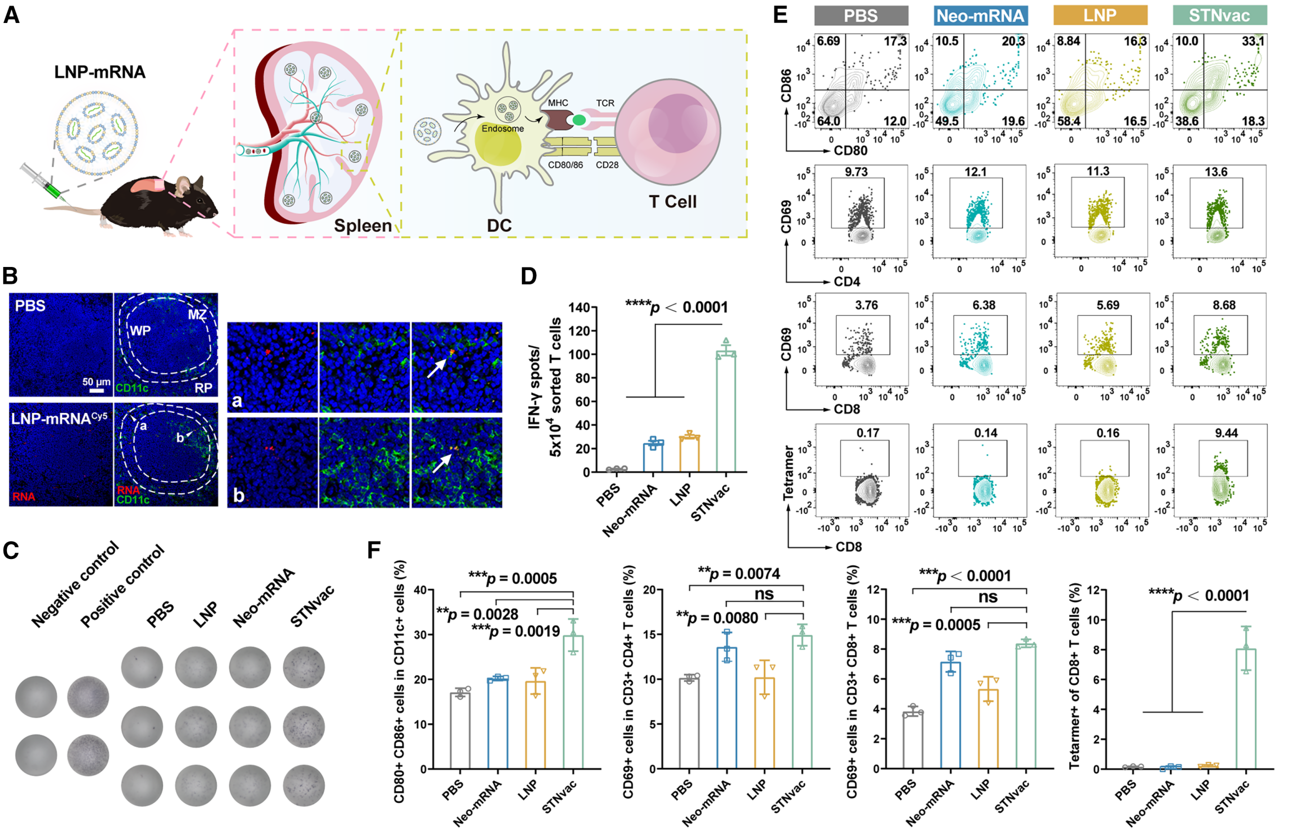

Figure 2. Splenic immune responses mediated by STNvac

(A) Schematic illustration showing that STNvac promotes DC activation and antigen-specific T cell induction.

(B) Representative immunofluorescence images showing the localization of CD11c^{+} DCs (green) and Cy5-labeled mRNA (red) in the spleen of C57BL/6 mouse 6 h after intravenous administration of LNP-mRNA^{Cy5} (N/P = 0.5). MZ, marginal zone; WP, white pulp; RP, red pulp. Scale bars, 50 μm.

(C and D) *Ex vivo* IFN-γ ELISpot analysis of STNvac-immunized mice. (C) Representative ELISpot images. (D) Quantification of IFN-γ spot-forming units.

(E and F) Flow cytometry analysis of splenic immune cell activation after 48 h of STNvac administration. (E) Representative contour plots. (F) Quantitative analysis. Statistics: one-way ANOVA for (D) and (F). Mean ± SD (*n* = 3 biological replicates). Significance levels: $^{**}p < 0.01$, $^{***}p < 0.001$, $^{****}p < 0.0001$. See also Figures S3 and S4.

and redistributed DCs toward the T cell zone (49.6%), suggesting enhanced antigen-presenting activity of splenic DCs.

We incorporated seven neoantigens previously identified by our group (*Ptpn2_I383T, Traf7_C403W, Mapk3_S284F, Lmf1_F523V, Lbr_A341P, Dtnb_K40T*, and *Samd91_K752M*)[16] into the mRNA coding region (Table S1) and formulated the STNvac using the optimized DOTMA/DOPE LNPs (N/P = 0.5). To evaluate immunogenicity, STNvac (10 μg mRNA per mouse) was administered intravenously, and splenic immune cells were isolated 48 h post-vaccination. *Ex vivo* interferon gamma (IFN-γ) ELISpot revealed that splenic T cells from STNvac-immunized mice secreted 38.7-fold higher IFN-γ than PBS control when co-cultured with mouse primary DCs pre-stimulated with the seven HCC neoantigen peptides (Figures 2C and 2D), confirming potent neoantigen-specific T cell activation. Flow cytometry further showed enhanced DC maturation (CD80^{+} CD86^{+}, 29.87% vs. 17.10%; 1.75-fold) and increased activation of splenic CD4^{+} and CD8^{+} T cells (CD69^{+}, 1.5- and 2.2-fold, respectively) after a single STNvac dose (Figures 2E, 2F, and S4). To track antigen-specific responses, we employed a fluorescent peptide-major histocompatibility complex (MHC) tetramer for the most immunogenic neoantigen *Ptpn2_I383T*[16] and observed robust expansion of Ptpn2-specific CD8^{+} T cells in STNvac-treated group (8.09%) compared with Neo-mRNA (0.11%) or LNP alone (0.23%). Collectively, these results suggest that STNvac effectively induces strong splenic antigen-specific immune responses.

STNvac shows strong efficacy in both the treatment and prevention of orthotopic HCC

To evaluate the therapeutic efficacy of STNvac for HCC, an orthotopic HCC tumor model was established using the Hepa1-6-Luc cells. Before therapeutic evaluation, we confirmed that LNP-mRNA maintained predominant spleen-preferential expression in orthotopic HCC-bearing mice (Figure S5). Since dosing regimen critically influences mRNA vaccine efficacy,[44,45] we compared single (1×), double (2×), triple (3×), and quadruple (4×) administrations, using a fixed total mRNA dose of 20 μg per mouse. Among these, the 3× group showed the most pronounced antitumor effect, achieving the highest

CellPress
OPEN ACCESS

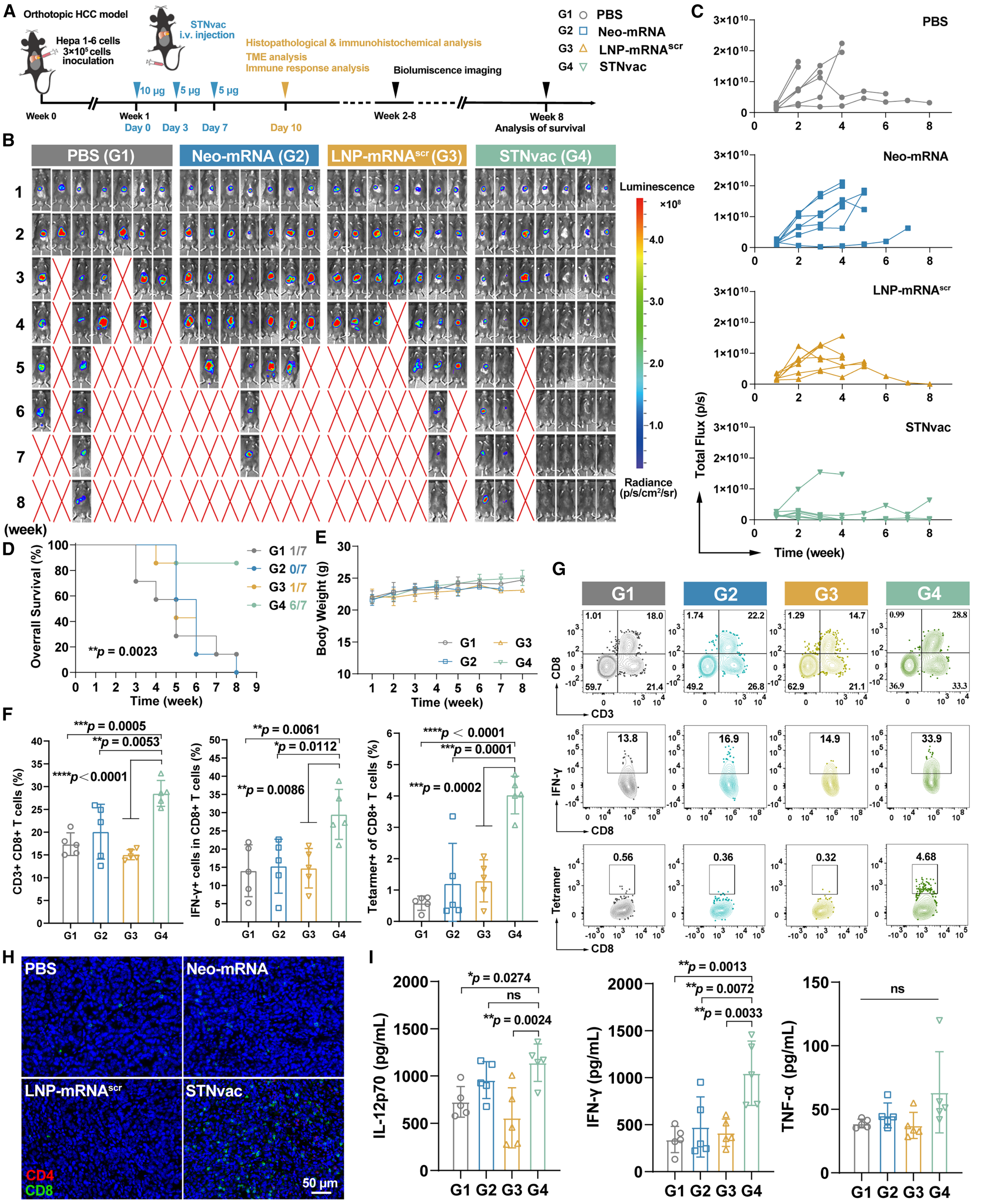

(legend on next page)

progression-free survival (PFS) and overall survival (OS) rates (Figure S6). Across all regimens, STNvac consistently outperformed the peptide-based neoantigen vaccine (Pep-NeoVac, adjuvanted with Poly I:C), exhibiting superior tumor control (Figure S6). The three-dose regimen achieves superior efficacy primarily due to the homologous prime-boost strategy.[46,47] Furthermore, in our study, the three-dose regimen outperformed the four-dose regimen, possibly because of the higher priming dosage and the more timely intervention during early tumor progression.

The therapeutic efficacy of STNvac (G4) was further confirmed using PBS (G1), pure Neo-mRNA (G2), and LNP-scramble mRNA (LNP-mRNA^{scr}, G3) as controls. The optimized three-dose vaccination regimen was adopted for subsequent studies, as illustrated in Figure 3A. As shown in Figures 3B and 3C, mice in G4 had higher response and PFS rates (85.71% and 71.43%, respectively) compared to G2 (14.28% and 0%) and G3 (14.28% and 14.28%). Four mice in G4 achieved complete tumor regression from week 4 to week 8. The survival rates across different treatments were 1/7, 0/7, 1/7, and 6/7, respectively (Figure 3D). Mice in G2 and G3 showed slightly extended survival, likely due to weak innate immune responses induced by mRNA. Histopathological analyses, including H&E, Ki67, and TUNEL staining (Figure S7A), revealed marked structural disruption, suppressed proliferation, and extensive apoptosis in STNvac-treated tumors, providing histological evidence for tumor regression. Together, these results highlight the potent therapeutic efficacy of STNvac in orthotopic HCC. In addition, the body weight of treated mice remained stable, and short-term serum biochemistry and major organ histology revealed no detectable abnormalities (Figures 3E, S7B, and S7C), indicating good acute tolerability of STNvac. To validate the advantage of spleen-targeted delivery, we compared intravenous STNvac with an intramuscular mRNA vaccine (IMNvac) formulated using the Food and Drug Administration (FDA)-approved SM-102-based LNP system from Moderna's mRNA-1273. STNvac achieved markedly stronger tumor suppression and higher survival (5/8 vs. 2/8; Figure S8), confirming the therapeutic benefit of systemic spleen-directed delivery. In splenectomized orthotopic HCC models, tumor progression was accelerated in both PBS- and STNvac-treated mice, and the efficacy of STNvac was markedly reduced, confirming that its antitumor activity relies on spleen-mediated immune responses (Figure S8). After confirming the therapeutic efficacy, we evaluated its biosafety in tumor-free mice. STNvac induced transient and self-limited innate activation, without sustained inflammation, biochemical abnormalities, or histopathological organ damage, supporting its systemic and long-term safety (Figure S9).

Having confirmed both efficacy and safety *in vivo*, we next explored whether STNvac could elicit durable immune protection capable of preventing tumor initiation. Given the increased splenic effector memory CD8$^+$ T cells (CD8$^+$ T$_{EM}$ cells, key mediators of rapid antitumor responses) observed in the therapeutic model (Figure S7D), we next examined whether this response could extend to long-term immune memory. In a prophylactic tumor challenge model, STNvac-immunized mice exhibited markedly reduced tumor incidence and overall burden, with half showing complete tumor rejection, demonstrating that STNvac can induce long-lasting immune memory capable of preventing HCC recurrence (Figure S10). Encouraged by these results, we further investigated the potential of STNvac to suppress liver metastasis. A liver metastasis model was established by intrahepatic implantation of Lewis lung carcinoma (LLC) cells, and an LLC-specific spleen-targeted mRNA vaccine (LLC-STNvac) was generated by incorporating previously identified LLC neoantigens[48,49] (Table S1) into the same LNP-mRNA formulation used for HCC-STNvac. LLC-STNvac partially inhibited tumor progression compared with PBS controls, although complete regression was not achieved owing to the high malignancy of LLC (Figure S11). These findings indicate that while STNvac exhibits therapeutic potential in highly aggressive liver metastatic models, further optimization will be needed to enhance its efficacy.

After validating the tumor-inhibitory effects across multiple tumor models, we analyzed the TME to assess its immunological effects. Given the key role of CD8$^+$ T cells in tumor-specific cytotoxicity, we examined intratumoral CD8$^+$ T cells 3 days after the final vaccination (day 10). Flow cytometry revealed that STNvac treatment (G4) induced the highest level of CD8$^+$ T cell infiltration (Figures 3F, 3G, andS12), which was corroborated by immunofluorescence staining (Figure 3H). Additionally, CD8$^+$ T cells in the STNvac group exhibited significantly higher IFN-γ expressions (29.52%) compared with the PBS group (14.03%), indicating enhanced effector functions. Ptpn2-MHC tetramer staining further confirmed the recruitment of neoantigen-specific CD8$^+$ T cells (4.03%) into tumor tissues. In parallel, proinflammatory cytokines including interleukin-12 (IL-12), IFN-γ, and tumor necrosis factor α (TNF-α) were all elevated in tumors following STNvac treatment (Figure 3I). Collectively, these findings demonstrate

Figure 3. Therapeutic efficacy and tumor microenvironment (TME) alterations induced by STNvac treatment

(A) Schematic illustration of the treatment schedule ($n = 7$ mice per group).

(B) Bioluminescence images showing tumor burden in orthotopic HCC-bearing mice receiving PBS (G1), Neo-mRNA (G2), LNP-scramble mRNA (G3), or STNvac (G4) during the 8-week observation period.

(C) Total bioluminescence flux for individual mice corresponding to (B).

(D) Survival curves of mice in different treatment groups.

(E) Mean body weight of mice monitored throughout the study period, showing no significant loss.

(F and G) Flow cytometry analysis of tumor-infiltrating immune cells collected 72 h after the final vaccination (day 10): (F) quantitative analysis ($n = 5$ biological replicates) and (G) representative contour plots.

(H) Immunofluorescence staining of CD4$^+$ and CD8$^+$ T cells in dissected tumor sections. Scale bars, 50 μm.

(I) Cytokine levels (IL-12, IFN-γ, and TNF-α) in tumor lysates measured by ELISA ($n = 5$ biological replicates, day 10).

Statistics: one-way ANOVA for (F) and (I); log rank (Mantel-Cox) test for (D). Mean ± SD. Significance levels: $*p < 0.05$; $**p < 0.01$; $***p < 0.001$; $****p < 0.0001$. See also Figures S5–S12 and S25.

that STNvac exerts potent therapeutic and prophylactic efficacy against HCC while alleviating the immunosuppressive state of the TME.

STNvac induces antigen-specific tumor cell killing

To elucidate the immune mechanisms underlying STNvac-mediated tumor regression, tumor samples from STNvac- and PBS-treated groups were analyzed by single-cell RNA sequencing and targeted sequencing for neoantigen-derived mutations. Nine major cell populations were identified, with immune subsets including T cells, DCs, B cells, and natural killer (NK) cells markedly enriched following STNvac treatment (Figure 4A). Tumor cells were distinguished from hepatocytes using SCEVAN (a CNV-based classifier), which revealed an approximate 35.6% reduction in tumor-cell abundance after STNvac treatment (Figure 4B), supporting its potent immune-mediated tumor clearance.

To confirm neoantigen-specific cytotoxicity, we examined tumor cells harboring targeted neoantigen-derived mutations at the single-cell level. Targeted sequencing revealed a marked reduction in mutation-bearing tumor cells following STNvac treatment (Figure 4C). Consistent with our previous findings,[16] tumor cells presenting highly immunogenic neoantigens such as *Ptpn2_I383* and *Traf7_C403W* were preferentially eliminated (Figure 4D). Moreover, tumor-cell clearance correlated with the number of neoantigens expressed per cell, with those carrying more than two neoantigens showing a greater reduction (Figures 4C and 4E), indicating that multi-neoantigen expression enhances susceptibility to T cell-mediated killing. Cell-cell communication analysis further revealed strengthened interactions between tumor and immune compartments after STNvac treatment (Figure 4F). Specifically, in the STNvac group, but not PBS group, two major classes of ligand-receptor interactions were observed: MHC I molecules on tumor cells engaging CD8 receptors on T cells (e.g., H2-k1-CD8a/b1), representing classical antigen presentation, and laminin-CD44 interactions (e.g., Lama5-CD44, Lamb2-CD44), which facilitate immune cell infiltration. These coordinated communication networks underscore the dual role of STNvac in promoting antigen recognition and effector-cell recruitment. Overall, these results suggest that STNvac induces robust antigen-specific tumor cell killing.

STNvac promotes ISG15$^+$ CD8$^+$ T cell activation and cytotoxicity

Encouraged by the enhanced antitumor immunity induced by STNvac, we next conducted single-cell RNA sequencing on CD45$^+$ immune cells isolated from tumor tissues to characterize changes of tumor immune microenvironment following STNvac treatment. Six major immune populations were identified, including T cells, B cells, NK cells, macrophages, DCs, and granulocytes (Figure 5A). Consistent with the single-cell transcriptomic analysis of tumor tissues (Figure 4A), STNvac significantly increased T cells, B cells, and NK cells, with T cells showing the most pronounced expansion (Figure 5B). To further examine T cell functional status, T cells were re-clustered into nine subsets, comprising one CD4$^+$ T cell cluster (cluster 0) and seven CD8$^+$ T cell clusters (clusters 1–7) (Figures 5C and S13). Among these, clusters 1, 4, and 5 expanded markedly following STNvac treatment (Figure 5D) and expressed high levels of effector molecules IFN-γ and GZMB, together with the activation and residency marker CD69 (Figure 5E). As shown in our previous study,[16] neoantigen-specific T cells are predominantly enriched in CD69$^+$ subsets, suggesting that these three clusters represent neoantigen-specific effector T cells. Notably, clusters 4 and 5 also expressed the proliferation marker Ki67 and the signaling adaptor protein FcεRI (enhancing TCR-mediated antigen recognition), implying that they represent early activated cytotoxic T cells. Interestingly, all three effector T cell clusters (1, 4, and 5) specifically expressed ISG15, a 17-kDa ubiquitin-like protein strongly induced by type I interferon (IFN-α/β).[50,51] Previous studies have shown that ISG15 enhances the cytotoxicity and cytokine secretion of CD8$^+$ T cells, thereby promoting tumor cell elimination.[50,52] Based on these findings, we proposed that ISG15$^+$ CD8$^+$ T cells play a key role in STNvac-mediated immunity. Consistent with this, multicolor immunofluorescence staining revealed that ISG15$^+$ CD8$^+$ T cells in STNvac-treated tumors co-expressed the cytotoxic molecules GZMB and IFN-γ, whereas such cells were scarcely detected in PBS controls (Figures 5F and 5G). These results support that ISG15$^+$ CD8$^+$ T cells represent an activated cytotoxic subset induced by STNvac. In addition, immunofluorescence staining in the prophylactic model confirmed the presence of ISG15$^+$ CD8$^+$ T cells within residual lesions (Figure S10D), suggesting that vaccine-induced cytotoxic T cells can be effectively recalled upon tumor challenge. To further explore their functional characteristics, Gene Ontology (GO) and Kyoto Encyclopedia of Genomes (KEGG) analyses were performed on differentially expressed genes in ISG15$^+$ CD8$^+$ T cells between PBS and STNvac groups. Both analyses revealed significant enrichment of antigen processing and presentation-associated pathways (e.g., MHC protein binding and antigen binding), suggesting preferential activation of ISG15$^+$ CD8$^+$ T cells via STNvac-induced antigen presentation (Figure 5H). Consistently, short-term *in vivo* blockade of MHC-I (H-2Kb) during vaccination markedly reduced the enrichment of activated ISG15$^+$ CD69$^+$ cells within tumor-infiltrating CD8$^+$ lymphocytes, supporting an MHC-I-dependent mechanism for STNvac-induced activation of this functional T cell subset (Figure S14). Moreover, analysis of TCGA liver cancer data showed that patients with higher ISG15$^+$ CD8$^+$ T cell signatures exhibited significantly improved 5-year OS (p = 0.0052) and progression-free interval (PFI, p = 0.0013) (Figure 5I). Collectively, these findings indicate that STNvac induces neoantigen-specific ISG15$^+$ CD8$^+$ T cells through enhanced antigen presentation by APCs, resulting in robust activation and potent cytotoxic activity that contribute crucially to STNvac-mediated tumor regression.

GZMA-F2R interaction between ISG15$^+$ CD8$^+$ T cells and APCs mediates the activation and cytotoxic function of ISG15$^+$ CD8$^+$ T cells

Building on the STNvac-induced enhancement of antigen presentation to ISG15$^+$ CD8$^+$ T cells, we further investigated their communications with potential APCs within the TME, including B cells, CD4$^+$ T cells, and DCs. Single-cell RNA sequencing of CD45$^+$ immune cells revealed a significant enhancement in antigen-presenting interactions between these APCs and ISG15$^+$

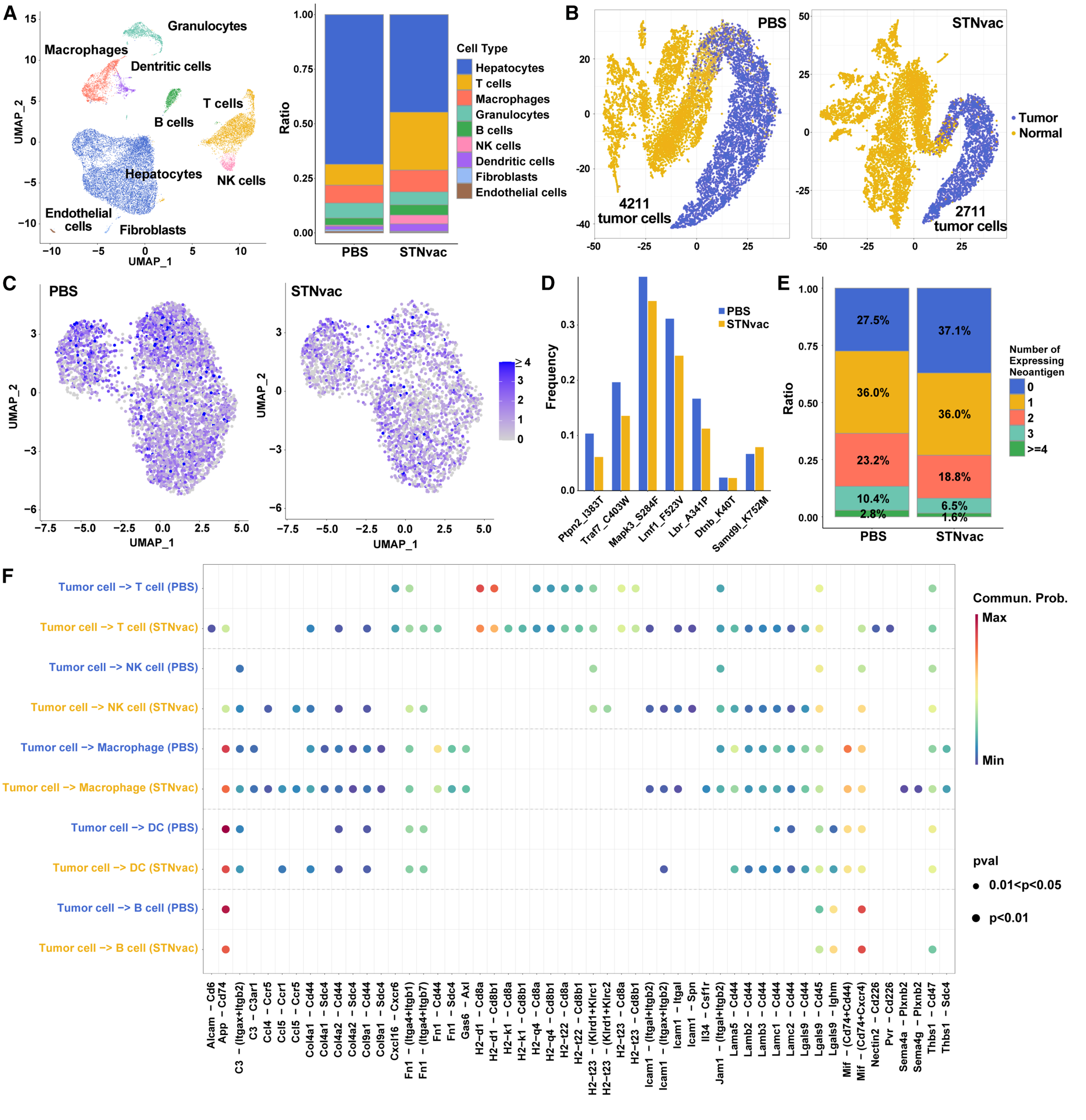

Figure 4. STNvac induces antigen-specific tumor killing

(A) UMAP visualization of single-cell transcriptomes from STNvac- and PBS-treated tumors showing nine major cell populations and a bar plot of their relative proportion.

(B) Identification of tumor cells using SCEVAN.

(C) Distribution changes of neoantigen expression after STNvac treatment.

(D) Changes in the proportion of tumor cells expressing specific neoantigens after STNvac treatment. Subclones carrying highly immunogenic neoantigens (*Ptpn2_I383T* and *Traf7_C403W*) were significantly eliminated, whereas weakly immunogenic ones (*Samd91_K752M*, *Dtnb_K40T*) showed minimal change.

(E) Bar plot showing the proportion changes in tumor cells expressing various numbers of neoantigens after STNvac treatment.

(F) Bubble plot of cell-cell interaction showing ligand-receptor pairs between tumor cells and immune cells (T cells, NK cells, macrophages, DCs, and B cells).

$CD8^+$ T cells (e.g., H2K1-CD8a/b and H2D1-CD8a/b) following STNvac treatment (Figure 6A). Interestingly, a distinct GZMA-F2R interaction was detected between APCs and $ISG15^+$ $CD8^+$ T cells post-STNvac treatment, albeit with relatively low interaction strength. F2R (protease-activated receptor 1 [PAR1]) has been reported to be expressed on $CD8^+$ T cells and to modulate

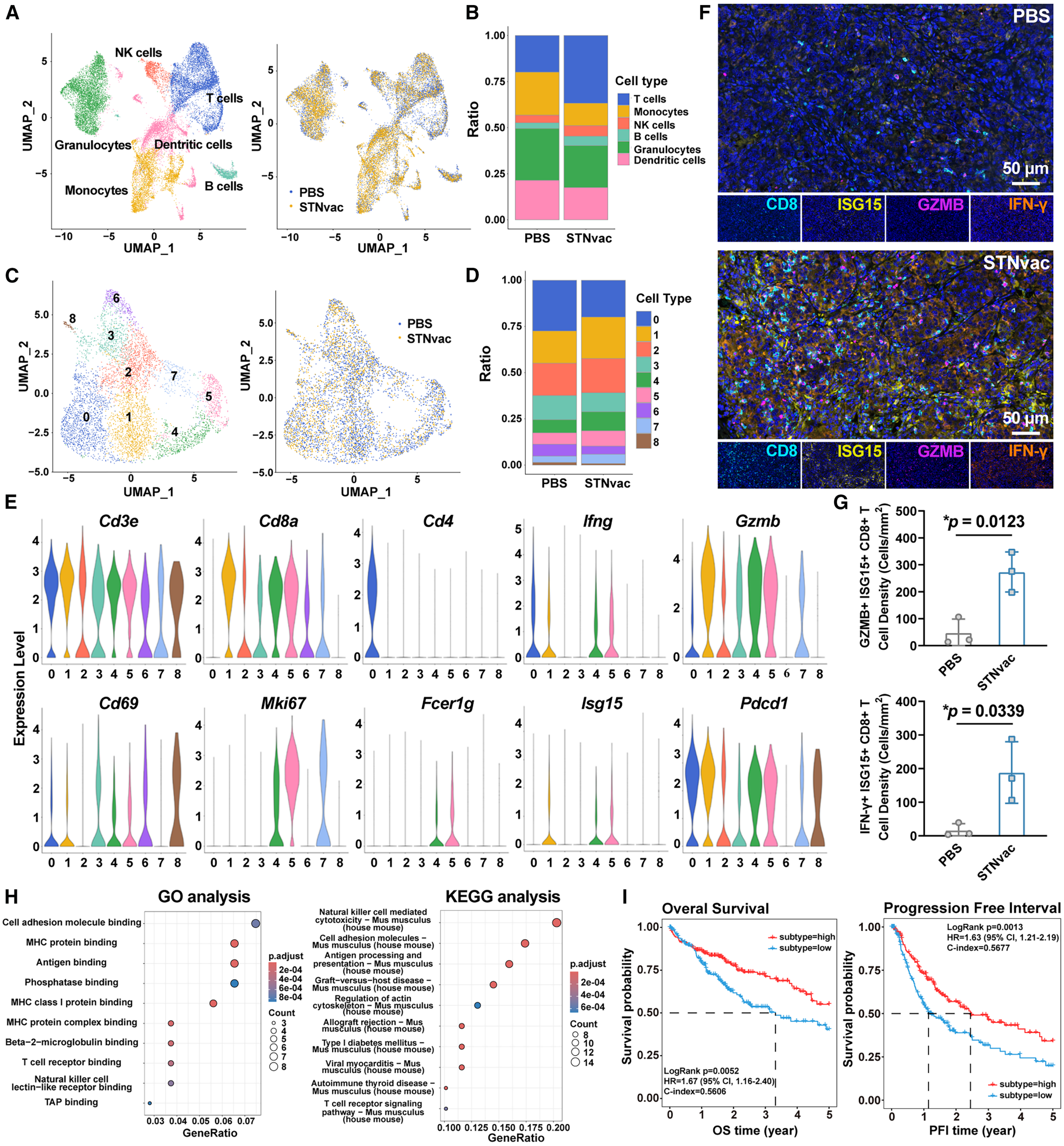

Figure 5. STNvac-induced enhancement of tumor infiltration, antigen-presenting capacity, and cytotoxic activity of ISG15^{+} CD8^{+} T cells

(A) UMAP visualization of single-cell transcriptomes from CD45^{+} immune cells.

(B) Relative proportions of major immune cell populations in PBS and STNvac groups.

(C) UMAP visualization of T cell clusters from PBS and STNvac groups.

(D) Relative proportions of different T cell clusters in PBS and STNvac groups.

(E) Violin plots showing expression levels of T cell function markers across T cell clusters.

(F) Representative multicolor immunofluorescence images of tumor sections showing co-localization of ISG15^{+} CD8^{+} T cells with GZMB and IFN-γ in PBS and STNvac groups. Scale bars, 50 μm.

(legend continued on next page)

their effector functions.[53] Emerging evidence suggests that the GZMA-F2R axis enhances T cell infiltration, activation, and cytotoxicity.[54] Based on these findings, we hypothesized that this interaction may contribute to the antitumor activity of ISG15$^+$ CD8$^+$ T cells during STNvac treatment. As expected, F2R expression was predominantly confined to ISG15$^+$ CD8$^+$ T cells, with minimal expression in other T cell subsets (Figure 6B). Meanwhile, GZMA expressions in B cells, CD4$^+$ T cells, and DCs were upregulated after STNvac treatment, supporting enhanced GZMA-F2R interaction between these APCs and ISG15$^+$ CD8$^+$ T cells.

To determine whether the GZMA-F2R interaction mediates the therapeutic efficacy of STNvac, we employed an F2R antagonist (F2RA, SCH79797) to block this pathway in orthotopic HCC mouse model. To confirm systemic exposure following intraperitoneal administration, pharmacokinetic and biodistribution profiles of F2RA were characterized (Figure S15), confirming adequate systemic availability for effective target inhibition. F2RA co-administration markedly attenuated the antitumor efficacy and survival benefits of STNvac (Figures 6C, 6D, and S16A). Multicolor immunofluorescence analysis further showed that F2RA intervention significantly reduced both the activation (CD69) and proliferation (Ki67) of intratumoral ISG15$^+$ CD8$^+$ T cells (Figures 6E and 6F), indicating that F2R is critically involved in their activation and expansion. Consistent results were obtained in an independent orthotopic intrahepatic cholangiocarcinoma (ICC) model established with KPC-OVA cells, in which F2RA similarly impaired the therapeutic efficacy of OVA-mRNA-loaded STNvac (Figure S17), further supporting the general relevance of the GZMA-F2R axis in mediating vaccine-induced antitumor immunity.

To assess whether the GZMA-F2R axis is conserved in humans, we cultured tumor-infiltrating lymphocytes (TILs) derived from tumor tissues of two postoperative HCC patients (HCCTIL01 and HCCTIL02) and evaluated CD8$^+$ TIL activation following GZMA stimulation and F2RA intervention (Figure 6G). Both CD3$^+$ CD8$^+$ TILs displayed near-uniform F2R expression (Figure S16B). For HCCTIL01, baseline 41BB expression (4.94%) increased to 9.16% following GZMA stimulation (0.05 ng/mL, 24 h) and decreased to 4.96% after F2RA pre-treatment (p = 0.0337) (Figures 6H and S16C–S16E). For HCCTIL02, which showed a higher baseline activation level (14.03% 41BB$^+$), GZMA stimulation caused no further increase, whereas F2RA reduced activation to 4.17% ($p < 0.001$). These results suggest that GZMA promotes early-stage CD8$^+$ T cell activation through an F2R-dependent mechanism. To determine whether this pathway also exists in primary human tumors, we re-analyzed single-cell RNA sequencing data from treatment-naive patients (GEO: GSE156625).[55] GZMA expression was detected in B cells (5.4%), DCs (6.8%), and CD4$^+$ T cells (33.7%), while F2R expression was 8.4% of ISG15$^+$ CD8$^+$ T cells. Cell-cell communication analysis further revealed GZMA-F2R interactions and antigen presentation axes (e.g., HLA-A-CD8A) between GZMA$^+$ APCs and F2R$^+$ ISG15$^+$ CD8$^+$ T cells (Figure S18), consistent with observations in the orthotopic HCC mouse model (Figure 6A). Collectively, these results support a conserved role for GZMA-F2R signaling in mediating CD8$^+$ T cell activation in both murine and human HCC.

STNvac induces the formation of TLS at the tumor invasive margin

Based on the finding that ISG15$^+$ CD8$^+$ T cells exhibited robust interactions with B cells, CD4$^+$ T cells, and DCs following STNvac treatment, we further examined their spatial organization within HCC tissues, as such architecture is closely associated with antitumor immunity.[36,37] Using probe-based spatial transcriptomics, we identified immune-cell-enriched clusters (4 and 7) that were markedly increased in the STNvac group (Figures 7A and 7B), consistent with single-cell analyses (Figures 4A and 5A). To enhance resolution, we applied TESLA to map ISG15$^+$ CD8$^+$ T cells (approximated by *Pclaf* and *Birc5*; Figure S19A) together with B cells, DCs, and CD4$^+$ T cells. These immune subsets showed pronounced colocalization in the STNvac group but were sparsely distributed in PBS control (Figures 7C and S19B). Because ISG15$^+$ CD8$^+$ T cells interact with APCs through the GZMA-F2R axis (Figure 6), we next examined whether GZMA-expressing APCs displayed a similar spatial pattern. Spatial transcriptomics revealed a marked increase of GZMA$^+$ APCs in the STNvac group that closely colocalized with ISG15$^+$ CD8$^+$ T cells (Figures 7C and S20A), indicating that the molecular interaction observed in single-cell data also occurs in spatial proximity *in vivo*. A comparable spatial colocalization between ISG15$^+$ CD8$^+$ T cells and GZMA$^+$ APCs was likewise detected in highly inflamed human HCC samples from Liu et al.'s study[56] (Figure S21).

Encouraged by the concurrent enrichment of CD8$^+$ T cells, B cells, DCs, and CD4$^+$ T cells, we next investigated whether STNvac promotes TLS formation in HCC. CXCL13, a key lymphoid chemokine driving B cell recruitment,[37,42,43] was markedly upregulated in the STNvac group (Figure S19C), and spatial transcriptomics revealed CXCL13 colocalization with ISG15$^+$ CD8$^+$ T cells, B cells, DCs, and CD4$^+$ T cells (Figure 7C). TLS-like aggregates with densely packed lymphocytes were evident at the tumor margins of STNvac-treated mouse (Figure 7D, white arrow), while smaller, loosely organized foci likely represented TLSs in formation (black arrows). These TLSs partially overlapped with ISG15$^+$ CD8$^+$ T cells, indicating an increased TLS density surrounded by neoantigen-specific T cells following STNvac treatment. To further verify TLS formation and organization, we performed multicolor immunofluorescence staining on serial tumor sections. CD20 was used to identify B cells and CD23 (expressed on follicular DCs and follicular B cells) to delineate germinal-center-like regions characteristic of secondary follicular TLSs,[37,39,57] while

(G) Quantification of ISG15$^+$ CD8$^+$ T cell density co-expressing GZMB and IFN-γ, corresponding to (F). Unpaired two-tailed t test; $^*p < 0.05$. Mean ± SD (n = 3 biological replicates).
(H) Comparative GO and KEGG pathway enrichment analyses of ISG15$^+$ CD8$^+$ T cells between PBS and STNvac groups.
(I) Kaplan-Meier curves showing 5-year overall survival (OS) and progression-free survival (PFI) for HCC patients in TCGA: LIHC cohort stratified by ISG15$^+$ CD8$^+$ T cell signatures. See also Figures S13 and S14.

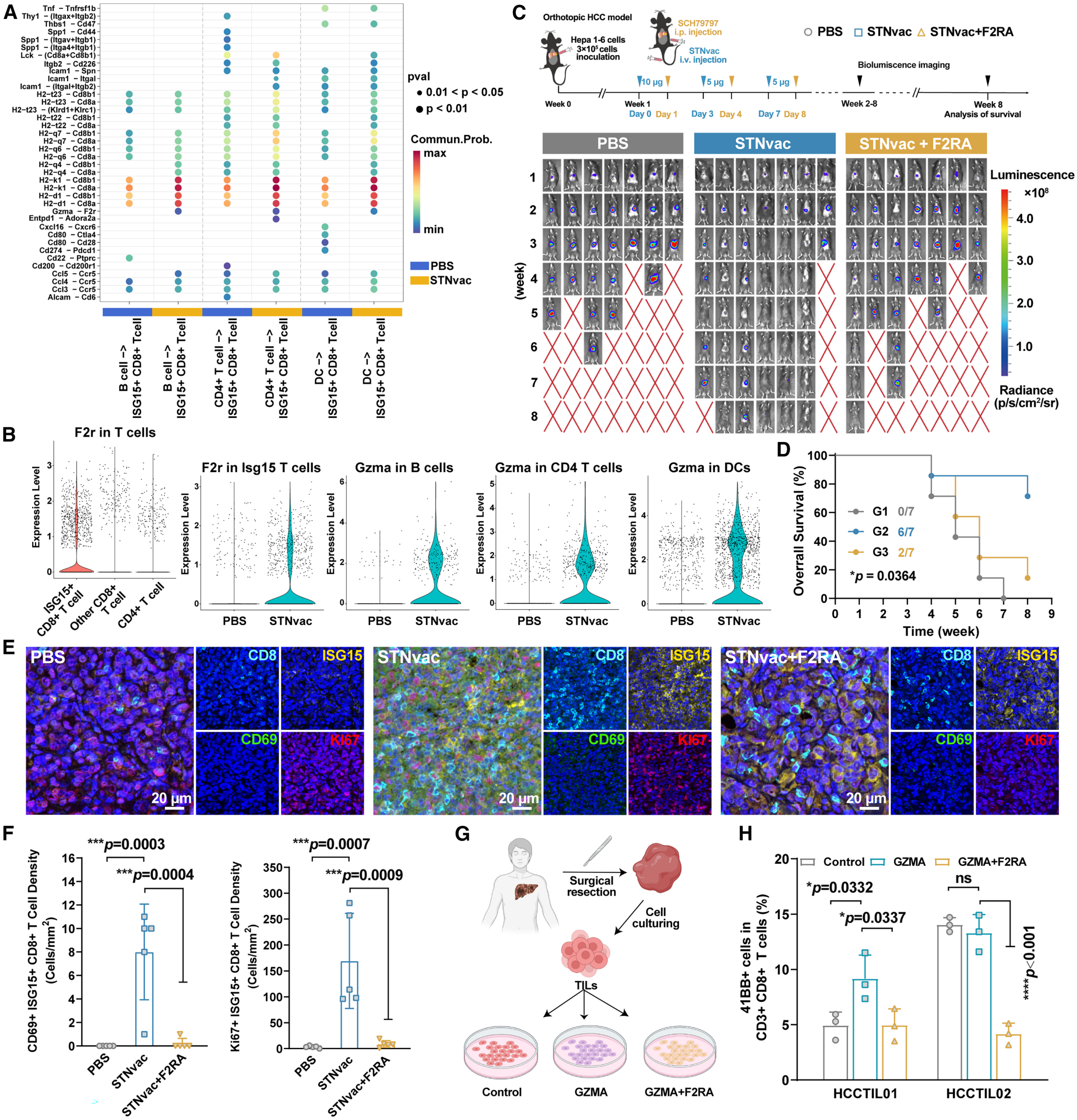

Figure 6. GZMA-F2R interaction mediates STNvac efficacy through T cell activation

(A) Bubble plot showing ligand-receptor pairs between ISG15^{+} CD8^{+} T cells and APCs (B cells, CD4^{+} T cells, and DCs).

(B) Violin plots showing F2R expression across T cell subsets and GZMA expression in APCs.

(C and D) Therapeutic evaluation of STNvac co-administration with F2R antagonist (F2RA, SCH79797) (n = 7 mice per group). (C) Bioluminescence images showing tumor burden in orthotopic HCC-bearing mice under the indicated treatments. (D) Survival curves of mice in different groups.

(E and F) Multicolor immunofluorescence staining of intratumoral Ki67^{+} CD69^{+} ISG15^{+} CD8^{+} T cells. (E) Representative images. Scale bars, 20 μm. (F) Quantitative analysis of positive cell density in five randomly selected areas per tumor section.

(G and H) Activation of human HCC TILs through the GZMA-F2R interaction. (G) Schematic illustration of the treatment schedule. (H) Quantitative analysis of 41BB^{+} CD3^{+} CD8^{+} T cells after 24 h of GZMA stimulation (n = 2 biological replicates, each analyzed in triplicate).

Statistics: one-way ANOVA for (F) and (H); log rank (Mantel-Cox) test for (D). Mean ± SD. Significance levels: *p < 0.05, ***p < 0.001, ****p < 0.0001. See also Figures S15–S19 and S25.

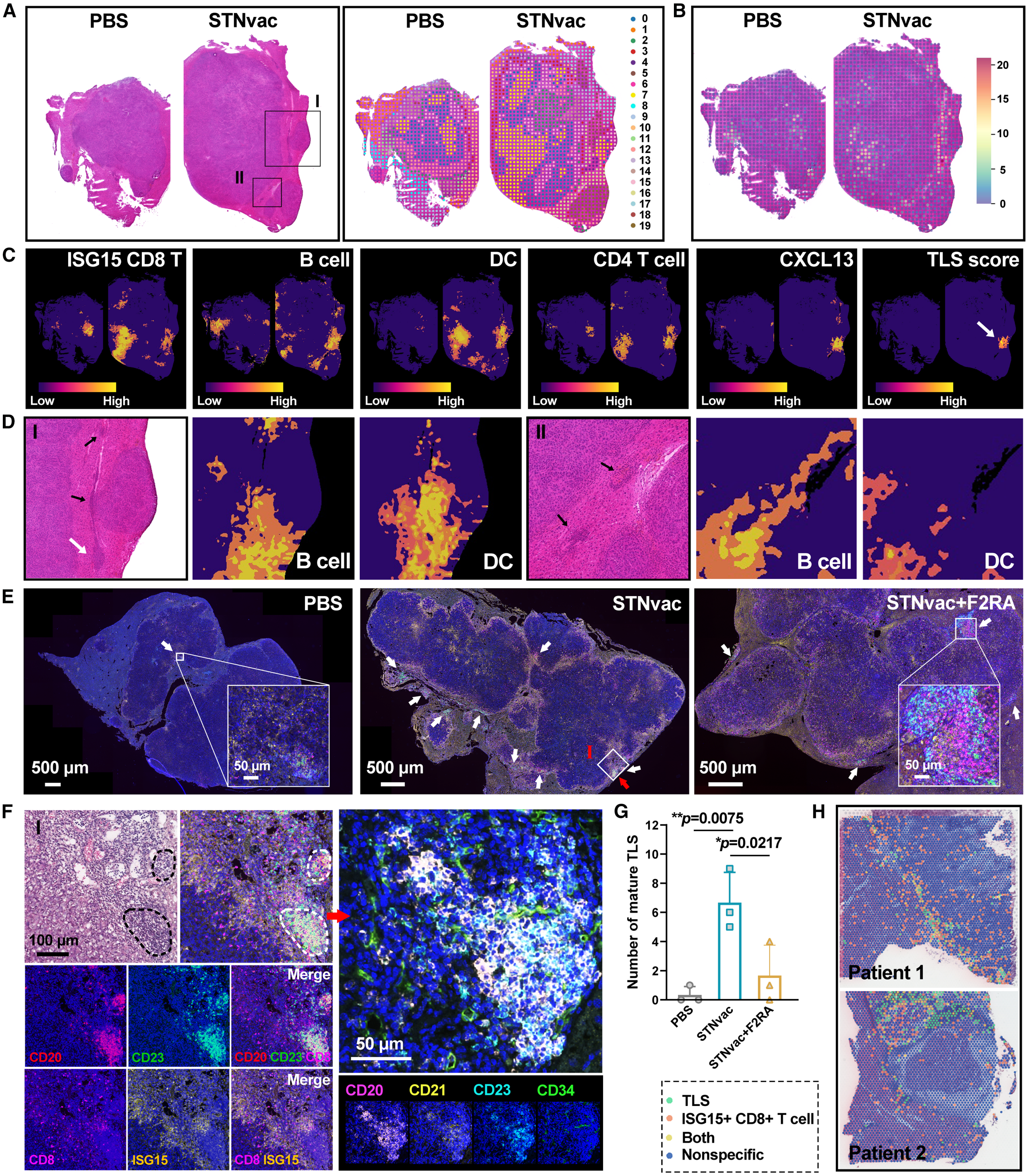

Figure 7. Spatial localization of ISG15⁺ CD8⁺ T cells and APCs and the formation of TLS following STNvac treatment

(A–D) Spatial transcriptomics analysis of tumor samples isolated from PBS- and STNvac-treated mice. (A) H&E staining and corresponding spatial clustering of transcriptome spots; 19 clusters were identified, with clusters 4 and 7 showing marked enrichment of immune cells (CD45⁺). (B) Spatial expression of *Ptprc* (CD45) mapped on H&E-stained tissues, highlighting immune-cell-dense regions. (C) Region of interest annotation based on TESLA, illustrating colocalization of ISG15⁺ CD8⁺ T cells (approximated by *Pclaf*/*Birc5*), B cells, DCs, and CD4⁺ T cells; CXCL13 expression overlapped with these immune clusters, and TLS-like regions were identified using a TLS score (co-localization of B cells, CD4⁺ T cells, DCs, and CXCL13). (D) Magnified views of regions I and II from (A), showing compact lymphoid aggregates (white arrows) and smaller, loosely organized foci (black arrows) at the tumor margin corresponding to TLS.

(legend continued on next page)

ISG15 and CD8 labeled neoantigen-specific cytotoxic T cells. Consistent with the spatial transcriptomics results, numerous CD20$^+$ CD23$^+$ TLSs were detected at the invasive margins of STNvac-treated tumors, including both compact and loosely organized aggregates (Figures 7E, 7F, and S22). ISG15$^+$ CD8$^+$ T cells were enriched around these TLSs and extended toward the tumor parenchyma, suggesting that neoantigen-specific T cells localized at TLS peripheries and infiltrated the tumor core. To further assess TLS structure and maturation, adjacent sections were stained with CD20, CD23, CD21, and CD34. CD21$^+$ follicular dendritic cells (FDCs) and CD34$^+$ perivascular structures reminiscent of high endothelial venules (HEV-like vessels) were observed at TLS boundaries in the STNvac group (Figures 7F and S23), confirming the presence of organized follicular and vascular elements. We next profiled intratumoral B cell differentiation using CD20, Bcl6, CD86, CD27, and CD138. TLSs from STNvac-treated tumors contained Bcl6$^+$ germinal-center-like B cells, CD86$^+$-activated B cells, CD27$^+$ memory-like B cells, and a small number of CD20$^-$ CD138$^+$ plasma cells, indicating active B cell activation and maturation within TLSs (Figure S23). Additionally, serum immunoglobulin G (IgG) levels increased progressively after STNvac administration, rising from day 10 and peaking at day 35 (Figure S9B), consistent with sustained B cell activation.

Given that STNvac induced mature TLSs enriched with activated APCs, we next examined whether the GZMA-F2R interaction identified at the single-cell level (Figures 6A and 6B) could also be visualized *in situ*. Four-color staining for GZMA, CD11c, CD19, and CD4 showed that GZMA$^+$ APCs, including CD19$^+$ B cells, CD11c$^+$ DCs, and CD4$^+$ T cells, were frequently localized within or immediately adjacent to TLS regions in the STNvac group but were scarce in PBS controls (Figure S20B). Quantification of GZMA$^+$ frequencies within each subset revealed increases across all three APC populations, with the most significant elevation observed in CD19$^+$ B cells and upward trends in CD11c$^+$ DCs and CD4$^+$ T cells. These findings are consistent with the proposed GZMA-F2R signaling between APCs and ISG15$^+$ CD8$^+$ T cells and support TLSs as spatial hubs that facilitate antigen presentation to neoantigen-specific T cells. Pharmacologic blockade of this pathway with the F2RA markedly impaired ISG15$^+$ CD8$^+$ T cell activation and attenuated the antitumor efficacy of STNvac (Figures 6C–6F). Consistently, F2RA intervention also significantly reduced TLS density at the tumor margins ($p = 0.0217$; Figures 7G and S24), supporting that the GZMA-F2R interaction contributes to TLS formation and maintenance. In human HCC spatial transcriptomic datasets from Liu et al's study,[56] ISG15$^+$ CD8$^+$ T cells were likewise enriched near or within TLS-like regions in the two immune-inflamed tumors (Figure 7H), suggesting that similar spatial organization occurs in human HCC. Together, these results demonstrate that STNvac induces TLS formation at tumor margins, sustaining antigen presentation and ISG15$^+$ CD8$^+$ T cell activation to drive durable antitumor immunity.

DISCUSSION

The limited abundance and restricted antigen-recognition capability of tumor-infiltrating T cells remain major barriers to effective immunotherapy for HCC. Personalized neoantigen vaccines represent a promising strategy to overcome these challenges by eliciting tumor-specific immune responses.[16] Building on our previous work, we developed a spleen-targeted mRNA vaccine (STNvac) to prime robust neoantigen-specific T cells and convert the immune “cold” TME into a responsive state. Several strategies have been explored to achieve spleen-selective mRNA expression, including tailoring structures of ionizable lipids,[58,59] modifying phospholipid tails,[32] incorporating additional SORT lipids,[29,31] and adjusting LNP/mRNA charge ratios.[27,30] Collectively, these studies suggest that LNP formulation parameters can influence particle size, surface charge, morphology, and the adsorbed protein corona,[60] thereby modulating spleen tropism. To simplify formulation and facilitate clinical translation, we employed a two-component DOTMA/DOPE LNP system,[27] which allows lipid pre-preparation and immediate mRNA complexation prior to administration. In our study, DOTMA/DOPE LNPs consistently exhibited predominant spleen-preferential mRNA expression across all N/P ratios (Figure 1G), suggesting that spleen tropism primarily derives from intrinsic formulation properties rather than surface charge or particle size alone. Through optimization, we identified the mildly negative formulation (N/P = 0.5) that achieved optimized splenic mRNA translation, enhanced DC activation, and robust priming of neoantigen-specific CD8$^+$ T cells. Employing this optimized formulation, STNvac effectively inhibited orthotopic HCC progression and even achieved complete tumor regression, with reproducible therapeutic outcomes across independent experimental cohorts (OS: PBS 3.7% vs. STNvac 77.8%; Figure S25).

While neoantigen vaccines have been shown to enhance antitumor immunity in HCC, the specific T cell subsets driving these responses remain poorly defined. Here, we identified a distinct ISG15$^+$ CD8$^+$ T cell subset that was markedly enriched in the HCC TME following STNvac. ISG15 is an interferon-stimulated ubiquitin-like modifier known to sustain CD8$^+$ T cell activation, persistence, and cytotoxic function.[52,61] Consistently, ISG15$^+$ CD8$^+$ T cells in STNvac-treated tumors displayed elevated antigen-processing and effector programs, together with upregulation of tissue residency and proliferation markers, indicating a highly activated neoantigen-specific phenotype. At the same time, this subset also expressed increased levels of exhaustion

(E–G) Multicolor immunofluorescence staining analysis of TLSs and ISG15$^+$ CD8$^+$ T cells in tumors from different groups. (E) Representative images showing CD20$^+$ B cells and CD23$^+$ follicular regions within TLSs, together with ISG15$^+$ CD8$^+$ T cells localized around TLS structures. Scale bars: 500 μm; inset, 50 μm. (F) Magnified views of Region I from (E), including H&E and additional multicolor staining for CD20, CD21, CD23, and CD34, highlighting organized FDC networks and HEV-like microvessels within TLS boundaries. Scale bars, 100 μm (left) and 50 μm (right). (G) Quantitative analysis of TLS density in tumors from different treatment groups (n = 3 biological replicates).
(H) Spatial transcriptomic mapping of human HCC tissues from Liu et al.'s study[56] showing ISG15$^+$ CD8$^+$ T cells located near or within TLS-like regions in immune-inflamed tumors. See also Figures S19–S24.

marker PD-1 (Figure 5E), indicating a functional state susceptible to checkpoint inhibition. Consistently, combination therapy with STNvac and anti-PD-1 antibody (STNvac+αPD-1) markedly improved tumor regression and survival compared with STNvac monotherapy (Figure S8), confirming the synergistic potential of PD-1 blockade to reinvigorate vaccine-induced ISG15$^+$ CD8$^+$ T cells.

Previous studies on neoantigen vaccines have mainly focused on the generation of neoantigen-specific T cells and their effects on tumor cells, leaving critical gaps in understanding their spatial organization and interactions with APCs in the TME. Notably, intratumoral antigen presentation and T cell re-education often occur within TLSs, which has been recognized as favorable prognostic indicators for cancer immunotherapy.[36,37,41] Using spatial transcriptomics and multiplex immunofluorescence, we observed that STNvac treatment promoted TLS formation, where neoantigen-specific ISG15$^+$ CD8$^+$ T cells clustered around TLSs and extended into the tumor parenchyma. Together with their enhanced antigen-processing and cytotoxic signatures, these findings suggest that ISG15$^+$ CD8$^+$ T cells undergo further activation through interactions with APCs in TLS niches. Furthermore, we identified the GZMA-F2R pair as a receptor-ligand axis mediating this interaction. While GZMA-F2R signaling has been implicated in T cell-tumor cell cytotoxicity,[54] our data reveal that GZMA expressed by APCs engages F2R on intratumoral CD8$^+$ T cells to promote their activation without affecting PD-1 expression (Figures S16C–S16E), indicating a role in early-stage T cell activation. Pharmacological blockade of this axis impaired TLS formation and attenuated the activation and proliferative state of intratumoral ISG15$^+$ CD8$^+$ T cells, supporting a role of APC-T cell communication via GZMA-F2R in TLS development or maintenance and local CD8$^+$ T cell activation or re-education. Although our data primarily support a local mechanism within the TME, the GZMA-F2R signaling may also influence earlier CD8$^+$ T cell activation in secondary lymphoid organs, thereby indirectly contributing to subsequent TLS formation within tumors. Collectively, these results highlight a mechanistic link between antigen-specific T cell-APC crosstalk and TLS formation in HCC and underscore the importance of spatial immune organization in vaccine-induced antitumor immunity.

Overall, this study demonstrated the feasibility and safety of the STNvac for precise immunotherapy against HCC. STNvac exhibited robust immunogenicity and potent antitumor efficacy, while promoting TLS formation that foster close interactions between vaccine-induced ISG15$^+$ CD8$^+$ T cells and APCs. This cellular crosstalk was partially mediated by the GZMA-F2R signaling axis, which we also observed in human HCC specimens, underscoring its clinical relevance as a targetable pathway. Together, these findings reveal a spatially organized immune mechanism underlying spleen-targeted mRNA vaccination and provide a rationale for advancing STNvac toward clinical evaluation in HCC patients.

Limitations of the study

Although STNvac elicited potent antitumor immunity and promoted TLS formation in murine HCC models, these findings require validation in clinical settings. The pharmacokinetics, spleen tropism, and safety of i.v. LNP delivery in humans remain unknown, and patient-specific neoantigen prediction and manufacturing may represent challenges for clinical translation. Mechanistically, our conclusions regarding vaccine-induced ISG15$^+$ CD8$^+$ T cells and their GZMA-F2R-mediated interactions with APCs are based on integrated spatial and transcriptional analyses rather than direct depletion experiments. The non-viability of ISG15$^+$ CD8$^+$ T cells after intracellular staining precluded their selective isolation or adoptive transfer, limiting definitive functional validation. Moreover, while STNvac promoted TLS formation and spatial coordination between ISG15$^+$ CD8$^+$ T cells and APCs, the molecular mechanisms driving TLS induction and the causal contribution of these T cells to TLS development remain to be clarified. Future studies should further delineate the molecular basis of TLS formation and confirm the functional relevance of ISG15$^+$ CD8$^+$ T cells in human HCC.

RESOURCE AVAILABILITY

Lead contact

Requests for further information and resources should be directed to and will be fulfilled by the lead contact, Xiaolong Liu (xiaoloong.liu@gmail.com).

Materials availability

Requests for sharing materials should be directed to the lead contact.

Data and code availability

- The single-cell RNA sequencing/target sequencing data and spatial transcriptome data in this study have been deposited in the Genome Sequencing Archive database of National Genomics Data Center (NGDC) under the accession number of NGDC: CRA022987 (https://ngdc.cncb.ac.cn/gsa/browse/CRA022987). This study also analyzed publicly available datasets, including GEO: GSE156625 (https://www.ncbi.nlm.nih.gov/geo/query/acc.cgi?acc=GSE156625), TCGA: LIHC (https://portal.gdc.cancer.gov/projects/TCGA-LIHC), and Mendeley Data: http://www.doi.org/10.17632/skrx2fz79n.1 (Liu's spatial transcriptome, https://data.mendeley.com/datasets/skrx2fz79n/1).
- This paper does not report any original code.
- Any additional information required to reanalyze the data reported in this work paper is available from the lead contact upon request.

ACKNOWLEDGMENTS

This work was supported by the National Natural Science Foundation of China (grant nos. 82403913 and U22A20328), the Natural Science Foundation of Fujian Province of China (grant nos. 2024J011229, 2024J010044, and 2023J06049), the Joint Funds for the innovation of science and Technology, Fujian province (grant no. 2024Y9419), the Major Research Projects for Young and Middle-aged Talent of Fujian Provincial Health Commission (grant no. 2022ZQNZD014), and the Scientific Research Foundation of State Key Laboratory of Vaccines for Infectious Diseases, Xiang An Biomedicine Laboratory (grant no. 2024XAKJ0101003). The authors want to thank Clinical Research Center for Infectious Diseases of Fujian Province (grant no. 2022Y2018) for technical support.

AUTHOR CONTRIBUTIONS

Conceptualization, X. Lin, Z.C., and X. Liu; methodology, X. Lin, G.C., R.T., M.W., D.Z., and Z.C.; investigation, X. Lin, G.C., R.T., M.W., D.Z., F.L., J.G., J.Y., X.D., X.Z., and L.Q.; data analysis, X. L in, G.C., F.L., J.G., J.Y., and Z.C.; writing original draft, X. Lin, G.C., and Z.C.; writing, review, and editing, H.Y. and X. Liu; funding, Z.C., H.Y., and X. Liu; supervision, Z.C. and X. Liu.

DECLARATION OF INTERESTS

The authors declare no competing interests.

STAR★METHODS

Detailed methods are provided in the online version of this paper and include the following:

- KEY RESOURCES TABLE
- EXPERIMENTAL MODEL AND STUDY PARTICIPANT DETAILS
  - Cell lines
  - Animals
  - Human HCC tumor-infiltrating lymphocytes (TILs)
- METHOD DETAILS
  - mRNA preparation
  - LNP-mRNA preparation and characterization
  - mRNA release and degradation analysis
  - *In vitro* experiments
  - *In vivo* and splenic immune-cell distribution of mRNA expression
  - *Ex vivo* IFN-γ ELISpot assay
  - Therapeutic efficacy in orthotopic HCC
  - Prophylactic and metastatic models
  - Mechanistic validation of the GZMA-F2R axis
  - Systemic safety evaluation
  - Pharmacokinetic and biodistribution analysis of F2RA
  - Flow cytometry of mouse spleen and tumor cells
  - Flow cytometry of human HCC TILs
  - Histology and immunostainings
  - Single cell data processing
  - Spatial transcriptome data analysis
  - Public dataset analysis
- QUANTIFICATION AND STATISTICAL ANALYSIS

SUPPLEMENTAL INFORMATION

Supplemental information can be found online at https://doi.org/10.1016/j.xcrm.2026.102754.

Received: May 6, 2025
Revised: November 4, 2025
Accepted: March 19, 2026
Published: April 20, 2026

REFERENCES

1. Foerster, F., Gairing, S.J., Ilyas, S.I., and Galle, P.R. (2022). Emerging immunotherapy for HCC: A guide for hepatologists. Hepatology *75*, 1604–1626. https://doi.org/10.1002/hep.32447.
2. Llovet, J.M., Castet, F., Heikenwalder, M., Maini, M.K., Mazzaferro, V., Pinato, D.J., Pikarsky, E., Zhu, A.X., and Finn, R.S. (2022). Immunotherapies for hepatocellular carcinoma. Nat. Rev. Clin. Oncol. *19*, 151–172. https://doi.org/10.1038/s41571-021-00573-2.
3. Sangro, B., Sarobe, P., Hervás-Stubbs, S., and Melero, I. (2021). Advances in immunotherapy for hepatocellular carcinoma. Nat. Rev. Gastroenterol. Hepatol. *18*, 525–543. https://doi.org/10.1038/s41575-021-00438-0.
4. Rimassa, L., Finn, R.S., and Sangro, B. (2023). Combination immunotherapy for hepatocellular carcinoma. J. Hepatol. *79*, 506–515. https://doi.org/10.1016/j.jhep.2023.03.003.
5. Kalbasi, A., and Ribas, A. (2020). Tumour-intrinsic resistance to immune checkpoint blockade. Nat. Rev. Immunol. *20*, 25–39. https://doi.org/10.1038/s41577-019-0218-4.
6. Yarchoan, M., Gane, E.J., Marron, T.U., Perales-Linares, R., Yan, J., Cooch, N., Shu, D.H., Fertig, E.J., Kagohara, L.T., Bartha, G., et al. (2024). Personalized neoantigen vaccine and pembrolizumab in advanced hepatocellular carcinoma: a phase 1/2 trial. Nat. Med. *30*, 1044–1053. https://doi.org/10.1038/s41591-024-02894-y.
7. Cheng, Y., Gunasegaran, B., Singh, H.D., Dutertre, C.A., Loh, C.Y., Lim, J.Q., Crawford, J.C., Lee, H.K., Zhang, X., Lee, B., et al. (2021). Non-terminally exhausted tumor-resident memory HBV-specific T cell responses correlate with relapse-free survival in hepatocellular carcinoma. Immunity *54*, 1825–1840.e7. https://doi.org/10.1016/j.immuni.2021.06.013.
8. Xie, N., Shen, G., Gao, W., Huang, Z., Huang, C., and Fu, L. (2023). Neoantigens: promising targets for cancer therapy. Signal Transduct. Target. Ther. *8*, 9. https://doi.org/10.1038/s41392-022-01270-x.
9. Blass, E., and Ott, P.A. (2021). Advances in the development of personalized neoantigen-based therapeutic cancer vaccines. Nat. Rev. Clin. Oncol. *18*, 215–229. https://doi.org/10.1038/s41571-020-00460-2.
10. Song, K., and Pun, S.H. (2024). Design and evaluation of synthetic delivery formulations for peptide-based cancer vaccines. BME Front. *5*, 0038. https://doi.org/10.34133/bmef.0038.
11. Hu, Z., Leet, D.E., Allesøe, R.L., Oliveira, G., Li, S., Luoma, A.M., Liu, J., Forman, J., Huang, T., Iorgulescu, J.B., et al. (2021). Personal neoantigen vaccines induce persistent memory T cell responses and epitope spreading in patients with melanoma. Nat. Med. *27*, 515–525. https://doi.org/10.1038/s41591-020-01206-4.
12. Keskin, D.B., Anandappa, A.J., Sun, J., Tirosh, I., Mathewson, N.D., Li, S., Oliveira, G., Giobbie-Hurder, A., Felt, K., Gjini, E., et al. (2019). Neoantigen vaccine generates intratumoral T cell responses in phase Ib glioblastoma trial. Nature *565*, 234–239. https://doi.org/10.1038/s41586-018-0792-9.
13. Ott, P.A., Hu-Lieskovan, S., Chmielowski, B., Govindan, R., Naing, A., Bhardwaj, N., Margolin, K., Awad, M.M., Hellmann, M.D., Lin, J.J., et al. (2020). A Phase Ib Trial of Personalized Neoantigen Therapy Plus Anti-PD-1 in Patients with Advanced Melanoma, Non-small Cell Lung Cancer, or Bladder Cancer. Cell *183*, 347–362.e24. https://doi.org/10.1016/j.cell.2020.08.053.
14. Peng, S., Chen, S., Hu, W., Mei, J., Zeng, X., Su, T., Wang, W., Chen, Z., Xiao, H., Zhou, Q., et al. (2022). Combination Neoantigen-Based Dendritic Cell Vaccination and Adoptive T-Cell Transfer Induces Antitumor Responses Against Recurrence of Hepatocellular Carcinoma. Cancer Immunol. Res. *10*, 728–744. https://doi.org/10.1158/2326-6066.CIR-21-0931.
15. Zhang, D., Lin, Z., Wu, M., Cai, Z., Zheng, Y., He, L., Li, Z., Zhou, J., Sun, L., Chen, G., et al. (2021). Cytosolic Delivery of Thiolated Neoantigen Nano-Vaccine Combined with Immune Checkpoint Blockade to Boost Anti-Cancer T Cell Immunity. Adv. Sci. *8*, 2003504. https://doi.org/10.1002/advs.202003504.
16. Chen, H., Li, Z., Qiu, L., Dong, X., Chen, G., Shi, Y., Cai, L., Liu, W., Ye, H., Zhou, Y., et al. (2022). Personalized neoantigen vaccine combined with PD-1 blockade increases CD8(+) tissue-resident memory T-cell infiltration in preclinical hepatocellular carcinoma models. J. Immunother. Cancer *10*, e004389. https://doi.org/10.1136/jitc-2021-004389.
17. Wang, Y., Zhao, Q., Zhao, B., Zheng, Y., Zhuang, Q., Liao, N., Wang, P., Cai, Z., Zhang, D., Zeng, Y., and Liu, X. (2022). Remodeling Tumor-Associated Neutrophils to Enhance Dendritic Cell-Based HCC Neoantigen Nano-Vaccine Efficiency. Adv. Sci. *9*, e2105631. https://doi.org/10.1002/advs.202105631.
18. Lin, Z., Jiang, C., Wang, P., Chen, Q., Wang, B., Fu, X., Liang, Y., Zhang, D., Zeng, Y., and Liu, X. (2023). Caveolin-mediated cytosolic delivery of spike nanoparticle enhances antitumor immunity of neoantigen vaccine for hepatocellular carcinoma. Theranostics *13*, 4166–4181. https://doi.org/10.7150/thno.85843.
19. Wu, M., Luo, Z., Cai, Z., Mao, Q., Li, Z., Li, H., Zhang, C., Zhang, Y., Zhong, A., Wu, L., and Liu, X. (2023). Spleen-targeted neoantigen DNA vaccine for personalized immunotherapy of hepatocellular carcinoma. EMBO Mol. Med. *15*, e16836. https://doi.org/10.15252/emmm.202216836.
20. Cai, Z., Su, X., Qiu, L., Li, Z., Li, X., Dong, X., Wei, F., Zhou, Y., Luo, L., Chen, G., et al. (2021). Personalized neoantigen vaccine prevents postoperative recurrence in hepatocellular carcinoma patients with vascular invasion. Mol. Cancer *20*, 164. https://doi.org/10.1186/s12943-021-01467-8.

21. Sayour, E.J., Boczkowski, D., Mitchell, D.A., and Nair, S.K. (2024). Cancer mRNA vaccines: clinical advances and future opportunities. Nat. Rev. Clin. Oncol. *21*, 489–500. https://doi.org/10.1038/s41571-024-00902-1.

22. Liu, C., Shi, Q., Huang, X., Koo, S., Kong, N., and Tao, W. (2023). mRNA-based cancer therapeutics. Nat. Rev. Cancer *23*, 526–543. https://doi.org/10.1038/s41568-023-00586-2.

23. Huang, X., Kong, N., Zhang, X., Cao, Y., Langer, R., and Tao, W. (2022). The landscape of mRNA nanomedicine. Nat. Med. *28*, 2273–2287. https://doi.org/10.1038/s41591-022-02061-1.

24. Chen, J., Ye, Z., Huang, C., Qiu, M., Song, D., Li, Y., and Xu, Q. (2022). Lipid nanoparticle-mediated lymph node-targeting delivery of mRNA cancer vaccine elicits robust CD8(+) T cell response. Proc. Natl. Acad. Sci. USA *119*, e2207841119. https://doi.org/10.1073/pnas.2207841119.

25. Hou, X., Zaks, T., Langer, R., and Dong, Y. (2021). Lipid nanoparticles for mRNA delivery. Nat. Rev. Mater. *6*, 1078–1094. https://doi.org/10.1038/s41578-021-00358-0.

26. Bevers, S., Kooijmans, S.A.A., Van de Velde, E., Evers, M.J.W., Seghers, S., Gitz-Francois, J.J.J.M., van Kronenburg, N.C.H., Fens, M.H.A.M., Mastrobattista, E., Hassler, L., et al. (2022). mRNA-LNP vaccines tuned for systemic immunization induce strong antitumor immunity by engaging splenic immune cells. Mol. Ther. *30*, 3078–3094. https://doi.org/10.1016/j.ymthe.2022.07.007.

27. Kranz, L.M., Diken, M., Haas, H., Kreiter, S., Loquai, C., Reuter, K.C., Meng, M., Fritz, D., Vascotto, F., Hefesha, H., et al. (2016). Systemic RNA delivery to dendritic cells exploits antiviral defence for cancer immunotherapy. Nature *534*, 396–401. https://doi.org/10.1038/nature18300.

28. Wang, F., Zhang, M., Tian, M., Lou, J., Pan, L., Gao, X., Zhang, L., Lou, X., Zhu, L., Sheng, Y., et al. (2023). Natural long-chain saturated fatty acids doped LNPs enabling spleen selective mRNA translation and potent cancer immunotherapy. Nano Res. *17*, 1804–1817. https://doi.org/10.1007/s12274-023-6111-2.

29. Pan, L., Zhang, L., Deng, W., Lou, J., Gao, X., Lou, X., Liu, Y., Yao, X., Sheng, Y., Yan, Y., et al. (2023). Spleen-selective co-delivery of mRNA and TLR4 agonist-loaded LNPs for synergistic immunostimulation and Th1 immune responses. J Control. Release *357*, 133–148. https://doi.org/10.1016/j.jconrel.2023.03.041.

30. LoPresti, S.T., Arral, M.L., Chaudhary, N., and Whitehead, K.A. (2022). The replacement of helper lipids with charged alternatives in lipid nanoparticles facilitates targeted mRNA delivery to the spleen and lungs. J. Control. Release *345*, 819–831. https://doi.org/10.1016/j.jconrel.2022.03.046.

31. Cheng, Q., Wei, T., Farbiak, L., Johnson, L.T., Dilliard, S.A., and Siegwart, D.J. (2020). Selective organ targeting (SORT) nanoparticles for tissue-specific mRNA delivery and CRISPR-Cas gene editing. Nat. Nanotechnol. *15*, 313–320. https://doi.org/10.1038/s41565-020-0669-6.

32. Liu, S., Cheng, Q., Wei, T., Yu, X., Johnson, L.T., Farbiak, L., and Siegwart, D.J. (2021). Membrane-destabilizing ionizable phospholipids for organ-selective mRNA delivery and CRISPR-Cas gene editing. Nat. Mater. *20*, 701–710. https://doi.org/10.1038/s41563-020-00886-0.

33. Luo, Z., Lin, Y., Meng, Y., Li, M., Ren, H., Shi, H., Cheng, Q., and Wei, T. (2024). Spleen-Targeted mRNA Vaccine Doped with Manganese Adjuvant for Robust Anticancer Immunity In Vivo. ACS Nano *18*, 30701–30715. https://doi.org/10.1021/acsnano.4c09902.

34. Chen, P., He, X., Hu, Y., Tian, X.L., Yu, X.Q., and Zhang, J. (2023). Spleen-Targeted mRNA Delivery by Amphiphilic Carbon Dots for Tumor Immunotherapy. ACS Appl. Mater. Interfaces *15*, 19937–19950. https://doi.org/10.1021/acsami.3c00494.

35. Sahin, U., Oehm, P., Derhovanessian, E., Jabulowsky, R.A., Vormehr, M., Gold, M., Maurus, D., Schwarck-Kokarakis, D., Kuhn, A.N., Omokoko, T., et al. (2020). An RNA vaccine drives immunity in checkpoint-inhibitor-treated melanoma. Nature *585*, 107–112. https://doi.org/10.1038/s41586-020-2537-9.

36. Kasikova, L., Rakova, J., Hensler, M., Lanickova, T., Tomankova, J., Pasulka, J., Drozenova, J., Mojzisova, K., Fialova, A., Vosahlikova, S., et al. (2024). Tertiary lymphoid structures and B cells determine clinically relevant T cell phenotypes in ovarian cancer. Nat. Commun. *15*, 2528. https://doi.org/10.1038/s41467-024-46873-w.

37. Schumacher, T.N., and Thommen, D.S. (2022). Tertiary lymphoid structures in cancer. Science *375*, eabf9419. https://doi.org/10.1126/science.abf9419.

38. Qin, M., Hamanishi, J., Ukita, M., Yamanoi, K., Takamatsu, S., Abiko, K., Murakami, R., Miyamoto, T., Suzuki, H., Ueda, A., et al. (2022). Tertiary lymphoid structures are associated with favorable survival outcomes in patients with endometrial cancer. Cancer Immunol. Immunother. *71*, 1431–1442. https://doi.org/10.1007/s00262-021-03093-1.

39. Siliņa, K., Soltermann, A., Attar, F.M., Casanova, R., Uckeley, Z.M., Thut, H., Wandres, M., Isajevs, S., Cheng, P., Curioni-Fontecedro, A., et al. (2018). Germinal Centers Determine the Prognostic Relevance of Tertiary Lymphoid Structures and Are Impaired by Corticosteroids in Lung Squamous Cell Carcinoma. Cancer Res. *78*, 1308–1320. https://doi.org/10.1158/0008-5472.CAN-17-1987.

40. Vanhersecke, L., Brunet, M., Guégan, J.P., Rey, C., Bougouin, A., Cousin, S., Moulec, S.L., Besse, B., Loriot, Y., Larroquette, M., et al. (2021). Mature tertiary lymphoid structures predict immune checkpoint inhibitor efficacy in solid tumors independently of PD-L1 expression. Nat. Cancer *2*, 794–802. https://doi.org/10.1038/s43018-021-00232-6.

41. Calderaro, J., Petitprez, F., Becht, E., Laurent, A., Hirsch, T.Z., Rousseau, B., Luciani, A., Amaddeo, G., Derman, J., Charpy, C., et al. (2019). Intra-tumoral tertiary lymphoid structures are associated with a low risk of early recurrence of hepatocellular carcinoma. J. Hepatol. *70*, 58–65. https://doi.org/10.1016/j.jhep.2018.09.003.

42. Peske, J.D., Thompson, E.D., Gemta, L., Baylis, R.A., Fu, Y.X., and Engelhard, V.H. (2015). Effector lymphocyte-induced lymph node-like vasculature enables naive T-cell entry into tumours and enhanced anti-tumour immunity. Nat. Commun. *6*, 7114. https://doi.org/10.1038/ncomms8114.

43. Workel, H.H., Lubbers, J.M., Arnold, R., Prins, T.M., van der Vlies, P., de Lange, K., Bosse, T., van Gool, I.C., Eggink, F.A., Wouters, M.C.A., et al. (2019). A Transcriptionally Distinct CXCL13(+)CD103(+)CD8(+) T-cell Population Is Associated with B-cell Recruitment and Neoantigen Load in Human Cancer. Cancer Immunol. Res. *7*, 784–796. https://doi.org/10.1158/2326-6066.CIR-18-0517.

44. Barbier, A.J., Jiang, A.Y., Zhang, P., Wooster, R., and Anderson, D.G. (2022). The clinical progress of mRNA vaccines and immunotherapies. Nat. Biotechnol. *40*, 840–854. https://doi.org/10.1038/s41587-022-01294-2.

45. Shroff, R.T., Chalasani, P., Wei, R., Pennington, D., Quirk, G., Schoenle, M.V., Peyton, K.L., Uhrlaub, J.L., Ripperger, T.J., Jergović, M., et al. (2021). Immune responses to two and three doses of the BNT162b2 mRNA vaccine in adults with solid tumors. Nat. Med. *27*, 2002–2011. https://doi.org/10.1038/s41591-021-01542-z.

46. Palgen, J.L., Feraoun, Y., Dzangué-Tchoupou, G., Joly, C., Martinon, F., Le Grand, R., and Beignon, A.S. (2021). Optimize Prime/Boost Vaccine Strategies: Trained Immunity as a New Player in the Game. Front. Immunol. *12*, 612747. https://doi.org/10.3389/fimmu.2021.612747.

47. Cheng, H., Peng, Z., Si, S., Alifu, X., Zhou, H., Chi, P., Zhuang, Y., Mo, M., and Yu, Y. (2022). Immunogenicity and Safety of Homologous and Heterologous Prime-Boost Immunization with COVID-19 Vaccine: Systematic Review and Meta-Analysis. Vaccines *10*, 798. https://doi.org/10.3390/vaccines10050798.

48. Lin, X., Tang, S., Guo, Y., Tang, R., Li, Z., Pan, X., Chen, G., Qiu, L., Dong, X., Zhang, L., et al. (2024). Personalized neoantigen vaccine enhances the therapeutic efficacy of bevacizumab and anti-PD-1 antibody in advanced non-small cell lung cancer. Cancer Immunol. Immunother. *73*, 26. https://doi.org/10.1007/s00262-023-03598-x.

49. Tang, S., Tang, R., Chen, G., Zhang, D., Lin, K., Yang, H., Fu, J., Guo, Y., Lin, F., Dong, X., et al. (2024). Personalized neoantigen hydrogel vaccine

CellPress
OPEN ACCESS

combined with PD-1 and CTLA-4 double blockade elicits antitumor response in liver metastases by activating intratumoral CD8(+)CD69(+) T cells. J. Immunother. Cancer *12*, e009543. https://doi.org/10.1136/jitc-2024-009543.

50. Fan, J.-B., Miyauchi-Ishida, S., Arimoto, K.-i., Liu, D., Yan, M., Liu, C.-W., Győrffy, B., and Zhang, D.-E. (2015). Type I IFN induces protein ISGylation to enhance cytokine expression and augments colonic inflammation. Proc. Natl. Acad. Sci. USA *112*, 14313–14318. https://doi.org/10.1073/pnas.1505690112.

51. Zhang, X., Bogunovic, D., Payelle-Brogard, B., Francois-Newton, V., Speer, S.D., Yuan, C., Volpi, S., Li, Z., Sanal, O., Mansouri, D., et al. (2015). Human intracellular ISG15 prevents interferon-α/β over-amplification and auto-inflammation. Nature *517*, 89–93. https://doi.org/10.1038/nature13801.

52. Villarreal, D.O., Wise, M.C., Siefert, R.J., Yan, J., Wood, L.M., and Weiner, D.B. (2015). Ubiquitin-like molecule ISG15 acts as an immune adjuvant to enhance antigen-specific CD8 T-cell tumor immunity. Mol. Ther. *23*, 1653–1662. https://doi.org/10.1038/mt.2015.120.

53. Chen, H., Smith, M., Herz, J., Li, T., Hasley, R., Le Saout, C., Zhu, Z., Cheng, J., Gronda, A., Martina, J.A., et al. (2021). The role of protease-activated receptor 1 signaling in CD8 T cell effector functions. iScience *24*, 103387. https://doi.org/10.1016/j.isci.2021.103387.

54. Gao, Y., Xu, Q., Li, X., Guo, Y., Zhang, B., Jin, Y., Zhu, C., Shen, Y., Yang, P., Shi, Y., et al. (2022). Heterogeneity induced GZMA-F2R communication inefficient impairs antitumor immunotherapy of PD-1 mAb through JAK2/STAT1 signal suppression in hepatocellular carcinoma. Cell Death Dis. *13*, 213. https://doi.org/10.1038/s41419-022-04654-7.

55. Sharma, A., Seow, J.J.W., Dutertre, C.A., Pai, R., Blériot, C., Mishra, A., Wong, R.M.M., Singh, G.S.N., Sudhagar, S., Khalilnezhad, S., et al. (2020). Onco-fetal Reprogramming of Endothelial Cells Drives Immunosuppressive Macrophages in Hepatocellular Carcinoma. Cell *183*, 377–394.e21. https://doi.org/10.1016/j.cell.2020.08.040.

56. Liu, Y., Xun, Z., Ma, K., Liang, S., Li, X., Zhou, S., Sun, L., Liu, Y., Du, Y., Guo, X., et al. (2023). Identification of a tumour immune barrier in the HCC microenvironment that determines the efficacy of immunotherapy. J. Hepatol. *78*, 770–782. https://doi.org/10.1016/j.jhep.2023.01.011.

57. Meylan, M., Petitprez, F., Becht, E., Bougoüin, A., Pupier, G., Calvez, A., Giglioli, I., Verkarre, V., Lacroix, G., Verneau, J., et al. (2022). Tertiary lymphoid structures generate and propagate anti-tumor antibody-producing plasma cells in renal cell cancer. Immunity *55*, 527–541.e5. https://doi.org/10.1016/j.immuni.2022.02.001.

58. Zhao, X., Chen, J., Qiu, M., Li, Y., Glass, Z., and Xu, Q. (2020). Imidazole-Based Synthetic Lipidoids for In Vivo mRNA Delivery into Primary T Lymphocytes. Angew. Chem. Int. Ed. Engl. *59*, 20083–20089. https://doi.org/10.1002/anie.202008082.

59. Qiu, M., Tang, Y., Chen, J., Muriph, R., Ye, Z., Huang, C., Evans, J., Henske, E.P., and Xu, Q. (2022). Lung-selective mRNA delivery of synthetic lipid nanoparticles for the treatment of pulmonary lymphangioleiomyomatosis. Proc. Natl. Acad. Sci. USA *119*, e2116271119. https://doi.org/10.1073/pnas.2116271119.

60. Chan, W.C.W. (2023). Principles of nanoparticle delivery to solid tumors. BME Front. *4*, 0016. https://doi.org/10.34133/bmef.0016.

61. Yeung, T.-L., Tsai, C.C., Leung, C.S., Au Yeung, C.-L., Thompson, M.S., Lu, K.H., Freedman, R.S., Birrer, M.J., Wong, K.-K., and Mok, S.C. (2018). ISG15 promotes ERK1 ISGylation, CD8+ T cell activation and suppresses ovarian cancer progression. Cancers *10*, 464. https://doi.org/10.3390/cancers10120464.

62. Zacharakis, N., Chinnasamy, H., Black, M., Xu, H., Lu, Y.C., Zheng, Z., Pasetto, A., Langhan, M., Shelton, T., Prickett, T., et al. (2018). Immune recognition of somatic mutations leading to complete durable regression in metastatic breast cancer. Nat. Med. *24*, 724–730. https://doi.org/10.1038/s41591-018-0040-8.

63. Fujimoto, D., and Kobayashi, H. (2024). PAR1 Is a Candidate Target for the Treatment of Peritoneal Dissemination in Gastric Cancer. Anticancer Res. *44*, 4857–4867. https://doi.org/10.21873/anticanres.17311.

64. Strande, J.L., Hsu, A., Su, J., Fu, X., Gross, G.J., and Baker, J.E. (2007). SCH 79797, a selective PAR1 antagonist, limits myocardial ischemia/reperfusion injury in rat hearts. Basic Res. Cardiol. *102*, 350–358. https://doi.org/10.1007/s00395-007-0653-4.

65. Yokono, Y., Hanada, K., Narita, M., Tatara, Y., Kawamura, Y., Miura, N., Kitayama, K., Nakata, M., Nozaka, M., Kato, T., et al. (2020). Blockade of PAR-1 Signaling Attenuates Cardiac Hypertrophy and Fibrosis in Renin-Overexpressing Hypertensive Mice. J. Am. Heart Assoc. *9*, e015616. https://doi.org/10.1161/JAHA.119.015616.

STAR★METHODS

KEY RESOURCES TABLE

REAGENT or RESOURCE	SOURCE	IDENTIFIER
Antibodies		
CD11c (Alexa Fluor™ 488, mouse)	eBioscience™	Cat#53-0114-82; RRID: AB_469903
CD11c (APC, mouse)	eBioscience™	Cat#17-0114-82; RRID: AB_469346
CD11b (APC, mouse)	eBioscience™	Cat#17-0112-82; RRID: AB_469343
CD19 (APC, mouse)	eBioscience™	Cat#17-0193-82; RRID: AB_1659676
CD3e (APC, mouse)	eBioscience™	Cat#17-0031-82; RRID: AB_469315
NK1.1 (APC, mouse)	eBioscience™	Cat#17-5941-82; RRID: AB_469479
CD80 (PE, mouse)	eBioscience™	Cat#12-0801-82; RRID: AB_465752
CD86 (FITC, mouse)	eBioscience™	Cat#11-0860-81; RRID: AB_465144
CD4 (PerCP, mouse)	BioLegend	Cat#100432; RRID: AB_893323
CD8a (PE, mouse)	BioLegend	Cat#100708; RRID: AB_312747
CD8a (FITC, mouse)	eBioscience™	Cat#11-0081-82; RRID: AB_464915
CD8a (PE, mouse)	eBioscience™	Cat#12-0081-82; RRID: AB_465530
CD69 (FITC, mouse)	BioLegend	Cat#104506; RRID: AB_313109
CD69 (PE-Cyanine7, mouse)	BioLegend	Cat#25-0691-82; RRID: AB_469637
CD45 (FITC, mouse)	eBioscience™	Cat#11-0451-85; RRID: AB_465051
CD44 (PE-Cyanine7, mouse)	eBioscience™	Cat#25-0441-82; RRID: AB_469623
CD62L (PerCP-Cyanine5.5, mouse)	eBioscience™	Cat#45-0621-82; RRID: AB_996667
IFN-γ (PE-Cyanine7, mouse)	eBioscience™	Cat#25-7311-82; RRID: AB_469680
ISG15 (Unconjugated, mouse)	Abcam	Cat#ab315281; RRID: N/A
Anti-rabbit IgG (H + L), F(ab')2 Fragment (Alexa Fluor® 647 Conjugate)	CST	Cat#4414S; RRID: N/A
Firefly luciferase (PE, intracellular)	Abcam	Cat#ab237253; RRID: N/A
PAR1(F2R) mAb (ATAP2, human TILs)	Invitrogen	Cat#35–2200; RRID: AB_2533198
Anti-mouse IgG (H + L), fluorescent secondary (CoraLite647)	Proteintech	Cat#SA00014-10; RRID: AB_2935615
Anti-Mouse MHC Class I (H-2Kb) Antibody (Y-3)	MCE	Cat#HY-P99135; RRID: N/A
InVivoMAb rat IgG2b isotype control	BioXCell	Cat#BE0090; RRID: N/A
CD3 (FITC, human TILs)	eBioscience™	Cat#11-0037-42; RRID: AB_2016669
CD8a (PE, human TILs)	eBioscience™	Cat#12-0089-42; RRID: AB_10804039
41BB (PerCP-eFluor™ 710, human TILs)	eBioscience™	Cat#46-1379-42; RRID: AB_2573716
PD-1 (APC, human TILs)	eBioscience™	Cat#17-9969-42; RRID: AB_10718533
αPD-1 (rat, mAb)	Leinco	Cat# Clone RMP1-14; RRID: AB_2749820
Ki67 (IHC, rabbit pAb)	Servicebio	Cat#GB111141; RRID:AB_3096315
CD4 (IF, rabbit pAb)	Servicebio	Cat#GB11064; RRID:AB_2904187
CD8 (IF, rabbit pAb)	Servicebio	Cat#GB114196; RRID:AB_3064847
CD8 (TSA-mIF, rabbit mAb)	CST	Cat#98941; RRID:AB_2756376
ISG15 (TSA-mIF, rabbit pAb)	Invitrogen	Cat#PA5-79523; RRID: AB_2746639
CD69 (TSA-mIF, rabbit pAb)	Proteintech	Cat#10803-1-AP; RRID: AB_2074965
Ki67 (TSA-mIF, rabbit pAb)	Abcam	Cat#ab15580; RRID: AB_443209
GZMB (TSA-mIF, rabbit mAb)	AiFang Biological	Cat#AFRM0352; RRID: N/A
IFN-γ (TSA-mIF, rabbit pAb)	GeneTex	Cat#GTX66714; RRID: N/A
GZMA (TSA-mIF, rabbit pAb)	Proteintech	Cat#11288-1-AP; RRID: AB_2114392
CD11c (TSA-mIF, rabbit mAb)	AiFang Biological	Cat#AFRM0294; RRID: N/A

(*Continued on next page*)

Continued

REAGENT or RESOURCE	SOURCE	IDENTIFIER
CD4 (TSA-mIF, rabbit mAb)	AiFang Biological	Cat#AFRM0003; RRID: N/A
CD19 (TSA-mIF, rabbit mAb)	AiFang Biological	Cat#AFRM0354; RRID: N/A
CD20 (TSA-mIF, rabbit mAb)	Abcam	Cat#ab64088; RRID:AB_1139386
CD23 (TSA-mIF, rabbit mAb)	Abcam	Cat#ab315289; RRID: N/A
CD21 (TSA-mIF, rabbit mAb)	Abcam	Cat#ab227662; RRID: N/A
CD34 (TSA-mIF, rabbit mAb)	AiFang Biological	Cat#AFRM0080; RRID: N/A
Bcl6 (TSA-mIF, rabbit pAb)	Invitrogen	Cat#PA5-27390; RRID:AB_2544866
CD138 (TSA-mIF, rabbit pAb)	Proteintech	Cat#10593-1-AP; RRID:AB_2182736
CD27 (TSA-mIF, rabbit mAb)	Abcam	Cat#ab214043; RRID: N/A
CD86 (TSA-mIF, rabbit mAb)	AiFang Biological	Cat#AFRM0167; RRID: N/A
Chemicals, peptides, and recombinant proteins		
DOTMA	Sinopeg	Cat#06040009700
DOPE	Sinopeg	Cat#06030000500
SM-102	Sinopeg	Cat#06040008800
DSPC	Sinopeg	Cat#06030001100
cholesterol	Sinopeg	Cat#06040015200
DMG-PEG2000	AVT	Cat#O02005
HindIII restriction enzyme	Thermo-Fisher	Cat#FD0505
Luciferin substrate	PerkinElmer	Cat#122799
Heparin sodium	Achem-block	Cat#ABC-B132998
SCH79797 (F2R antagonist, F2RA)	MCE	Cat#HY-14994
Recombinant GZMA	MCE	Cat#HY-P76377
OKT3	T&L biotechnology	Cat#GMP-TL101-0500
Trypan Blue Solution	Gibco	Cat#15250-061
Critical commercial assays		
Mouse IFN-γ ELISpot Kit	Mabtech	Cat#3321-4AST-2
H&E Stain Kit	Solarbio	Cat#G1120
One Step TUNEL Apoptosis Assay Kit	Beyotime	Cat#C1086
Mouse IL-12 ELISA kit	Boster Bio	Cat#EK0422
Mouse IFN-γ ELISA kit	Boster Bio	Cat# EK0375
Mouse IgG ELISA kit	Boster Bio	Cat#EK0101
Mouse IL-1β ELISA kit	Boster Bio	Cat#EK0394
Mouse IL-6 ELISA kit	Boster Bio	Cat#EK0411
Mouse TNF-α ELISA kit	Boster Bio	Cat#EK0527
Mouse IFN-β ELISA kit	FANKEW	Cat#F2124-A
Mouse C3a ELISA kit	Cusabio Biotech	Cat#CSB-E08666r
Mouse C5a ELISA kit	Cusabio Biotech	Cat#CSB-E14363r
Fixation/Permeabilization Solution Kit	BD Bioscience	Cat#554715
HyperScribe^{TM} All in One mRNA Synthesis Kit Plus 1	APExBIO	Cat#K1064
HyperScribe^{TM} T7 High Yield Cy5 RNA Labeling Kit	APExBIO	Cat#K1063
VAHTS RNA Clean Beads	Vazyme	Cat#N412-01
Cell Counting Kit-8	Dojindo Laboratories	Cat#CK04-01
sCelLive Tissue Digestion Solution	Singleron	Cat#1020012
FocuSCOPE Single Cell Multiomics mRNA × Mouse Liver Mutation Detection Kit	Singleron	Cat#4212122
GEXSCOPE Single Cell RNA Library Kit	Singleron	Cat#4180022
DynaSpatial FFPE Spatial Gene Expression Reagents Kit (for Mouse Transcriptome)	Dynamic Biosystems	Cat#13010060

(*Continued on next page*)

Continued		
REAGENT or RESOURCE	SOURCE	IDENTIFIER
DynaSpatial FFPE Spatial Gene Expression Slides	Dynamic Biosystems	Cat#13020031
Deposited data		
scRNA-seq/Target-seq/Spatial transcriptomics	This paper	NGDC: CRA022987 https://ngdc.cncb.ac.cn/gsa/browse/CRA022987
TCGA LIHC dataset	GDC Data Portal	TCGA: LIHC https://portal.gdc.cancer.gov/projects/TCGA-LIHC
GSE156625 dataset	Gene Expression Omnibus	GEO: GSE156625 https://www.ncbi.nlm.nih.gov/geo/query/acc.cgi?acc=GSE156625
Spatial transcriptome dataset from Liu's research	Mendeley Data	http://www.doi.org/10.17632/skrx2fz79n.1, https://data.mendeley.com/datasets/skrx2fz79n/1
Experimental models: Cell lines		
HEK293T	ATCC	Cat#CRL-3216
DC2.4	Merck	Cat#SCC142
Hepa 1-6	ATCC	Cat#CRL-1830
LLC	ATCC	Cat#CRL-1642
Experimental models: Organisms/strains		
C57BL/6 mouse (male, 6–8 week old)	Slac Laboratory Animal	N/A
Recombinant DNA		
PUC57-eGFP	This paper	N/A
PUC57-Neoantigens	This paper	N/A
PUC57-OVA	This paper	N/A
Software and algorithms		
ImageJ v2.17.0	National Institutes of Health (NIH)	https://imagej.net/software/fiji/
Living Image 4.2	PerkinElmer	https://www.perkinelmer.com
Fusion Software v2.2.0 (PhenoCycler-Fusion acquisition)	Akoya Biosciences	https://www.akoyabio.com/phenocycler/
inForm Tissue Analysis Software v3.0.0	Akoya Biosciences	https://www.akoyabio.com/phenoimager/inform-tissue-analysis-software/
GraphPad Prism 8.0	GraphPad software	https://www.graphpad.com/
Flow Jo 10.0	BD Bioscience	https://www.flowjo.com/
R v4.3.3	R Foundation	https://www.r-project.org/
Celescope v1.17.0	Singleron	https://github.com/singleron-RD/CeleScope
Seurat v5.1.0	Rahul Satija	https://satijalab.org/seurat/
Harmony v1.2.1	Ilya Korsunsky	https://portals.broadinstitute.org/harmony/
SingleR v2.4.1	SingleR-inc	https://github.com/SingleR-inc/SingleR
SCEVAN v1.0.1	Antonio De Falco	https://github.com/AntonioDeFalco/SCEVAN
CellChat v2.1.2	Cellchat	https://github.com/jinworks/CellChat
ClusterProfiler v4.10.1	YuLab-SMU	https://github.com/YuLab-SMU/clusterProfiler
DynamicST v1.0.4	Dynamic Biosystems	https://github.com/DynamicBiosystems/DynamicST
Stlearn v0.4.1	Duy Pham	https://stlearn.readthedocs.io/en/latest/
TESLA v1.2.4	Jian Hu	https://github.com/jianhuupenn/TESLA
Other		
Fluc-mRNA	TriLink	Cat#L-7202
RNase R	Beyotime	Cat#R7092M
RiboGreen RNA Reagent	Shanghai Maokang	Cat#MF0785
Red blood cell lysis	Solarbio	Cat#R1010
mm10 reference genome	Gencode	https://www.gencodegenes.org/mouse/

EXPERIMENTAL MODEL AND STUDY PARTICIPANT DETAILS

Cell lines

Human embryonic kidney 293T cells (HEK293T) and mouse dendritic cells (DC2.4) were employed to analyze the *in vitro* transfection efficiency and cellular uptake of LNP-mRNA. Luciferase-expressing murine hepatoma carcinoma cells (Hepa 1-6-Luc), which carry the seven neoantigens identified by our group,[16] were used to establish the orthotopic HCC model. Luciferase-expressing Lewis lung carcinoma cells (LLC-Luc) were used to generate liver metastasis model. Both luciferase-expressing lines were generated by lentiviral transduction. All cell lines were routinely tested for mycoplasma contamination and used within ten passages after thawing to ensure phenotypic stability and growth consistency.

Animals

All animal experiments were conducted in strict accordance with the guidelines approved by the Ethics Committee of Mengchao Hepatobiliary Hospital of Fujian Medical University (MCHH-AEC-2023-18). Male C57BL/6 mice (6–8 weeks old, 18–20 g) were purchased from Slac Laboratory Animal Co., Ltd (Shanghai, China). Mice were housed under specific pathogen-free (SPF) conditions with a 12 h light/12 h dark cycle at controlled temperature (22°C–25°C) and humidity (40–70%), with free access to irradiated chow and autoclaved water. Mice were examined to confirm good health before experiments; those with abnormal physiological conditions or failed tumor engraftment were excluded. No animals were lost or excluded due to death or technical failure. Group allocation was randomized, and all experiments followed standardized procedures to ensure reproducibility.

Human HCC tumor-infiltrating lymphocytes (TILs)

Tumor-infiltrating T lymphocytes (TILs) from two postoperative HCC patients (HCCTIL01 and HCCTIL02) were isolated and cultured following a previously reported method.[62] Both patients were male (54 and 55 years old) with hepatitis B virus-associated hepatocellular carcinoma and had not received prior anti-tumor treatment before surgery. Neither patient had evidence of other active infections or corticosteroid use, and both had good general condition and normal liver function (Child-Pugh class A) at the time of tumor resection. Briefly, freshly resected tumor fragments were cultured in X-vivo 15 medium supplemented with 6000 IU/mL IL-2 and 5% penicillin/streptomycin at 37°C under 5% CO_2. After 2 weeks, OKT3 (T&L biotechnology) was added for TIL expansion. The use of human HCC samples for TIL culture was approved by the Institutional Review Board of Mengchao Hepatobiliary Hospital of Fujian Medical University (Keshen 2021_100_02), and written informed consent was obtained from all participants.

METHOD DETAILS

mRNA preparation

The plasmids (pUC57-eGFP and pUC57-Neoantigens) were linearized by HindIII restriction enzyme (Thermo-Fisher) and used as templates for *in vitro* transcription. The Neo-mRNA construct encoded the codon-optimized sequences of seven long peptides previously identified in Hepa1-6[16] and LLC[48] cell lines with high H-2Kb-restricted immunogenicity. In parallel, an OVA-mRNA construct encoding the OVA240-269 epitope region was transcribed from a pUC57-OVA plasmid using the same procedure. *In vitro* transcription was performed using the HyperScribe All-in-One mRNA Synthesis Kit Plus 1 (APExBIO). The GFP-encoding mRNA ($mRNA^{GFP}$) was transcribed from the pUC57-eGFP plasmid using the same procedure and used as a reporter for *in vitro* transfection assays. For fluorescence labeling, the Cy5-mRNA was transcribed using HyperScribe T7 High Yield Cy5 RNA Labeling Kit (APExBIO). Transcribed mRNAs were purified with VAHTS RNA Clean Beads (Vazyme) according to the manufacturer's instructions. The luciferase-encoding mRNA ($mRNA^{Luc}$) was purchased from TriLink BioTechnologies and used as a reporter for *in vivo* assay.

LNP-mRNA preparation and characterization

The lipid nanoparticles (LNPs) were formulated from DOTMA (Xiamen Sinopeg Biotech Co., Ltd., Sinopeg) and DOPE (Sinopeg) at a 1:1 molar ratio using the ethanol-injection method (ethanol/water = 1/3, v/v), followed by ultrafiltration purification. LNP-mRNA complexes were prepared by vortex mixing pre-formed LNPs with mRNA at the indicated N/P ratios, calculated based on the molar ratio of cationic amines (DOTMA head group) to anionic phosphate groups of mRNA. The spleen-targeting neoantigen mRNA vaccine (STNvac) was prepared using DOTMA-DOPE LNPs and Neo-mRNA at N/P ratio of 0.5. The intramuscular control vaccine (IMNvac) was synthesized using a microfluidic mixing system (INano N, Micro&Nano, Shanghai) by combining a lipid phase (SM-102 (Sinopeg), DSPC (Sinopeg), cholesterol (Sinopeg), and DMG-PEG2000 (AVT (Shanghai) Pharmaceutical Tech Co., Ltd.) dissolved in ethanol) with an aqueous Neo-mRNA phase dispersed in 25 mM citrate buffer (pH = 4) at an ethanol-to-aqueous flow ratio of 1:3. The resulting IMN-LNPs were purified by ultrafiltration, buffer-exchanged into PBS, and stored at 4°C until use.

Particle morphology was visualized by transmission electron microscopy (TEM, Thermo-Fisher Talos L120C). Hydrodynamic size, polydispersity index, and zeta potential were measured using dynamic light scattering (DLS, Malvern Nano ZS).

mRNA release and degradation analysis

To evaluate mRNA release and degradation in serum-containing conditions, LNP-mRNA (N/*p* = 0.5) was incubated in RPMI 1640 medium supplemented with 10% FBS and RNase R (final concentration = 3 U/mL; Beyotime) at 37°C with constant shaking

(200 rpm). At designated time points (0, 1, 2, 3, 6, 12, 24, 48, and 72 h), aliquots were collected for analysis. For agarose gel electrophoresis, samples were either directly loaded or pretreated with heparin sodium (final 10 mg/mL; Achem-block) for 15 min at room temperature to displace mRNA from LNPs. Gels (1% agarose, Super GelBlue staining) were run at 120V for 20 min and imaged using a Gel Doc XR system (Bio-Rad). For quantitative assessment of RNA release, the RiboGreen RNA Reagent (Shanghai Maokang) was used to measure total RNA content following heparin treatment. Fluorescence was recorded on a SpectraMax iD3 microplate reader (Molecular Devices) and quantified using a standard curve prepared from serial dilutions of purified mRNA. Release at each time point was calculated by normalizing RNA amount to the 0 h time point and expressed as the percentage of RNA released over time.

In vitro experiments

The GFP-encoding mRNA ($mRNA^{GFP}$) was used as the reporter gene to evaluate transfection efficiency, cytocompatibility and formulation stability *in vitro*. Cellular viability following LNP-mRNA exposure was assessed in HEK293T and DC2.4 cells using the Cell Counting Kit-8 (Dojindo Laboratories) in 96-well plates with 0.1 μg mRNA per well, and absorbance was measured after 24 or 48 h of incubation. For transfection assays, cells were seeded in 24-well plates and treated with LNP-mRNA (1 μg mRNA per well) for 24 h. GFP expression was quantified by flow cytometry (BD FACSVerse) and visualized by fluorescence microscopy (Nikon Intensilight C-HGFI). To evaluate serum stability, freshly prepared LNP-$mRNA^{GFP}$ and samples pre-incubated in 10% FBS at 4°C for 24 h (N/*p* = 0.5) were compared under identical transfection conditions. DC2.4 cells were analyzed 24 h post-transfection for GFP expression by flow cytometry and fluorescence microscopy. Cellular uptake was examined in DC2.4 cells using Cy5-labeled LNP-mRNA (N/*p* = 2). After incubating for 2–6 h, cells were fixed with paraformaldehyde (4%) and imaged by confocal laser scanning microscopy (Zeiss LSM780).

In vivo and splenic immune-cell distribution of mRNA expression

Luciferase-encoding mRNA ($mRNA^{Fluc}$) was used as the reporter gene to assess *in vivo* and immune cell-level biodistribution of LNP-mRNA formulations. Formulations with N/P ratios ranging from 0.5 to 5 were intravenously injected into C57BL/6 mice (*n* = 3 per group, 10 μg of mRNA per mouse). After 6 h, luciferase substrate (20 mg/mL, 150 μL per mouse; PerkinElmer) was administered intraperitoneally, and whole-body and *ex vivo* bioluminescence imaging of major organs (heart, liver, spleen, lung, kidney) was performed using an IVIS Spectrum Imaging System (PerkinElmer).

For biodistribution analysis in tumor-bearing mice, orthotopic HCC models were established by intrahepatic implantation of Hepa1-6 (luc-negative) cells, and LNP-$mRNA^{Fluc}$ (N/*p* = 0.5, 10 μg mRNA per mouse) was administered intravenously 1 week after tumor implantation. Major organs were harvested 6 h post-injection for *ex vivo* bioluminescence imaging and corresponding bright-field photography.

For immune cell-level biodistribution, spleens were mechanically dissociated to obtain single-cell suspensions, followed by red blood cell lysis (Solarbio). Aliquots of each sample were used for separate staining panels, with surface markers (CD11c, CD11b, CD19, CD3, and NK1.1; eBioscience) to identify DCs, macrophages, B cells, T cells, and NK cells, respectively. After fixation and permeabilization, cells were stained with PE-conjugated anti-firefly luciferase antibody (Abcam) to detect luciferase expression. Samples were analyzed by flow cytometry (BD FACSVerse) to determine the composition of total splenic immune cells and the relative proportions of each subset within luciferase-positive (Luc^+) populations.

For immunofluorescence validation, frozen spleen sections were collected 6 h after LNP-$mRNA^{Cy5}$ injection, stained with Hoechst 33342 (Sigma-Aldrich) and anti-CD11c antibody (eBioscience), and imaged using confocal laser scanning microscopy (Zeiss LSM780) to visualize the spatial colocalization of $CD11c^+$ DCs with Cy5-labeled mRNA signals. The distribution of $CD11c^+$ cells was quantified from representative images using ImageJ software.

Ex vivo IFN-γ ELISpot assay

The immunogenicity of STNvac was evaluated using an *ex vivo* IFN-γ ELISpot assay (Mouse IFN-γ ELISpot Kit; Mabtech). Bone marrow-derived dendritic cells (BMDCs) isolated from non-vaccinated mice were pulsed with neoantigen peptides (1×10^5 cells and 4 μg peptide per well) for 24 h in the ELISpot plate. Splenic T cells, isolated from mice 3 days after a single STNvac immunization (10 μg Neo-mRNA per mouse), were then co-cultured with peptide pre-treated BMDCs (5×10^4 T cells per well) for an additional 24 h. IFN-γ spots were detected according to the manufacturer's instructions and quantified using the ELISpot Analysis System (Antai Yongxin Medical Technology, AT-Spot-2200).

Therapeutic efficacy in orthotopic HCC

Orthotopic HCC models were established by surgically implanting 3×10^5 Hepa1-6-Luc cells into the liver lobe of male C57BL/6 mice (week 0). On the seventh day post-tumor inoculation (week 1), mice received intravenous STNvac following a three-dose regimen (10 μg Neo-mRNA on day 0 and 5 μg on days 3 and 7; total 20 μg per mouse). Tumor progression was monitored weekly using the IVIS Spectrum Imaging System (PerkinElmer), and total photon flux was quantified using Living Image 4.2 software. Three days after the final STNvac administration, tumors were excised for flow cytometry, multiplex immunofluorescence, and ELISA (IL-12, IFN-γ, and TNF-α; Boster Bio). Whole blood was collected in EDTA-2K tubes for hematology, and major organs were processed for H&E staining to assess histopathology.

For dose-regimen optimization, vaccination schedules comprising single (1×), double (2×), triple (3×), and quadruple (4×) intravenous administrations were compared using a fixed total Neo-mRNA dose of 20 μg per mouse (n = 5 per group). The peptide-based vaccine (Pep-NeoVac, formulated with Poly I:C) was used as a reference control and administered subcutaneously in a three-dose regimen (2 μg per peptide, total 14 μg) at comparable intervals (days 0, 4, and 8), following the protocol established in our previous study.[16]

For route comparison, an intramuscular control vaccine (IMNvac) was prepared using an SM-102-based LNP formulation equivalent to that employed in Moderna's mRNA-1273 platform. IMNvac encapsulated the same Neo-mRNA as STNvac and was administered intramuscularly following the same three-dose schedule (days 0, 3, and 7).

For spleen-dependency evaluation, surgical splenectomy was performed under anesthesia immediately before intrahepatic tumor implantation during the same surgical session. Mice were then treated with PBS or STNvac according to the same three-dose schedule as above.

For combination therapy, an anti-PD-1 monoclonal antibody (αPD-1, Leinco, clone RMP1-14) was administered intraperitoneally 24 h after each STNvac injection at a dose of 200 μg per mouse to assess potential synergy.

Prophylactic and metastatic models

For prophylactic evaluation, healthy mice were immunized with STNvac (10 μg on day-12; 5 μg on days −9 and −5) and subsequently challenged intrahepatically with 3×10^5 Hepa1-6-Luc cells on day 0. For the liver-metastasis model, 1×10^5 LLC-Luc cells were implanted intrahepatically on day −5, followed by STNvac treatment on days 0, 3, and 7.

Mechanistic validation of the GZMA-F2R axis

To assess the functional role of GZMA-F2R signaling, the F2R antagonist SCH79797 (25 μg/kg; MCE) was administered 24 h after each STNvac dose to assess the contribution of the GZMA-F2R axis to vaccine efficacy. The dosing regimen and intravenous administration route were selected with reference to previously published studies[63–65] and the manufacturer's recommendations, with minor adjustments made according to experimental feasibility and the physicochemical properties of F2RA.

For cross-tumor validation, an orthotopic intrahepatic cholangiocarcinoma (ICC) model was established by surgically implanting 5×10^5 KPC-OVA-Luc cells (KrasG12D; Trp53R172H; Pdx1-Cre; expressing OVA and luciferase) into the liver of C57BL/6 mice. Mice received PBS, OVA-mRNA STNvac, or OVA-STNvac+F2RA (SCH79797), and were monitored for 6 weeks by IVIS Spectrum imaging and survival analysis.

Systemic safety evaluation

For biosafety assessment, tumor-free C57BL/6 mice received intravenous STNvac on days 0, 3, and 7. Blood samples were collected via orbital capillary sampling at days 0, 3, 10, 21, and 35. To minimize distress and prevent excessive sampling within short intervals, mice were divided into two alternating cohorts, each bled no more than once every 7 days. Cytokine concentrations (IL-1β, IL-6, TNF-α, and IFN-β ELISA kits; Boster Bio), complement levels (C3a and C5a ELISA kits; Cusabio Biotech), and total IgG levels (mouse IgG ELISA kit; Boster Bio) were quantified according to the manufacturers' instructions. Serum biochemistry (liver and kidney function) was analyzed on day 35 (n = 6 per group). Major organs (heart, liver, spleen, lung, and kidney) were fixed in 4% paraformaldehyde and processed for H&E staining.

Pharmacokinetic and biodistribution analysis of F2RA

F2R antagonist (F2RA, SCH79797; MCE) was dissolved in a minimal volume of DMSO and diluted in PBS (final injection volume 200 μL) for intraperitoneal administration at 0.1 mg/kg. The dosing regimen and vehicle preparation were adapted from previous reports and manufacturer's recommendations.[63–65] Blood and tissue samples were collected at the indicated time points (0.25–6 h for plasma; 0.5 h for organs). F2RA concentrations were quantified by LC-MS/MS coupled with HPLC (AB Sciex API 5500 system, Shimadzu LC-30AD UPLC) as previously described.[65] Plasma concentration-time profiles and organ distribution were calculated to assess systemic exposure and tissue enrichment.

Flow cytometry of mouse spleen and tumor cells

Single-cell suspensions from mouse spleen and tumor tissues were prepared after the indicated treatments. To evaluate the immune activation status, cells were stained for surface markers corresponding to DCs and T cells, including CD11c (eBioscience), CD80 (eBioscience), CD86 (eBioscience), CD3 (eBioscience), CD4 (BioLegend), CD8 (BioLegend), and CD69 (BioLegend). For profiling splenic immune-cell subsets and luciferase-expressing populations, additional panels including CD11b (eBioscience), NK1.1 (eBioscience), and CD19 (eBioscience) were applied to identify macrophages, NK cells, and B cells, respectively. For neoantigen-specific $CD8^+$ T cell detection, cells were stained with CD8 (FITC, eBioscience) and a PE-labeled Ptpn2-MHC tetramer (prepared by the BioReagent Unit, Cancer Research Center of Xiamen University). For immune-memory evaluation, splenic cells were stained with CD3 (eBioscience), CD8 (PE, eBioscience), CD44 (eBioscience) and CD62L (eBioscience).

For intracellular detection of firefly luciferase and IFN-γ, cells were fixed and permeabilized using the Fixation/Permeabilization Solution Kit (BD Bioscience) before staining with anti-luciferase (Abcam) and anti-IFN-γ (eBioscience). For *in vivo* MHC-I blockade experiments, tumor-bearing mice were administered an anti-H-2Kb blocking antibody (MCE; 200 μg per mouse per injection) or an

isotype control (BioXCell; 200 μg per mouse per injection) via intraperitoneally injection 24 h prior to each STNvac vaccination. Tumors were collected 3 days after the final vaccination, and single-cell suspensions were prepared and stained with surface markers including CD45 (eBioscience), CD8 (eBioscience) and CD69 (eBioscience), followed by intracellular staining for ISG15 using a primary anti-ISG15 antibody (Abcam) and a fluorophore-conjugated secondary antibody (CST), to assess the activation status of $ISG15^+$ $CD8^+$ T cells. Flow cytometric data were acquired on a BD FACSVerse and analyzed using FlowJo software.

Flow cytometry of human HCC TILs

To evaluate the impact of the GZMA-F2R signaling on $CD8^+$ TIL activation, the in vitro-expanded TILs were seeded in 24-well plates, pre-treated with the F2R antagonist SCH79797 (MCE, 5 μM), and subsequently stimulated with recombinant GZMA (MCE, 0.05 ng/mL). After 24 h, TIL activation was quantified by flow cytometry. F2R surface expression was detected using an indirect staining protocol with a primary PAR1 monoclonal antibody (Invitrogen) followed by a fluorescent secondary antibody (Proteintech). For activation profiling on $CD3^+$ $CD8^+$ TILs, cells were stained with CD3 (eBioscience), CD8 (eBioscience), 41BB (eBioscience) and PD-1 (eBioscience) according to the manufacturers' instructions. Human TILs derived from two postoperative HCC patients (HCCTIL01 and HCCTIL02) were expanded *in vitro* and divided into triplicate wells for each stimulation condition. Flow cytometric analyses were performed independently for each replicate, and averaged values were used to represent individual donors.

Histology and immunostainings

Tumors harvested from the orthotopic HCC mouse model after the indicated treatment were processed into paraffin sections for histology and immunofluorescence analysis. Hematoxylin and eosin (H&E) staining was performed using H&E Stain Kit (Solarbio) for histopathology analysis. Ki67 immunohistochemistry (Servicebio) and TUNEL assays (One Step TUNEL Apoptosis Assay Kit, Beyotime) were conducted to evaluate tumor cell proliferation and apoptosis. For two-color immunofluorescence, sections were stained with anti-CD4 (Servicebio) and anti-CD8 (Servicebio) antibodies to visualize intratumoral T cell infiltration.

Multi-color immunofluorescence staining was performed using tyramide signal amplification (TSA) technology. Formalin-fixed paraffin-embedded tumor sections were deparaffinized, rehydrated, and subjected to antigen retrieval. After quenching and blocking, primary antibodies were sequentially incubated and detected with HRP-conjugated secondary antibodies and fluorophore-labeled tyramide reagents (AiFang Biological, Hunan, China). After each staining cycle, antibody complexes were removed by heat-mediated stripping before proceeding to the next marker. Slides were counterstained with DAPI, mounted with anti-fade medium, and imaged using the PhenoCycler-Fusion system (Akoya Biosciences) controlled by Fusion software (v2.2.0). The following antibody panels were applied:

Panel 1 (T cell effector function): CD8 (CST), ISG15 (Invitrogen), IFN-γ (GeneTex), and GZMB (AiFang Biological).

Panel 2 (T cell activation and proliferation): CD8 (CST), ISG15 (Invitrogen), CD69 (Proteintech), and Ki67 (Abcam).

Panel 3 ($GZMA^+$ APC): GZMA (Proteintech), CD11c (AiFang Biological), CD4 (AiFang Biological), and CD19 (AiFang Biological).

Panel 4 (neoantigen-specific T cells and mature TLS): CD8 (CST), ISG15 (Invitrogen), CD20 (Abcam), and CD23 (Abcam).

Panel 5 (TLS structural markers): CD20 (Abcam), CD21 (Abcam), CD23 (Abcam), and CD34 (AiFang Biological).

Panel 6 (B-cell maturation and activation): CD20 (Abcam), Bcl6 (Invitrogen), CD138 (Proteintech), CD27 (Abcam), and CD86 (AiFang Biological).

Image quantification was performed using inForm software (v3.0.0, Akoya Biosciences) to identify positive cells, calculate marker co-localization, and quantify regional immune-cell densities.

Single cell data processing

Three tumor samples from both STNvac and PBS (control) groups were collected and subjected to single-cell suspension with 2 mL of sCelLive Tissue Digestion Solution (Singleron, China). Cell viability was assessed using the trypan blue exclusion assay (Gibco) to ensure a viability of greater than 80%, and then the concentration was adjusted to 3×10^5 cells/mL with PBS. Single cell isolation and mRNA capture were conducted on Singleron Matrix Single Cell Processing System (Singleron, China), and the single cell RNA sequencing (scRNA-seq) library and single cell target RNA sequencing library were constructed using the FocuSCOPE Single Cell Multiomics mRNA x Mouse Liver Mutation Detection Kit (Singleron, China) according to the manufacturer's instructions. The library was sequenced on the Illumina HiSeq 6000 platform (150 bp paired end reads). Sequencing data was preprocessed with Celescope (v1.17.0) and aligned to mm10 reference genome to generate single cell expression matrix and single cell mutation expression matrix. Cells with fewer than 200 detected genes or a number of detected genes above the 98th percentile for the corresponding sample were excluded from downstream analysis. Filtered single cell expression matrix for different samples was then merged with Seurat (v5.1.0) and the batch effect was removed using the Harmony algorithm (v1.2.1). Cells were then clustered according to their expression patterns and annotated with SingleR (v2.4.1), followed by manual correction based on known marker-gene expression. To further distinguish tumor cells, SCEVAN (v1.0.1) was applied to identify the copy number profiles of each cell based on the expression patterns and then classify malignant cells from non-malignant cells. Then the single cell mutation expression matrixes were filtered to retain only tumor cells and the overall expression of neoantigen derived mutations were calculated as the number of expressing mutations within each cell. CellChat (v1.6.1) was applied to identify intercellular communication between different cell types and compare the communication patterns between the two samples.

To further investigate the immune microenvironment after STNvac treatment, single cell RNA sequencing was further performed on $CD45^+$ cells sorted by flow cytometry from isolated tumor tissues. Similarly, single cells were isolated and mRNA was captured, and the scRNA-seq library was constructed using the GEXSCOPE Single Cell RNA Library Kit (Singleron, China). Sequencing data was preprocessed, merged and annotated like previously described. T cells were then extracted and further subjected to more detailed clustering to identify functional T cell subpopulations based on key functional gene expressions. Marker genes for each T cell cluster were identified using FindAllMarkers in Seurat. The top 10 marker genes of each cluster were then visualized with DoHeatmap. Differentially expressed genes between $ISG15^+$ $CD8^+$ T cells from STNvac and control groups were also identified with FindAllMarkers. Pathway enrichment (KEGG) enrichment and Gene Ontology analyses were performed using ClusterProfiler (v4.10.1). Likewise, CellChat was applied to explore the interaction between $ISG15^+$ $CD8^+$ T cells and other cells.

Spatial transcriptome data analysis

Tumor samples from both STNvac and control groups were analyzed using the DynaSpatial FFPE Spatial Gene Expression Reagents Kit (for Mouse Transcriptome, Dynamic Biosystems, Suzhou, China) to extract spatial features. The FFPE tissue sections were cut and then placed on slides, which were subjected to H&E staining. The sequencing library was prepared using DynaSpatial FFPE Spatial Gene Expression Slides & Reagents Kits (Dynamic Biosystems, including DynaSpatial FFPE Spatial Gene Expression Slides and DynaSpatial FFPE Gene Expression Reagents Kit) and subjected to quality control. The sequencing process was then performed with BGI Genomics DNBSEQ-T7 (BGI Genomics).

Sequencing reads of spatial transcriptome were aligned to mm10 reference genome using DynamicST (v1.0.4) to generate count matrix corresponding to spots on the sampling tissues. The matrix was first processed with stlearn (v0.4.1) to perform spot clustering for identifying spots with similar expression patterns. To further enhance the resolution of spatial transcriptome data, TESLA (v1.2.4) was deployed. In brief, drawContours was first applied to extract tissue regions from the image for downstream super-resolution processing and region annotation; TESLA was then applied to the original count matrix with size of the superpixel (res) set to 10 to generate expression matrix with enhanced resolution. Tissue regions containing selected cell types were annotated using corresponding marker genes and visualized with customized color scale. The percentage of positive spots in each sample for selected cell types was calculated as the quotient of the number of positive spots to the total number spots within each sample. Regions of interest were further separated from the visualization results to highlight them.

Public dataset analysis

TCGA: LIHC dataset was obtained from GDC Data Portal and patients were stratified according to the ssGSEA scores of the signature containing CD8A, CD8B and ISG15, the survival analysis was then further conducted using survival packages in R.

Mendeley Data: http://www.doi.org/10.17632/skrx2fz79n.1 (the spatial transcriptome dataset from Liu's research[56]) was downloaded with the link provided in that publication. Spots with cells of interest were annotated based on the expression of corresponding cell marker genes ($ISG15^+$ $CD8^+$ T cells: CD8A, ISG15 and F2R; $GZMA^+$ B cells: MS4A1 and GZMA; $GZMA^+$ CD4 T cells: CD4 and GZMA; $GZMA^+$ DCs: ITGAX and GZMA). TLS was identified as spots with co-localization of B cells, $CD4^+$ T cells, DCs and CXCL13 expressions.

The single cell transcriptome dataset GEO: GSE156625[55] was obtained from NCBI website and subjected to downstream analysis. Cells were clustered and different cell types were annotated with SingleR. $F2R^+$ $CD8^+$ T cells and $GZMA^+$ APCs were extracted. The cell interaction between $F2R^+$ $CD8^+$ T cells and $GZMA^+$ APCs was analyzed and visualized with CellChat.

QUANTIFICATION AND STATISTICAL ANALYSIS

All quantitative data are expressed as the mean ± standard deviation (SD). Statistical analyses were performed using GraphPad Prism 8.0. Differences between two groups were analyzed using an unpaired two-tailed Student's *t* test, and comparisons among multiple groups were evaluated by one-way analysis of variance (ANOVA). Survival curves were analyzed using the Kaplan-Meier method with the Log Rank (Mantel-Cox) test. Statistical significance was defined as *p*-value (pval) < 0.05 (*$p < 0.05$, **$p < 0.01$, ***$p < 0.001$, and ****$p < 0.0001$). The number of biological replicates (n) for each experiment is indicated in the corresponding figure legends.

Cell Reports Medicine, Volume *7*

Supplemental information

Spleen-targeted neoantigen mRNA vaccine induces ISG15$^+$ CD8$^+$ T cell-mediated tertiary lymphoid structure formation in hepatocellular carcinoma

Xinyi Lin, Geng Chen, Ruijing Tang, Ming Wu, Da Zhang, Fangzhou Lin, Jianhua Guan, Jing Yang, Xiuqing Dong, Xiaoyuan Zheng, Liman Qiu, Haijun Yu, Zhixiong Cai, and Xiaolong Liu

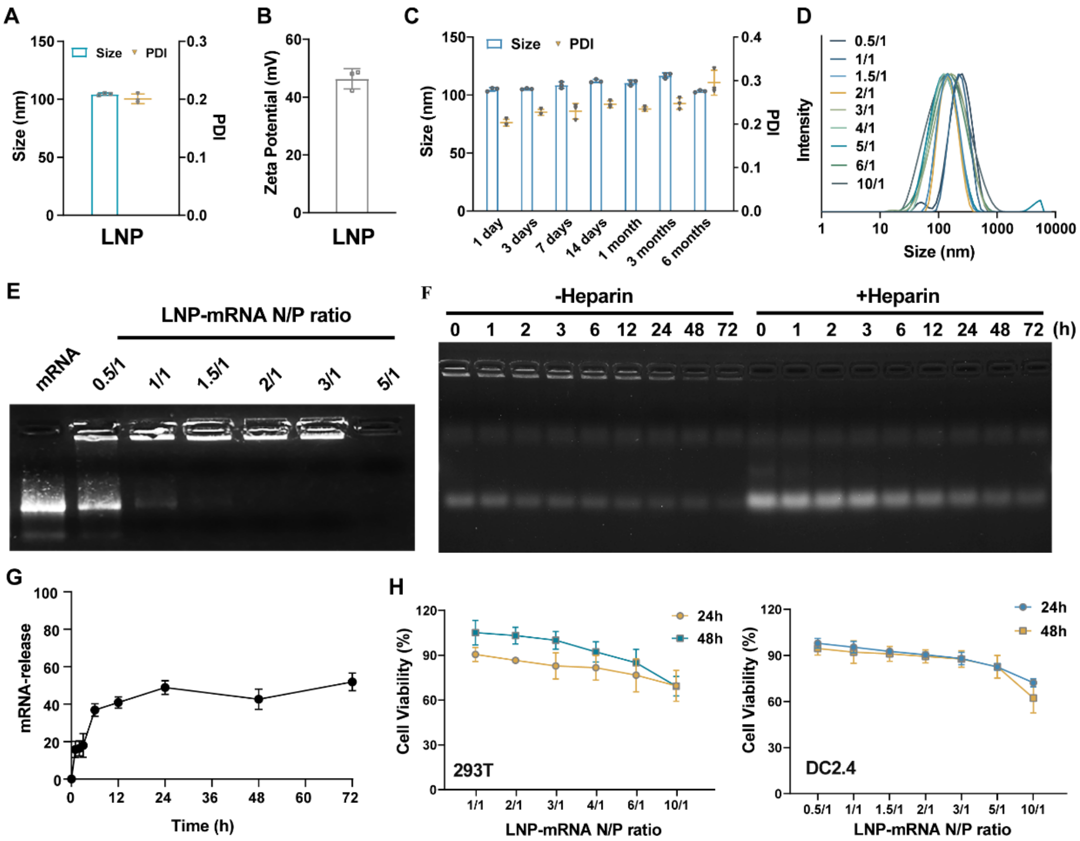

Figure S1. Synthesis and characterization of LNP-mRNA. Related to Figure 1.

(A) Particle size (104.4 nm) and polydispersity index (PDI = 0.201) of DOTMA/DOPE LNPs (n = 3 independent preparations).

(B) Zeta potential of pure LNPs (46.3 mV), indicating strong electrostatic capacity for complexing negatively charged mRNA (n = 3 independent preparations).

(C) Particle size and PDI of LNPs during storage at 4 °C for 6 months, showing minimal change and confirming colloidal stability (n = 3 independent preparations).

(D) Representative particle size distribution of LNP-mRNA complexes at different N/P ratios.

(E) Agarose gel electrophoresis of LNP-mRNA at different N/P ratios, with naked mRNA as a migration reference. Complete mRNA retention was observed at N/P ≥ 1.5.

(F) Agarose gel analysis of LNP-mRNA (N/P = 0.5) incubated in 10% serum-containing medium supplemented with RNase R (final 3 U/mL) at 37 °C with orbital shaking (200 rpm) for 0-72 h. Untreated lanes (left) show a gradual loss of both retained and migrating mRNA signals, whereas heparin-treated lanes (final 10 mg/mL) reflect the total recoverable mRNA (right). A molecular size marker was not included because the assay evaluates relative mRNA release and protection. Representative result from 3 independent biological replicates.

(G) Quantitative analysis of mRNA release measured by RiboGreen fluorescence after heparin displacement (final 10 mg/mL). An initial burst release (~35%) occurred within 6 h, reaching a plateau (~50%) by 24 h, indicating that a substantial fraction of mRNA remains LNP-associated despite serum nuclease exposure. Data are presented as mean ± SD (n = 3 biological replicates).

(H) Cytotoxicity of LNP-mRNA in 293T and DC2.4 cells assessed by CCK-8. Data are presented as mean ± SD (n = 5 biological replicates). LNP-mRNA showed good biocompatibility across N/P ratios tested, except at N/P = 10, where a moderate reduction in cell viability was observed.

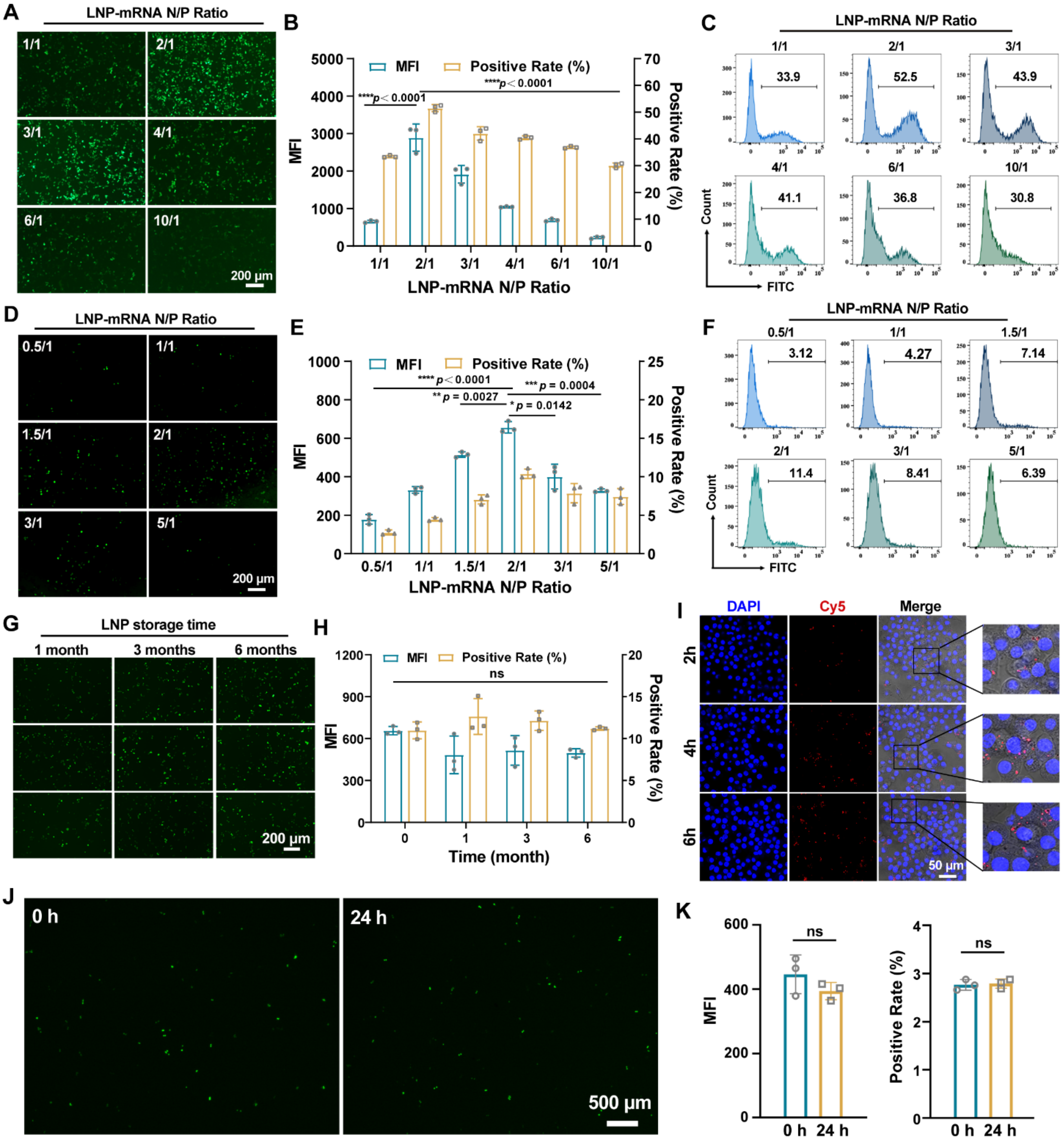
A
LNP-mRNA N/P Ratio
1/1
2/1
3/1
4/1
6/1
10/1
200 μm
B
MFI
Positive Rate (%)
****p < 0.0001
****p < 0.0001
LNP-mRNA N/P Ratio
C
LNP-mRNA N/P Ratio
33.9
52.5
43.9
41.1
36.8
30.8
Count
FITC
D
LNP-mRNA N/P Ratio
0.5/1
1.5/1
5/1
E
**** p < 0.0001
*** p = 0.0004
** p = 0.0027
* p = 0.0142
F
3.12
4.27
7.14
11.4
8.41
6.39
G
LNP storage time
1 month
3 months
6 months
H
ns
Time (month)
I
DAPI
Cy5
Merge
2h
4h
6h
50 μm
J
0 h
24 h
500 μm
K
ns
Positive Rate (%)

Figure S2. In vitro performance of LNP-mRNA. Related to Figure 1.

For all in vitro experiments, mRNA encoding GFP ($mRNA^{GFP}$) was used as the reporter, and cells were analyzed 24 h post-transfection unless otherwise noted.

(A-C) Transfection of 293T cells with LNP-$mRNA^{GFP}$ at indicated N/P ratios. (A) Representative fluorescence microscopy images. Scale bar, 200 µm. (B) Flow cytometry quantification of mean fluorescence intensity (MFI) and GFP-positive cell percentage, showing peak efficiency at N/P = 2. (C) Representative FITC histograms.

(D-F) Transfection of DC2.4 cells with LNP-$mRNA^{GFP}$ at indicated N/P ratios. (D) Representative fluorescence microscopy images. Scale bar, 200 µm. (E) Flow cytometry quantification of MFI and GFP-positive cell percentage, showing peak efficiency at N/P = 2. (F) Representative FITC histograms.

(G-H) Transfection efficiency of LNP-$mRNA^{GFP}$ (N/P = 2) in DC2.4 cells using LNPs stored at 4 °C for 1-6 months. (G) Representative fluorescence microscopy images. Scale bar, 200 µm. (H) Flow cytometry quantification showing MFI and GFP-positive cell percentage, indicating that long-term storage does not impair delivery efficiency.

(I) Confocal laser scanning microscopy (CLSM) images showing intracellular uptake of LNP-$mRNA^{Cy5}$ (N/P = 2) in DC2.4 cells after 2-6 h of incubation. Scale bar, 50 µm.

(J-K) Transfection of DC2.4 cells with freshly prepared or serum-exposed LNP-$mRNA^{GFP}$ (10% serum, 4 °C, 24 h; N/P = 0.5). (J) Representative fluorescence microscopy images. Scale bar, 500 µm. (K) Flow cytometry quantification showing MFI and GFP-positive cell percentage 24 h post-transfection. Brief serum exposure did not significantly affect transfection efficiency, indicating that DOTMA/DOPE LNPs retain structural integrity and delivery function under short-term handling conditions.

Statistics: Data are presented as mean ± SD (n = 3 biological replicates for all quantifications). For N/P ratio comparisons (B, E), one-way ANOVA was performed (comparing N/P = 2 vs. other ratios). For LNP storage comparisons (H), one-way ANOVA was used to assess differences among time points (1-6 months). For serum-exposure comparisons (K), a two-tailed paired t-test was applied (fresh vs. 24 h serum-exposed within each replicate). Significance levels: *$p < 0.05$; **$p < 0.01$; ***$p < 0.001$; ****$p < 0.0001$.

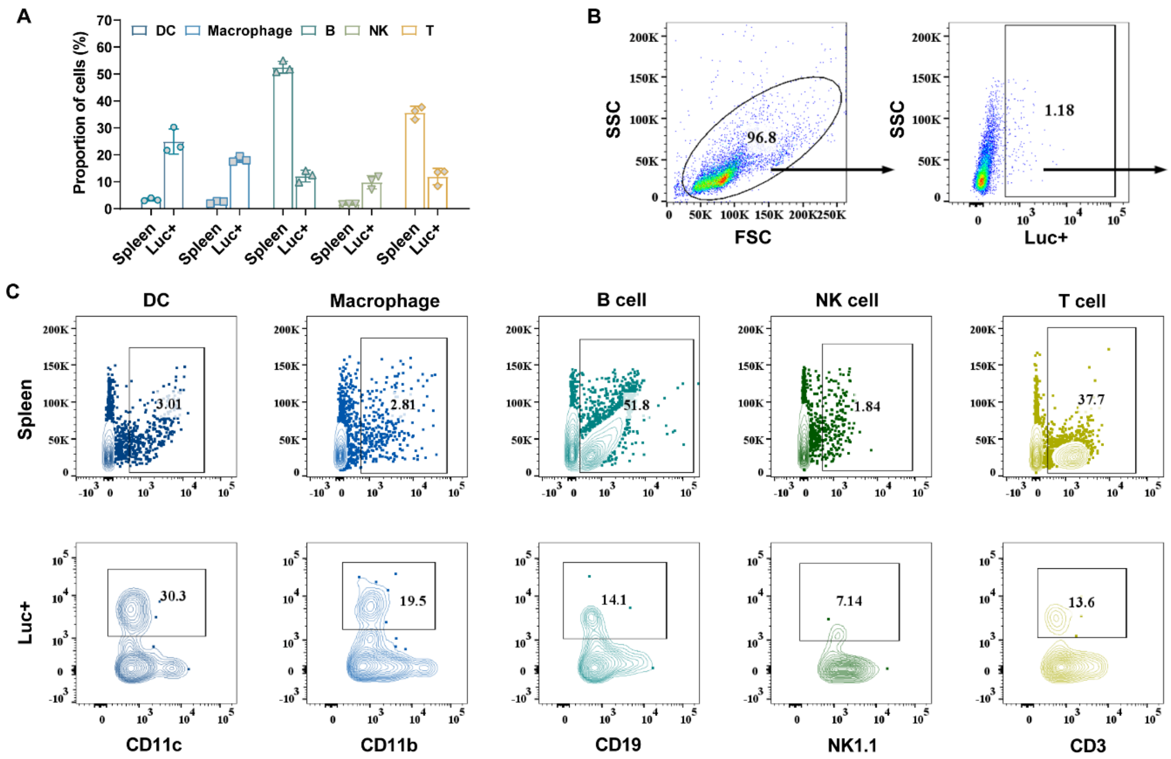

Figure S3. Cellular composition of the spleen and distribution of luciferase expression among immune subsets after LNP-mRNA administration. Related to Figure 2.

Firefly luciferase mRNA (mRNA^{Fluc}) was used as the reporter. Splenic single-cell suspensions were prepared by mechanical dissociation followed by red blood cell lysis and analyzed 6 h post-administration.

(A) Flow cytometric quantification of major splenic immune cell populations (DCs, macrophages, B cells, NK cells, and T cells) and their respective proportions within luciferase-positive (Luc+) cells. Despite their low abundance in the spleen (DCs 3.42%, macrophages 2.46%), DCs accounted for the highest proportion of Luc+ cells (24.97%), followed by macrophages (18.33%), B cells (12.15%), T cells (11.94%), and NK cells (9.85%), indicating preferential transfection of professional antigen-presenting cells by DOTMA/DOPE LNPs. Data are presented as mean ± SD (n = 3 biological replicates).

(B) Representative gating strategy for identifying Luc+ cell subsets from splenic single-cell suspensions.

(C) Representative density plots showing the indicated immune subsets in total splenic versus Luc+ populations. Luciferase expression was detected by intracellular staining with PE-conjugated anti-firefly luciferase antibody after cell fixation and permeabilization.

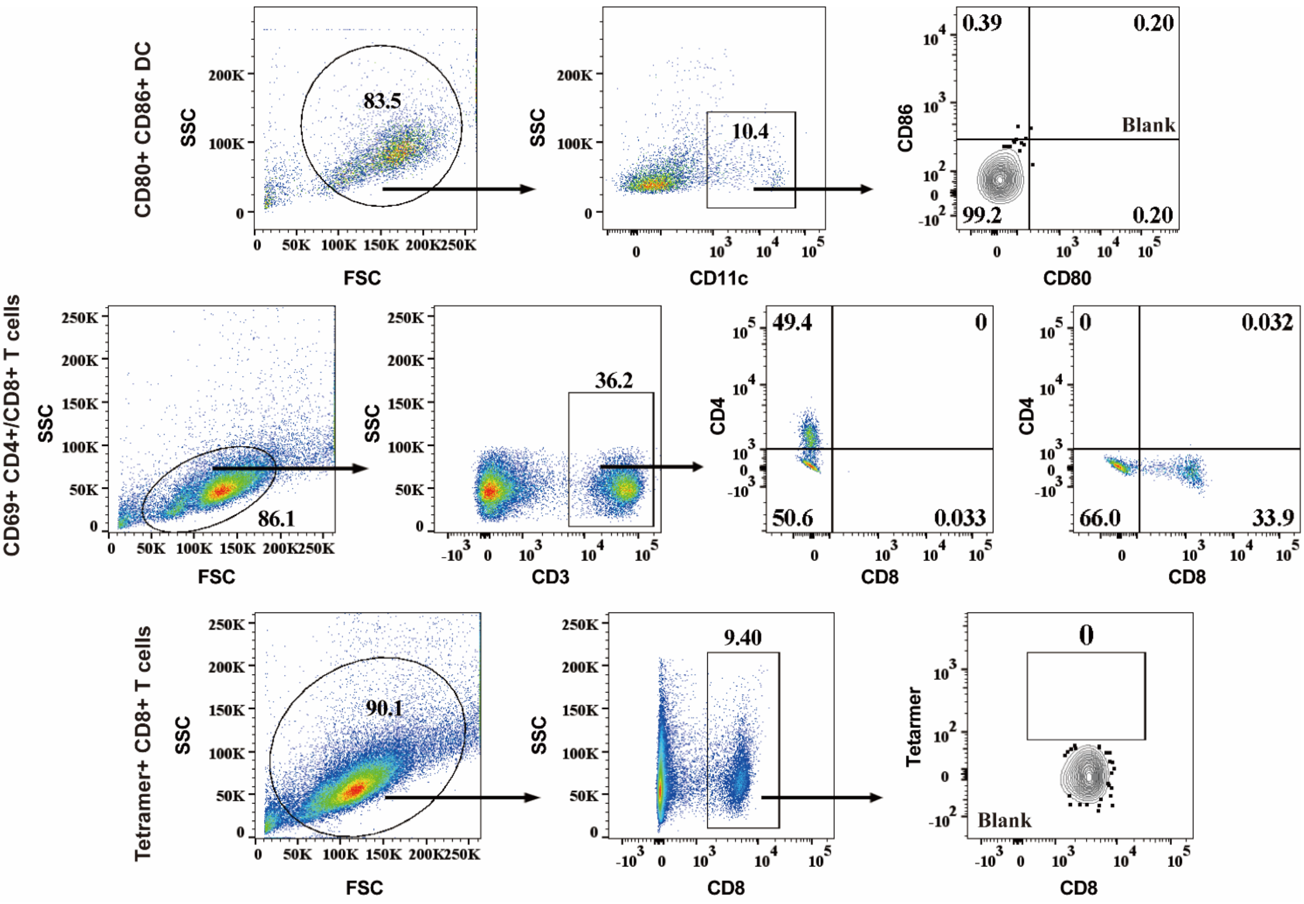

Figure S4. Flow cytometry gating strategy for assessing splenic immune cell populations. Related to Figure 2E. Representative gating sequence used to identify and quantify splenic CD80+ CD86+ DCs, CD69+ CD4+ T cells, CD69+ CD8+ T cells, and Ptpn2-specific CD8+ T cells after STNvac administration.

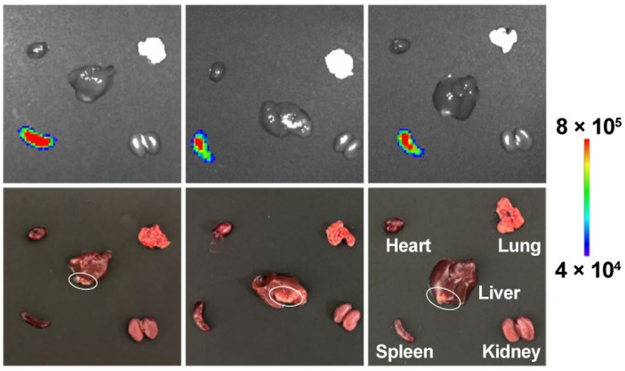

Figure S5. In vivo biodistribution of mRNA expression in orthotopic HCC-bearing mice. Related to Figure 3. Ex vivo bioluminescence imaging (top) and corresponding bright-field photographs (bottom) of major organs (heart, liver (HCC-bearing), spleen, lung, and kidney) collected 6 h after intravenous administration of LNP-mRNA^{Fluc} (N/P = 0.5; 10 µg mRNA per mouse) in orthotopic HCC-bearing C57BL/6 mice one week after tumor implantation. The tumor sites on the liver were outlined with white circles in the bright-field images. Predominant luciferase expression was observed in the spleen.

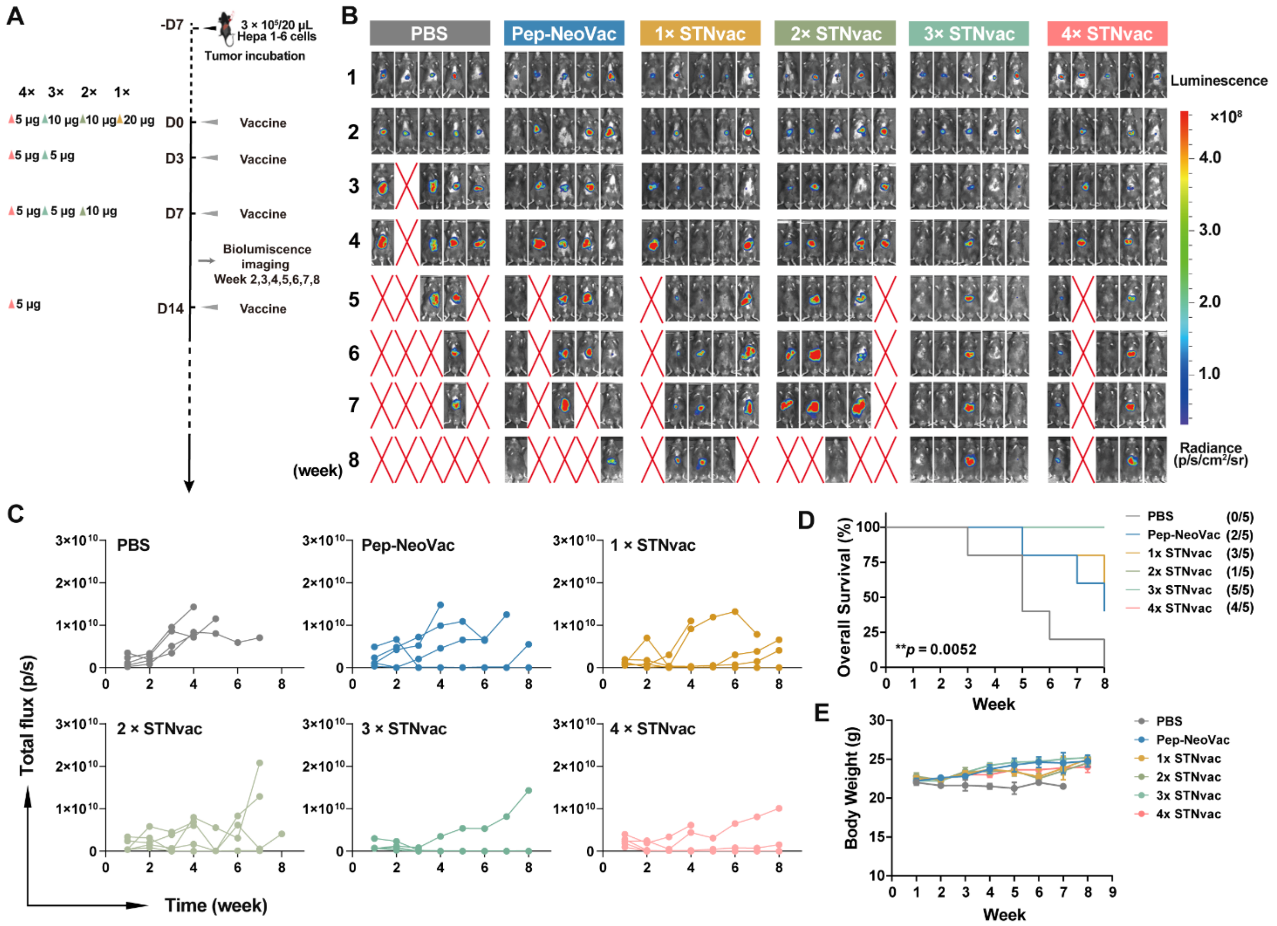

Figure S6. Optimization of STNvac vaccination regimen. Related to Figure 3.

(A) Schematic illustration of the vaccination protocol comparing single (1×), double (2×), triple (3×), and quadruple (4×) administrations with a fixed total mRNA dose of 20 μg per mouse (n = 5 mice per group). The peptide-based vaccine (Pep-NeoVac, formulated with Poly I:C) was administered subcutaneously in a three-dose regimen (2 μg per peptide, total 14 μg) at comparable intervals (day 0, 4, and 8), following the protocol established in our previous study (J Immunother Cancer, 2022, 10, e004389).

(B) Bioluminescence images showing tumor burden in orthotopic HCC-bearing mice during an 8-week observation period. Tumor growth in the PBS group was rapid, whereas varying degrees of regression were observed in all STNvac-treated groups. The 3× group showed the highest progression-free survival (PFS) rate of 80%, while the PFS rates for the PBS, 1×, 2×, and 4× groups were 0%, 20%, 20%, and 40%, respectively. Across all regimens, STNvac consistently outperformed the peptide-based vaccine, exhibiting superior tumor control. The enhanced efficacy of the three-dose regimen is attributable to a homologous prime-boost effect, in which the initial dose primes the immune system and subsequent doses reinforce and prolong the response. In this study, the three-dose schedule also surpassed the four-dose regimen, possibly because of its stronger priming and earlier intervention during initial tumor growth.

(C) Total bioluminescence flux for individual mice corresponding to (B).

(D) Survival curves of mice receiving different vaccination regimens. By the end of the monitoring period, no PBS-treated mice survived (0/5), whereas the 3× group maintained complete survival. Statistics: Log-rank test for trend; $**p < 0.01$.

(E) Mean body weight of mice monitored throughout the study period, showing no significant weight loss, confirming the biosafety of STNvac.

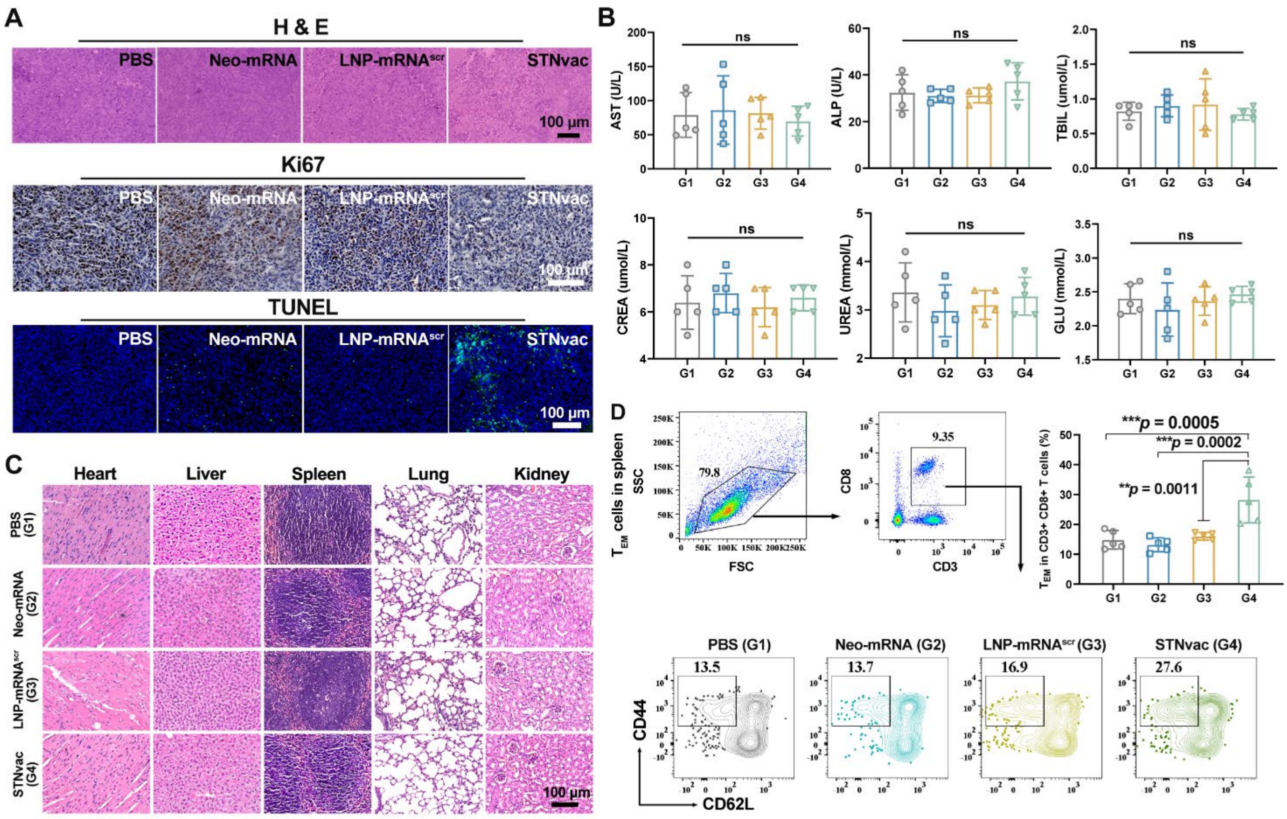

Figure S7. Antitumor effects and acute tolerability of STNvac evaluated in orthotopic HCC mouse model. Related to Figure 3.

(A) H&E, Ki67, and TUNEL staining of dissected tumor sections collected 3 days after the final vaccination (day 10), showing tissue morphology, proliferation, and apoptosis. Scale bars, 100 µm.

(B-C) Acute tolerability assessment of STNvac: (B) Serum biochemical analysis and (C) H&E staining of major organs harvested on day 10. Scale bar, 100 µm.

(D) Flow cytometry analysis of effector memory CD8+ T cells (CD8+ T_{EM}) in spleens collected on day 10. STNvac treatment significantly increased the proportion of CD8+ T_{EM} (28.2% of CD3+ CD8+ T cells) compared with PBS-treated mice (14.8%), indicating enhanced immune protection induced by STNvac.

Statistics: One-way ANOVA for (B) and (D); Data are presented as mean ± SD (n = 5 biological replicates). Significance levels: $^{**}p < 0.01$; $^{***}p < 0.001$.

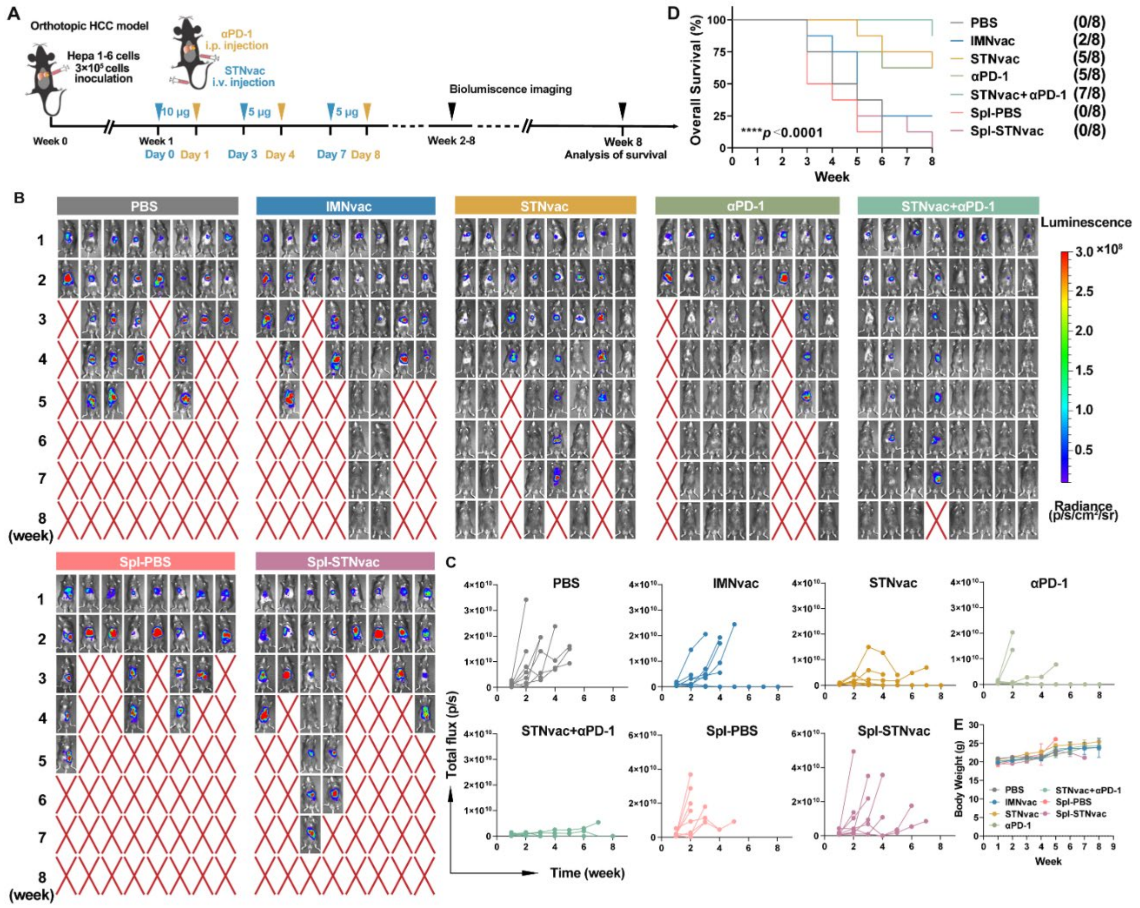

Figure S8. Therapeutic evaluation of STNvac across administration routes, with checkpoint blockade, and in splenectomized mice. Related to Figure 3. (A) Schematic illustration of the vaccination and treatment protocol including seven groups: PBS, intramuscular IMNvac (formulated with the FDA-approved SM-102-based LNP system used in Moderna's mRNA-1273 vaccine), intravenous STNvac, αPD-1 antibody, STNvac + αPD-1 combination, and splenectomized mouse models (Spl-PBS and Spl-STNvac). All mRNA vaccines were administered in a three-dose regimen (days 0, 3, 7; total 20 µg mRNA per mouse; n = 8 mice per group). (B) Bioluminescence images showing tumor burden over the 8-week observation period. Intravenous STNvac induced markedly stronger tumor suppression than intramuscular IMNvac, highlighting the advantage of spleen-targeted delivery. Rapid tumor regression appeared as early as week 2 in the STNvac+αPD-1 group, whereas STNvac monotherapy showed evident responses around weeks 3-4. Responses to αPD-1 monotherapy were heterogeneous, with responding mice showing rapid tumor regression, whereas non-responders exhibited outcomes comparable to PBS-treated controls and experienced early mortality, indicating variable therapeutic sensitivity to PD-1 blockade. In contrast, the STNvac+αPD-1 combination resulted in more consistent tumor control across mice, reflecting an increased response rate compared with αPD-1 monotherapy. In splenectomized cohorts, tumor progression was accelerated and the therapeutic benefit of STNvac observed in intact-spleen mice was largely lost, confirming spleen-dependent efficacy. (C) Total bioluminescence flux for individual mice corresponding to (B). (D) Survival curves of mice in different treatment groups. The STNvac+αPD-1 combination showed the highest overall survival (7/8), followed by STNvac and αPD-1 monotherapy (5/8). In contrast, IMNvac resulted in limited survival benefit (2/8), and all PBS-treated mice succumbed during the observation period. Splenectomy markedly diminished the benefit of STNvac, with Spl-PBS and Spl-STNvac groups succumbing by week 8. (E) Mean body weight of mice monitored throughout the study period, showing no significant loss. Statistics: Log-rank (Mantel-Cox) test for survival analysis (D). Significance levels: ****$p < 0.0001$.

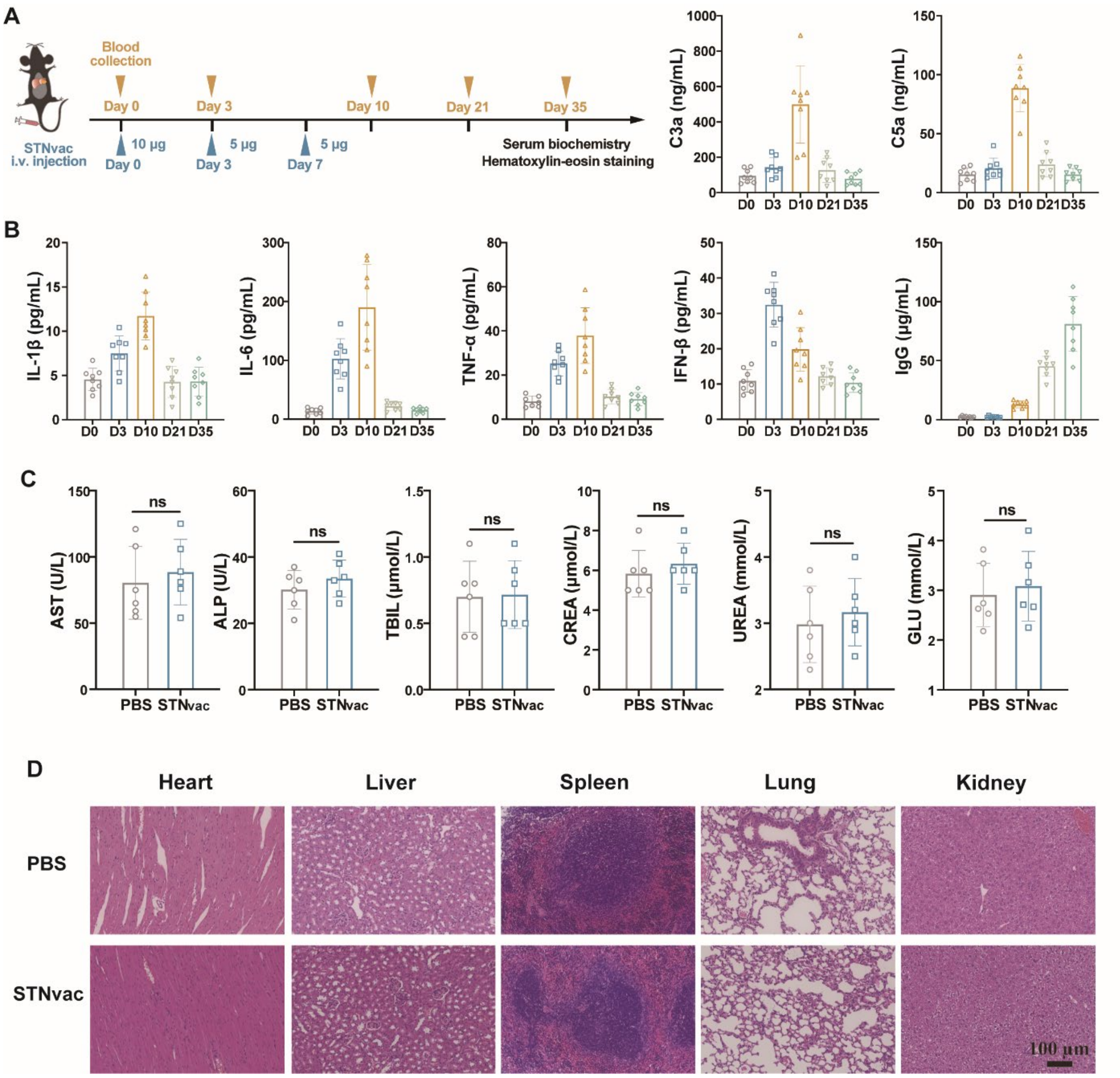

Figure S9. Systemic safety evaluation of STNvac in tumor-free mice. Related to Figure 3.

(A) Schematic illustration of the biosafety assessment protocol. Tumor-free C57BL/6 mice received three intravenous injections of STNvac on days 0, 3, and 7. Blood was collected via orbital capillary sampling at designated time points (days 0, 3, 10, 21 and 35) for cytokine and complement analysis, and at day 35 for serum biochemistry. Major organs were harvested on day 35 for H&E staining.

(B) Serum cytokine (IL-1β, IL-6, TNF-α, IFN-β) and complement (C3a, C5a) levels at different time points, together with total IgG levels. Each bar represents pooled serum obtained from parallel groups of mice (n = 8 per time point). IFN-β peaked on day 3, whereas IL-1β, IL-6, TNF-α, C3a, and C5a peaked on day 10, followed by a progressive decline to baseline by day 21 and stabilization through day 35, indicating transient and self-limited innate activation without sustained systemic inflammation. Total IgG increased beginning on day 10 and continued to rise, reaching a maximum by day 35, consistent with the induction of a sustained humoral response.

(C) Serum biochemistry of liver and kidney function at Day 35. Data represent individual mice (n = 6 biological replicates, mean ± SD). No significant differences were detected between STNvac-treated and control mice.

(D) Representative H&E staining of major organs (heart, liver, spleen, lung, kidney) from PBS control and STNvac-treated mice at day 35, showing no histopathological abnormalities. Scale bar, 100 μm.

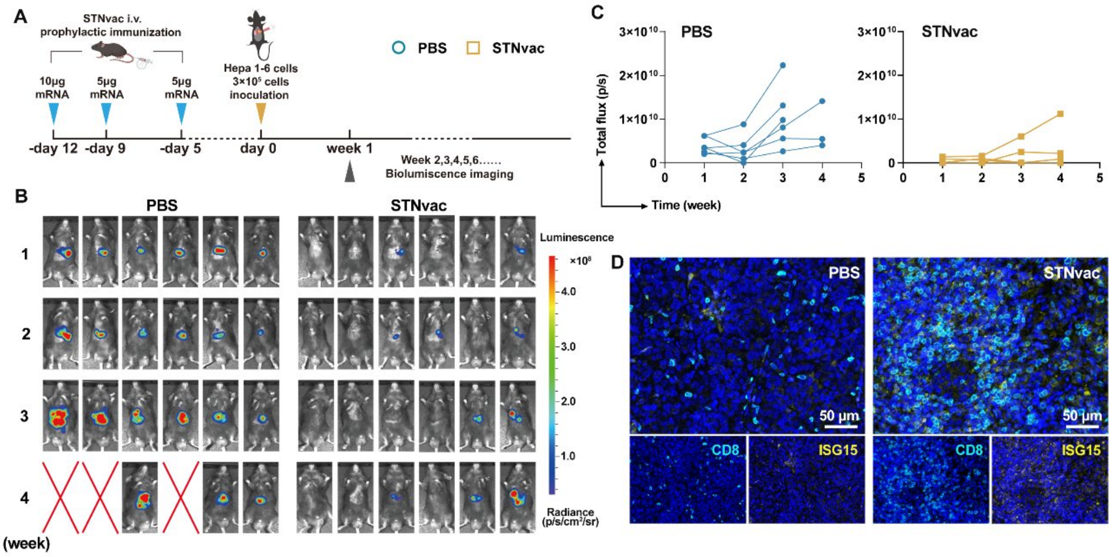

Figure S10. Prophylactic efficacy of STNvac in preventing HCC initiation. Related to Figure 3.

(A) Schematic illustration of the prophylactic vaccination and tumor challenge protocol. Healthy C57BL/6 mice were immunized with STNvac, followed by intrahepatic injection of Hepa1-6-Luc cells 5 days after the final vaccination (n = 6 mice per group).

(B) Bioluminescence imaging showing tumor burden during the 4-week observation period. Rapid tumor progression was observed in the PBS group, whereas STNvac-immunized mice exhibited markedly reduced tumor formation and overall burden, with half showing complete tumor rejection.

(C) Total bioluminescence flux for individual mice corresponding to (B).

(D) Representative multicolor immunofluorescence images of ISG15 and CD8 co-staining in residual lesions from the prophylactic model, confirming the presence of ISG15+ CD8+ T cells in STNvac-immunized mice. Scale bars, 50 μm.

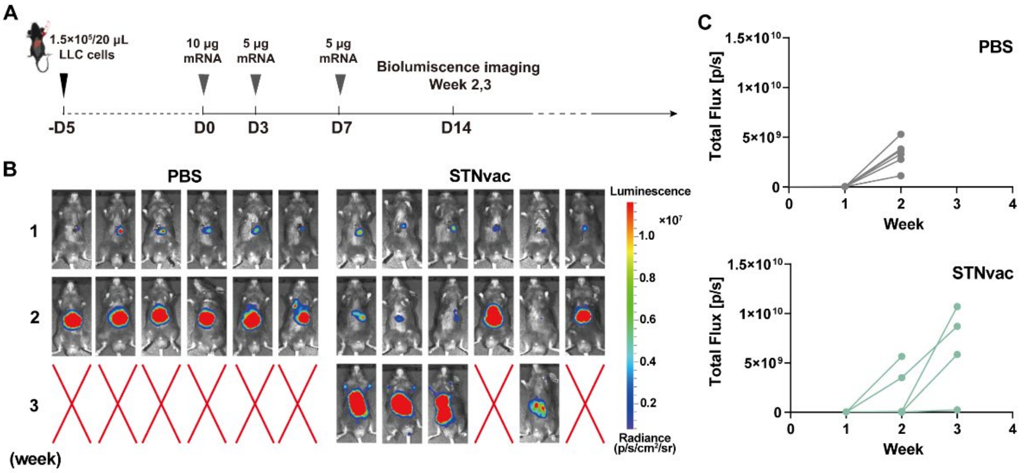

Figure S11. Therapeutic effects of STNvac in the LLC liver metastasis model. Related to Figure 3.

(A) Schematic illustration of the treatment schedule. Lewis lung carcinoma (LLC)-Luc cells were implanted into the livers of C57BL/6 mice to establish a liver metastasis model (n = 6 mice per group).

(B) Bioluminescence imaging showing tumor burden during the 3-week observation period. Rapid tumor progression was observed in the PBS group, whereas STNvac-treated mice showed delayed tumor growth and partially reduced tumor burden, though complete regression was not achieved due to the high malignancy of LLC.

(C) Total bioluminescence flux for individual mice corresponding to (B).

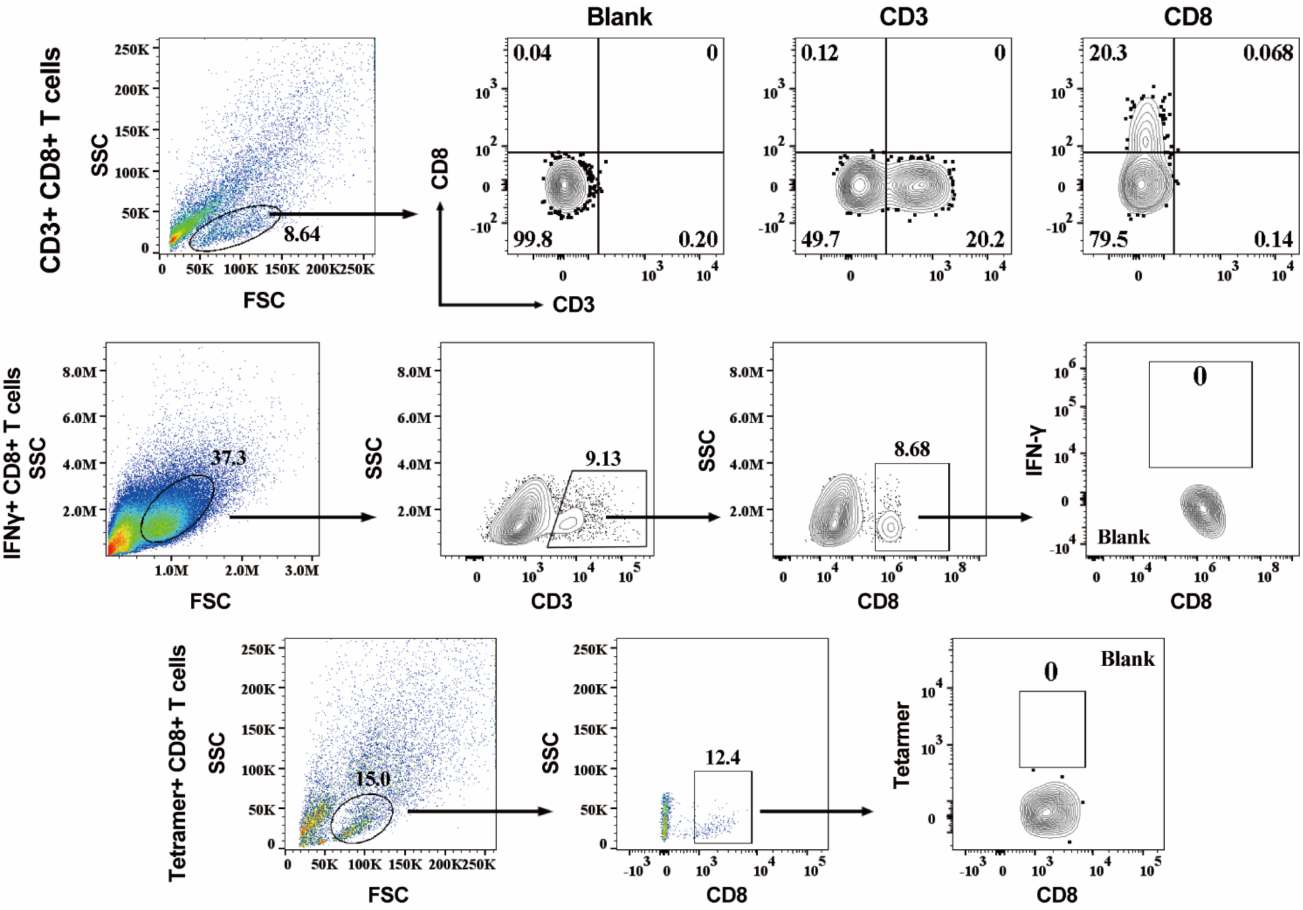

Figure S12. Flow cytometry gating strategy for analyzing T cell states within the tumor microenvironment. Related to Figure 3G.

Representative gating sequence used to identify and quantify intratumoral CD3+ CD8+ T cells, IFN-γ+ CD8+ T cells, and Ptpn2-specific CD8+ T cells in dissected tumor samples after STNvac treatment.

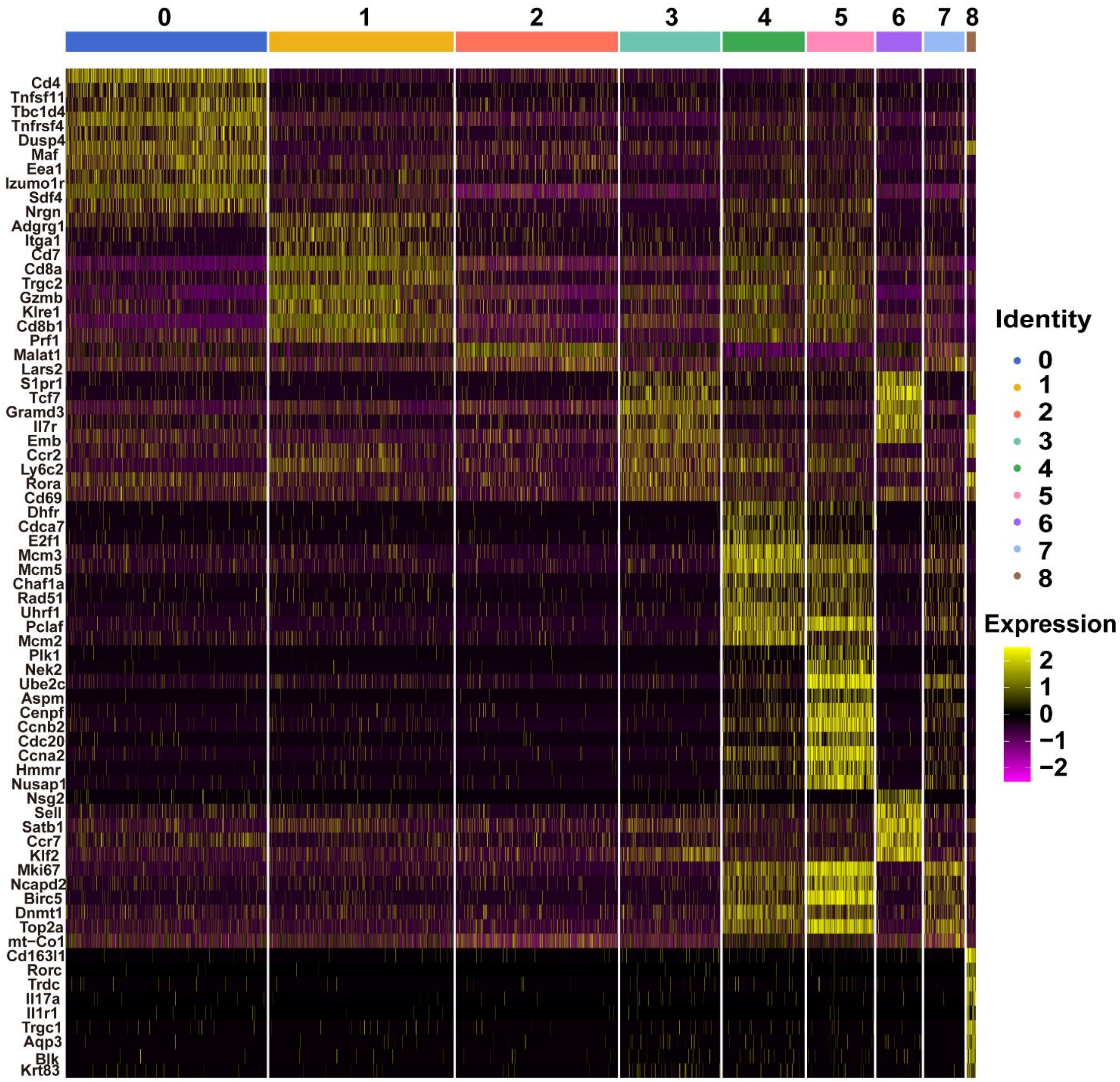

Figure S13. Heatmap showing gene expression profiles across T-cell clusters. Related to Figure 5C.
Representative heatmap illustrating the expression patterns of key marker genes across distinct T-cell clusters identified from single-cell transcriptomic analysis of PBS- and STNvac-treated tumors.

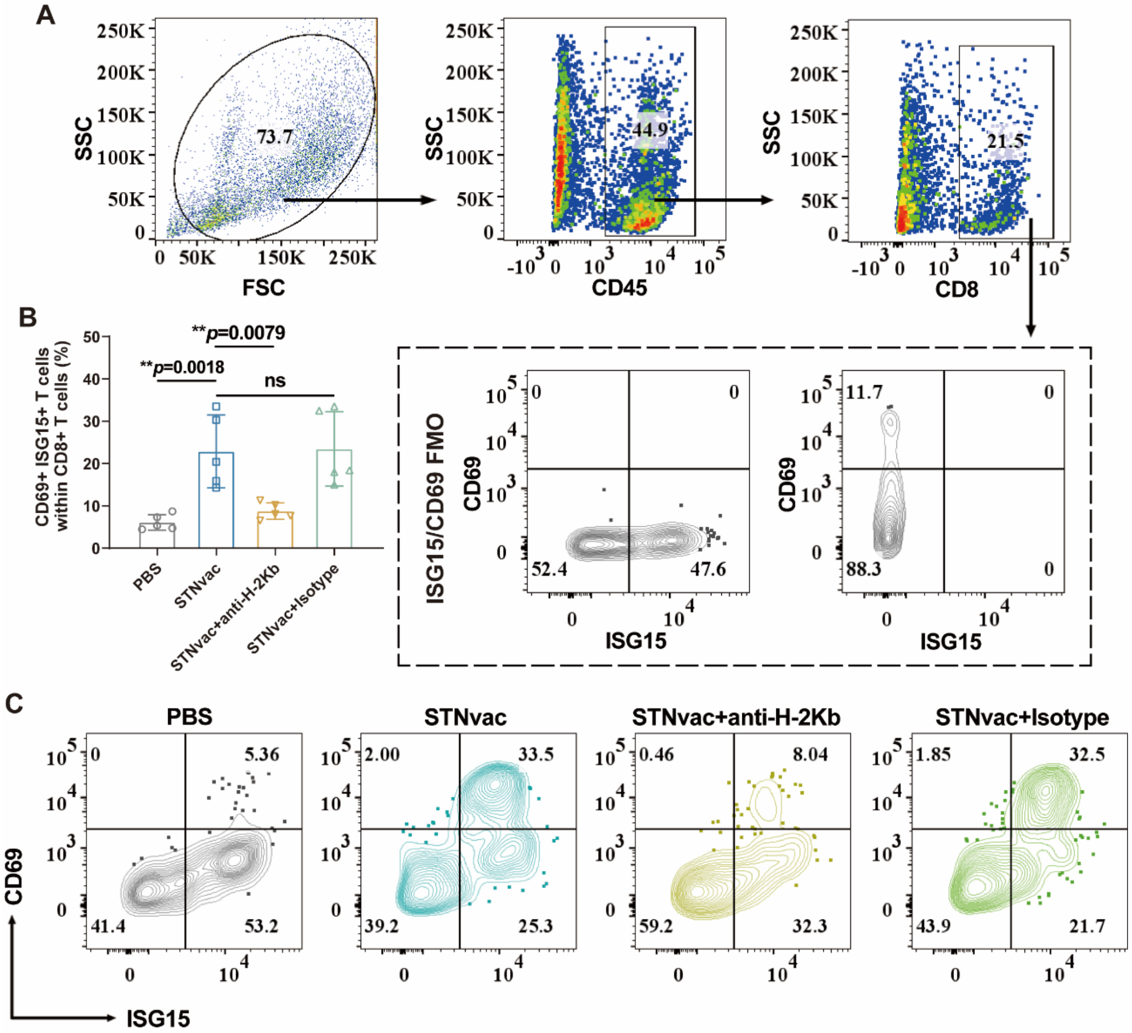

Figure S14. MHC-I blockade attenuates STNvac-induced enrichment of activated ISG15+ CD8+ T cells in tumors. Related to Figure 5.

For MHC-I blockade, anti-H-2Kb antibody or isotype control was administered intraperitoneally 24 h prior to each STNvac vaccination. Tumors were harvested 3 days after the final STNvac dose and processed into single-cell suspensions for flow cytometric analysis. An equal number of CD45+ events were acquired per sample.

(A) Gating strategy for the analysis of tumor-infiltrating lymphocytes. Fluorescence-minus-one (FMO) controls for ISG15 (secondary antibody included, primary antibody omitted) and CD69 were used to define positivity thresholds.

(B) Quantification of the frequency of ISG15+ CD69+ cells within tumor-infiltrating CD8+ lymphocytes across treatment groups, including PBS, STNvac, STNvac plus anti-H-2Kb antibody, and STNvac plus isotype control.

(C) Representative density plots of ISG15 and CD69 expressions within the CD45+ CD8+ population in each treatment group.

Statistics: One-way ANOVA. Data are presented as mean ± SD (n = 5 biological replicates). Significance levels: ** $p < 0.01$.

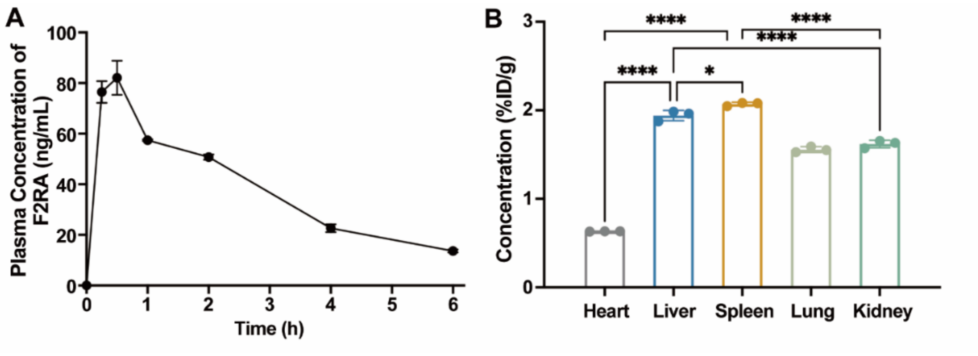

Figure S15. Pharmacokinetics and biodistribution of F2RA (SCH79797). Related to Figure 6.

(A) Plasma concentration-time profile of F2RA following intraperitoneal injection, measured by LC-MS/MS coupled with HPLC (Shimadzu UPLC 30AD with AB Sciex API5500 triple-quadrupole mass spectrometer). Plasma samples were collected at 0.25-6 h post-administration and analyzed by multiple-reaction monitoring (Q1/Q3 = 372.1/356.3 m/z) using a Shim-pack XR-ODS III C18 column (2.0 × 50 mm, 1.6 μm). Plasma levels peaked at 0.5 h, indicating rapid systemic absorption.

(B) Biodistribution of F2RA in major organs (heart, liver, spleen, lung, kidney) at 0.5 h post-injection, showing highest enrichment in the liver and spleen.

Statistics: One-way ANOVA for (B); Data are presented as mean ± SD (n = 3 biological replicates). Significance levels: *$p < 0.05$; ****$p < 0.0001$.

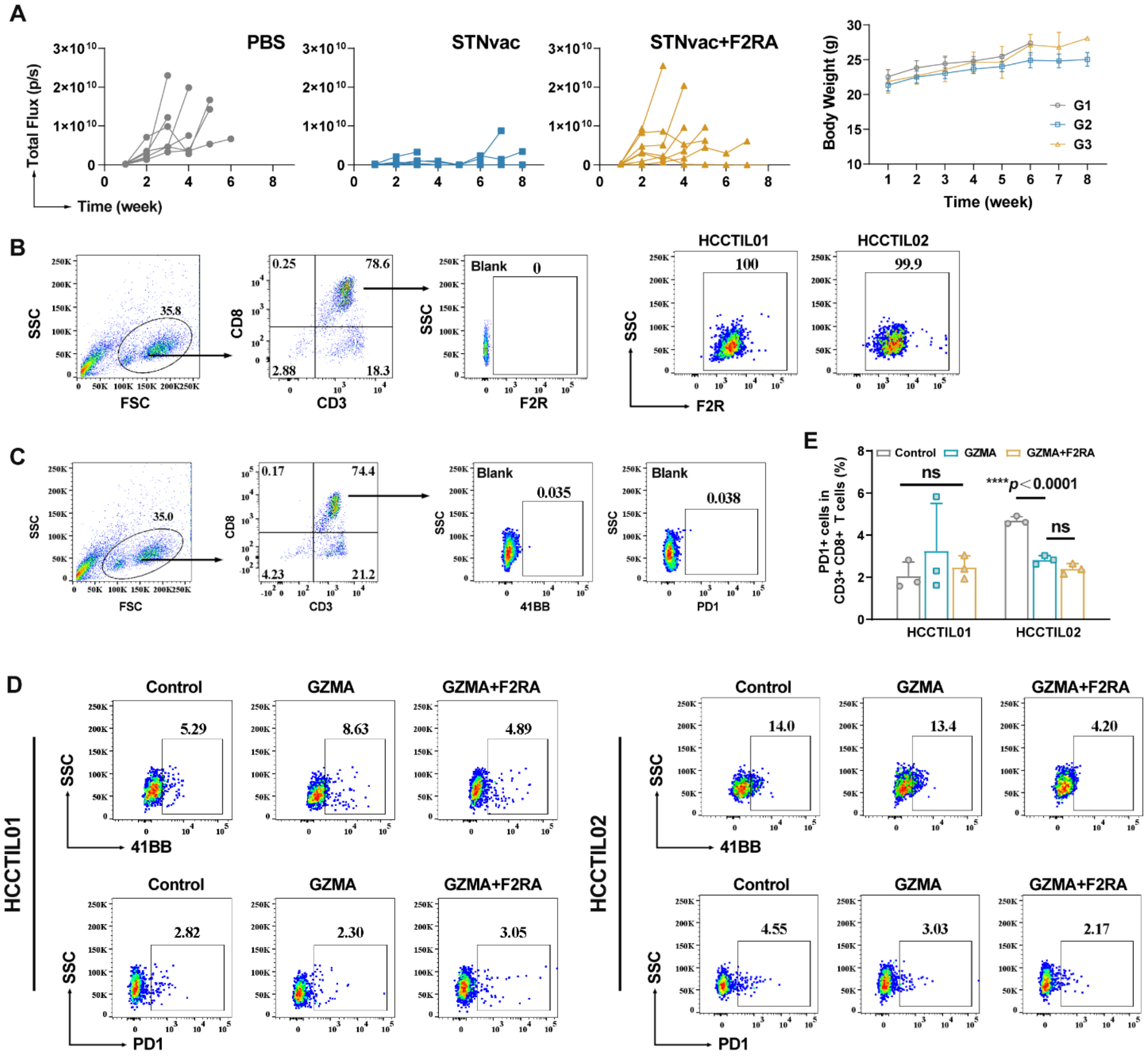

Figure S16. Effect of GZMA-F2R interaction on STNvac efficacy and human CD8+ TIL activation. Related to Figure 6.

(A) Individual tumor growth curves assessed by total fluorescence flux and mean body weight of the mice (related to Figure 6C).

(B) Flow cytometry analysis of F2R expression on CD3+ CD8+ TILs.

(C-E) Flow cytometry analysis of 41BB and PD1 expressions of the CD3+ CD8+ TILs after indicated administration (related to Figure 6H). (C) Gating strategies. (D) Representative scatter plots. (E) Quantitative analysis of 41BB+ and PD1+ CD3+ CD8+ T cell frequencies (n = 2 biological replicates, each analyzed in triplicate). Statistics: One-way ANOVA. Data are presented as mean ± SD. Significance levels: ****$p < 0.0001$.

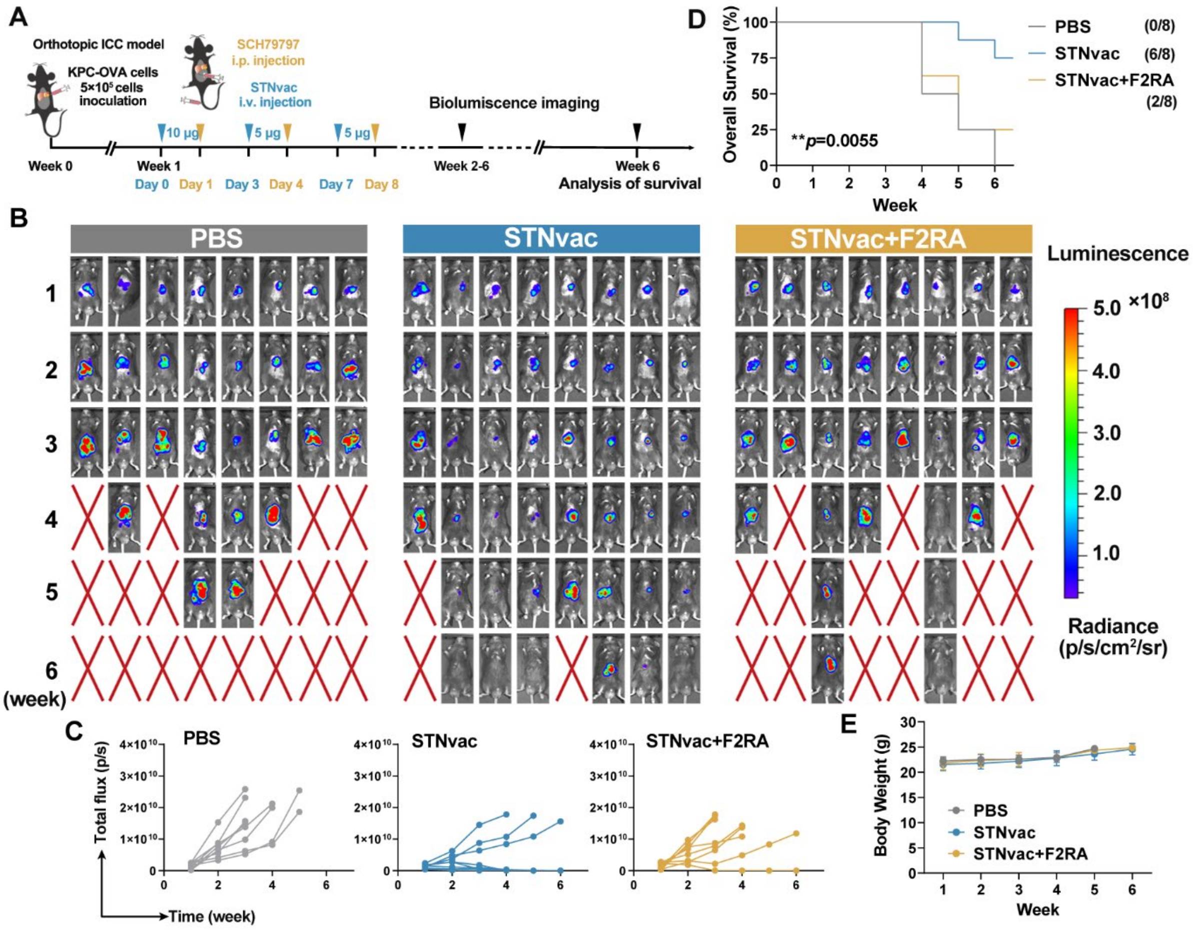

Figure S17. Validation of the GZMA-F2R pathway in an orthotopic intrahepatic cholangiocarcinoma (ICC) model derived from the KPC-OVA cell line. Related to Figure 6.

(A) Schematic illustration of the vaccination and treatment protocol. An orthotopic ICC model was established by intrahepatic implantation of KPC-OVA-Luc cells (KrasG12D; Trp53R172H; Pdx1-Cre) engineered to express the model antigen ovalbumin (OVA) and firefly luciferase (n = 8 mice per group). Mice received PBS, OVA-mRNA-loaded STNvac, or STNvac co-administered with the F2R antagonist (F2RA, SCH79797).

(B) Bioluminescence imaging showing tumor burden during the 6-week observation period. Co-administration of F2RA impaired the antitumor efficacy of OVA-STNvac, consistent with findings in the Hepa1-6 HCC model.

(C) Total bioluminescence flux for individual mice corresponding to (B).

(D) Survival curves of mice in different treatment groups, demonstrating reduced therapeutic benefit when the GZMA-F2R axis is blocked.

(E) Mean body weight of mice monitored throughout the study period, showing no significant systemic toxicity.

Statistics: Log-rank (Mantel-Cox) test for survival analysis (D). Significance levels: $**p < 0.01$.

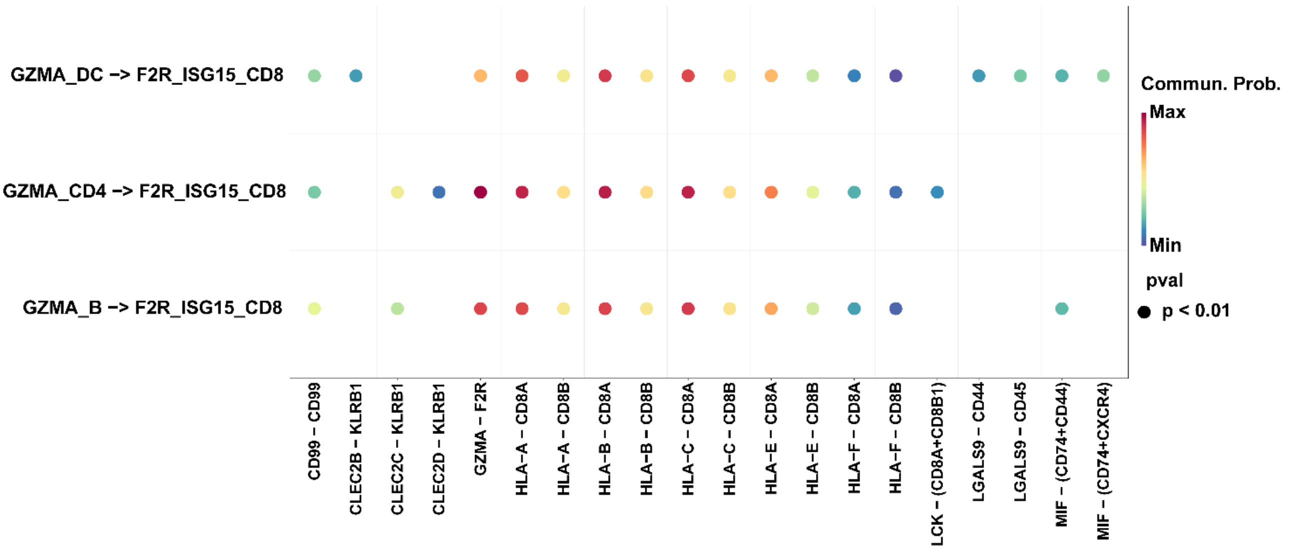

Figure S18. Predicted cell-cell interaction between F2R+ ISG15+ CD8+ T cells and GZMA+ APCs (DCs, B cells, CD4+ T cells) derived from scRNA-seq data of treatment-naive HCC patients (GEO: GSE156625). Related to Figure 6.

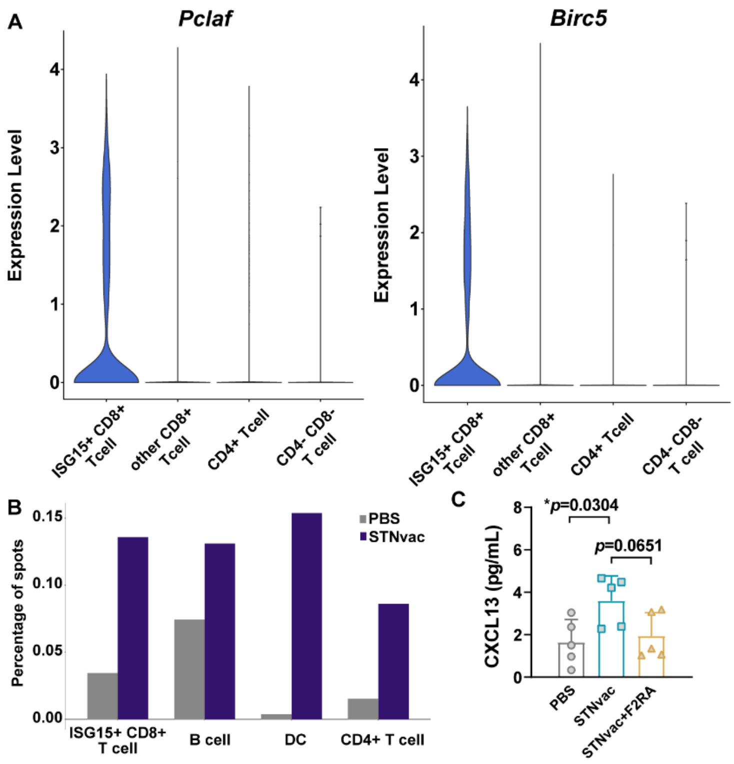

Figure S19. Spatial annotation of ISG15+ CD8+ T cells and CXCL13 expression. Related to Figures 6 and 7.

(A) Expression of ISG15+ CD8+ T cell-associated markers across T cell subsets in single-cell transcriptomic data. *Pclaf* and *Birc5* were selected as alternative signatures to represent ISG15+ CD8+ T cells in Figure 7C, as ISG15 was not included in the spatial probe list.

(B) Percentage of positive spatial transcriptomic spots annotated as ISG15+ CD8+ T cells, B cells, DCs, or CD4+ T cells in tumor tissues, corresponding to the regions shown in Figure 7C.

(C) ELISA analysis of CXCL13 in dissected tumors from PBS, STNvac and STNvac+F2RA groups, 3 days after the final treatment (related to Figure 6C). One-way ANOVA. Data are presented as mean ± SD (n = 5 biological replicates). Significance levels: *$p < 0.05$.

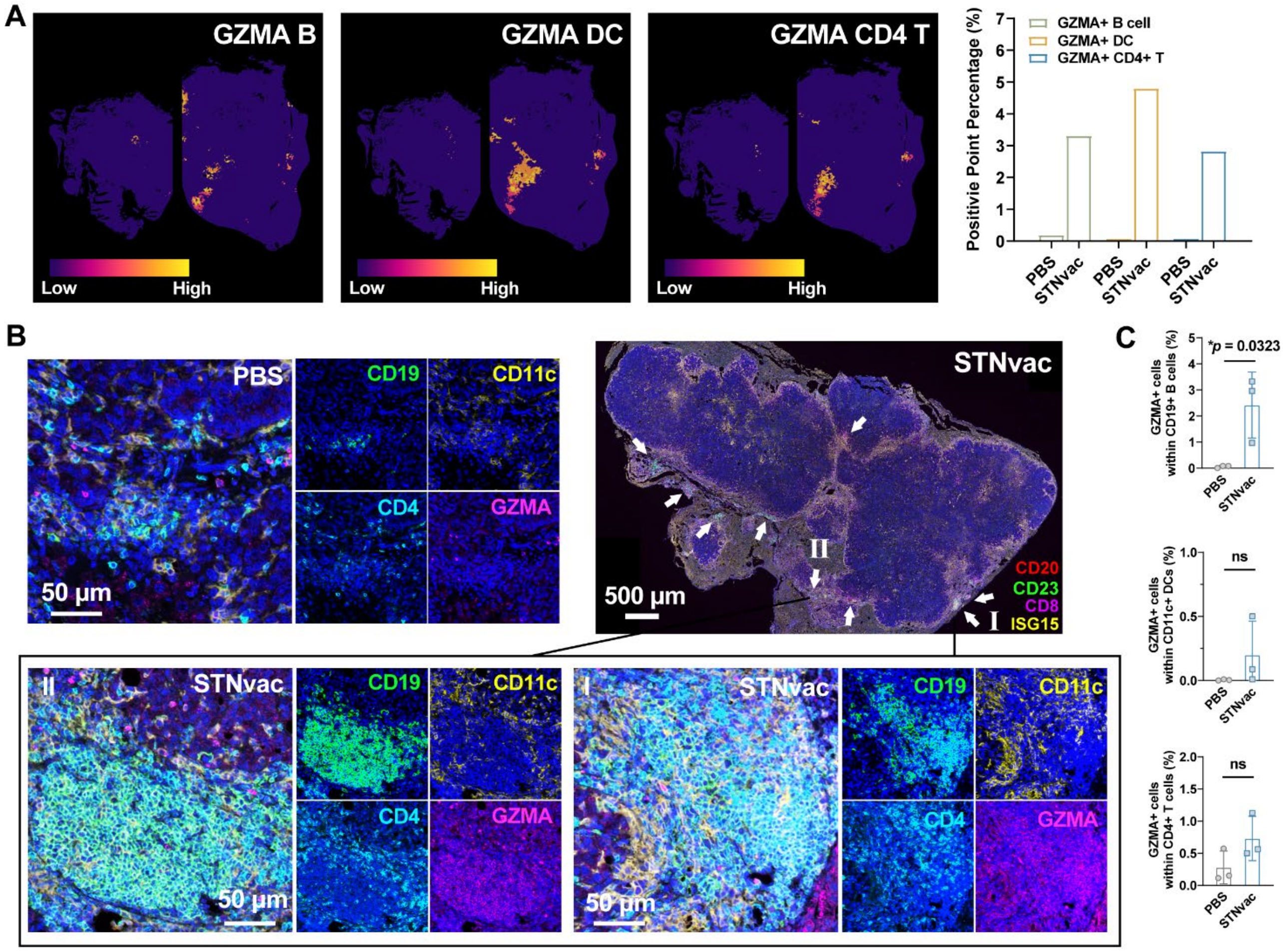

Figure S20. Spatial and histological validation of GZMA expression in APC subsets following STNvac treatment. Related to Figure 7.

(A) Spatial transcriptomic analysis of the same tumor sections shown in Figure 7A, quantifying the proportion of GZMA+ cells within B cells, DCs, and CD4+ T-cell populations.

(B) Multicolor immunofluorescence staining for CD19, CD11c, CD4, and GZMA in tumors from PBS- and STNvac-treated mice. The whole-section image of the STNvac group corresponds to that shown in Figure 7E, serving as a spatial reference to align consecutive sections. Regions I and II, indicated by white arrows, are displayed at higher magnification to illustrate the enrichment of GZMA+ APCs within and surrounding TLS regions. The PBS panel shows a magnified area corresponding to the arrow-indicated region in Figure 7E. Scale bars, 50 μm.

(C) Quantitative analysis of the percentage of GZMA+ cells within each APC subset (n = 3 biological replicates). Data are presented as mean ± SD. Statistical significance was determined by unpaired two-tailed Student's t-test (*$p < 0.05$).

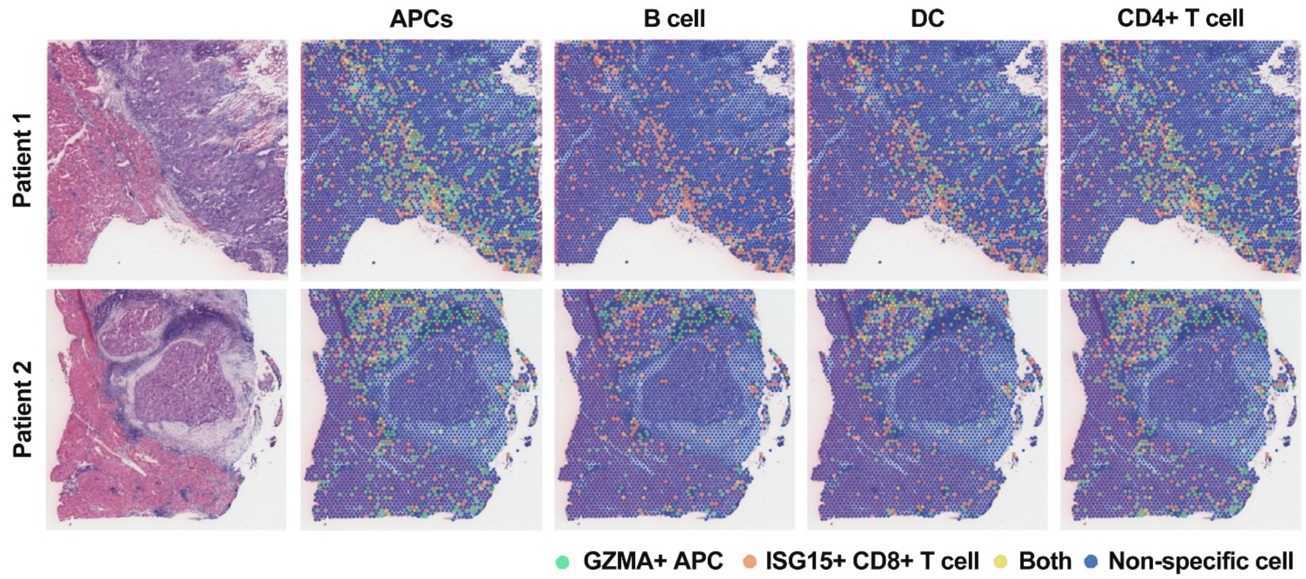

Figure S21. Spatial colocalization of ISG15+ CD8+ T cells and GZMA+ APCs in human HCC tissues. Related to Figure 7.

Spatial transcriptomic analysis of nine HCC samples from Mendeley Data: http://www.doi.org/10.17632/skrx2fz79n.1 (Liu's study; J. Hepatol., 2023, 78, 770, Ref 55). Two immune-inflamed tumors with pronounced immune-cell infiltration were selected for detailed evaluation. GZMA+ APCs (including GZMA+ DCs, B cells, and CD4+ T cells) were analyzed for their spatial relationship with ISG15+ CD8+ T cells, which were consistently localized around GZMA+ APCs at the tumor periphery, indicating conserved spatial organization between human and murine HCC.

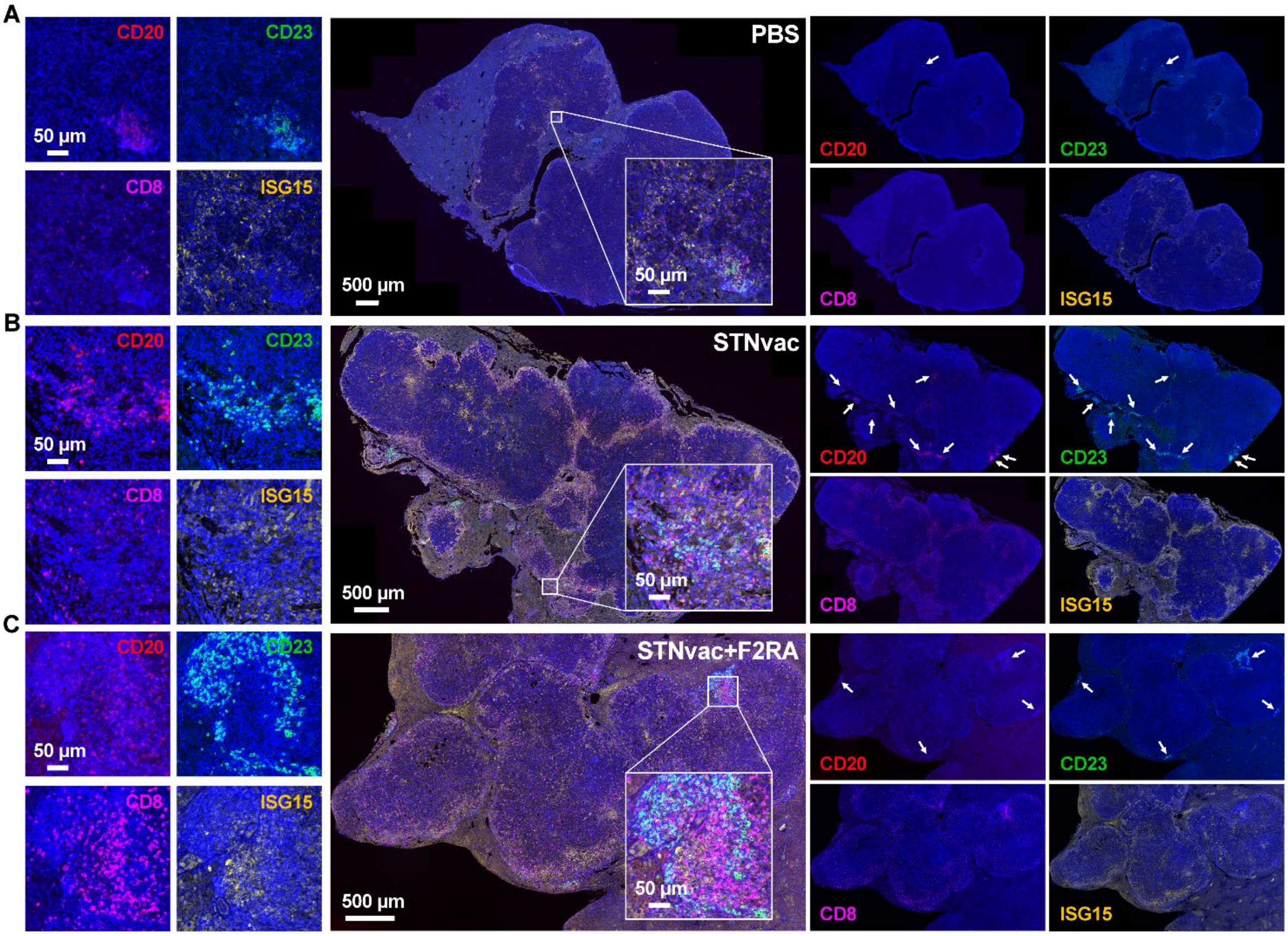

Figure S22. Representative multicolor and single-channel immunofluorescence images of TLSs and ISG15+ CD8+ T cells in tumors from different treatment groups. Related to Figure 7.

(A-C) Tumor sections from (A) PBS, (B) STNvac, and (C) STNvac+F2RA groups stained for CD20, CD23, CD8, and ISG15. Shown are representative composite and single-channel views, as well as higher-magnification images of TLS regions corresponding to those in Figure 7E.

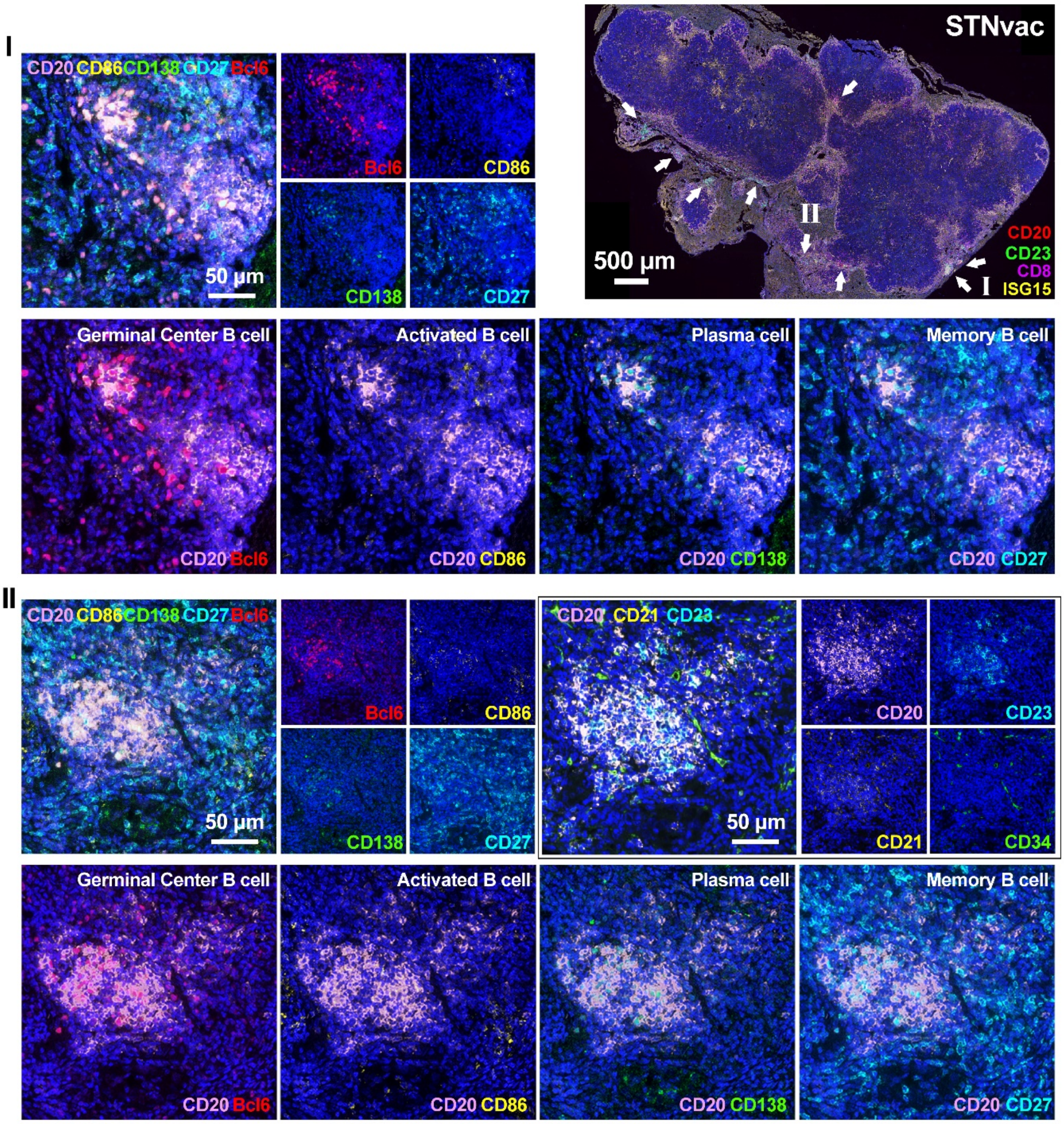

Figure S23. Detailed characterization of B-cell maturation and TLS organization in STNvac-treated tumors. Related to Figure 7.

Tumor sections from the STNvac group (consecutive to the sample shown in Figure 7E) were analyzed by multicolor immunofluorescence. The whole-section image corresponds to Figure 7E and serves as a spatial reference for aligning consecutive sections. Regions I and II are shown at higher magnification. Scale bars, 50 µm.

Region I: Five-marker staining (CD20, Bcl6, CD86, CD138, and CD27) revealed distinct B-cell subsets within TLSs. Representative merged and single-channel images are presented, together with enlarged dual-marker views highlighting CD20+ Bcl6+ germinal-center B cells, CD20+ CD86+ activated B cells, CD20- CD138+ plasma cells, and CD20+ CD27+ memory-like B cells.

Region II: The same five-color panel as Region I, together with an additional four-marker panel (CD20, CD21, CD23, and CD34), illustrates CD21+ FDC networks and CD34+ HEV-like structures at TLS boundaries.

Similar B-cell subsets, as well as CD21+ and CD34+ structures were consistently observed in additional biological replicates (data not shown), confirming the reproducibility of TLS organization.

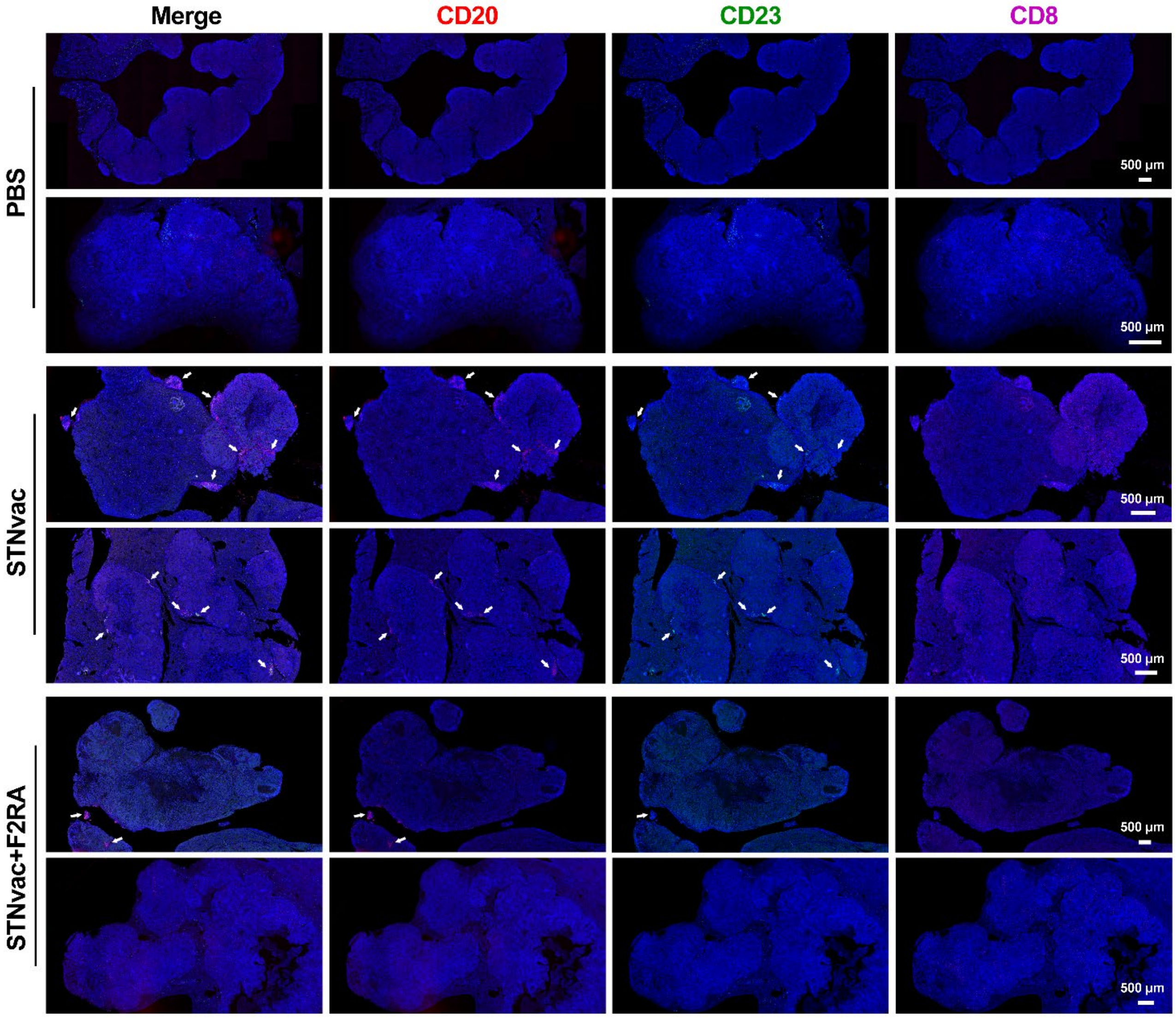

Figure S24. Multicolor immunofluorescence validation and quantitative assessment of TLSs across biological replicates. Related to Figure 7G.

Representative CD20/CD23 dual-staining images from PBS-, STNvac-, and STNvac+F2RA-treated tumors. Shown are the two additional biological replicates not depicted in Figure 7E and Figure S21; together, these three tumors were used for TLS quantification in Figure 7G. For quantification, TLSs were defined as organized CD20+ CD23+ lymphoid aggregates consistent with secondary follicular TLSs described in the literature (Science, 2022, 2528, eabf9419; Cancer Res, 2018, 78, 1308; Immunity, 2022, 55, 527), and only aggregates with a longest axis greater than 100 μm were counted to exclude small, non-structured B-cell clusters. This unified definition was used for TLS quantification in Figure 7G. In the STNvac+F2RA group, CD20+ aggregates lacking CD23 signal were observed and were therefore not classified as mature TLSs in this study. Scale bars, 500 μm.

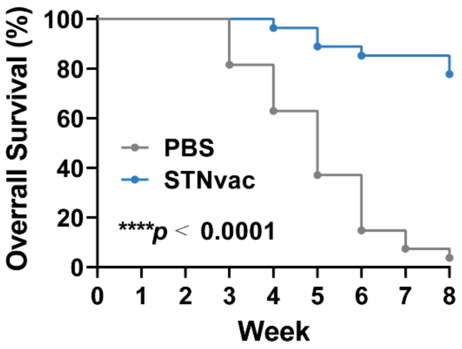

Figure S25. Pooled survival analysis of STNvac across independent orthotopic HCC experiments. Related to Figures 3 and 6.

Kaplan-Meier survival data were combined from four independent orthotopic HCC studies (Figures 3D, 6D, S6D, and S8D), each including PBS and STNvac treatment groups (total n = 27 mice per group). STNvac significantly improved overall survival compared with PBS (77.8% vs 3.7%; log-rank test, ****$p < 0.0001$), confirming the reproducibility and robust efficacy of the vaccine across independent cohorts.

Table S1. The mRNA sequence of Hepa1-6/LLC neoantigens for neoantigen vaccine preparation. Related to STAR Methods.

**Hepa 1-6 neoantigen mRNA sequence**
ATGGCCGTCATGGCGCCCCGAACCCTCCTCCTGCTACTCTCGGGGGCCCTGGCCCTGACCCAGACCTGGG<br>CGGGCTCCATGAAGGCCCGGAACTACCTGCAGTTCCTGCCCAGCAAGACCAAGGTGGCCCGGGGCCGG<br>AAGCGGCGGAGCCGGGGCGAGCACTACCGGTACAAGGTGAGCCTGCCCGGCGGCCAGCACGCCCGGGG<br>CCGGAAGCGGCGGAGCCACGTGCTGTGGGACCTGAAGCAGATGTTCCGGTGCGCCGTGCTGAAGAACC<br>GGGGCCGGAAGCGGCGGAGCTGGGACACCTGCACCACCTACAAGTGGCAGAAGACCCTGGAGGGCCAC<br>GACCGGGGCCGGAAGCGGCGGAGCCTGAGCACCTACCGGACCGCCTGCACCCTGCGGTTCGTGCAGAA<br>GCGGTGCCGGGGCCGGAAGCGGCGGAGCCTGTACACCCACTTCCTGCAGCTGCCCCTGGCCGCCACCGG<br>CTTCAGCGTGCGGGGCCGGAAGCGGCGGAGCAAGCGGTGGCTGTACTGGCAGCCCACCCTGACCAAGA<br>TGGGCTTCGTGAGCCGGGGCCGGAAGCGGCGGAGCGGATCCATCATTGCTGGCCTGGTTCTCCTTGGAG<br>CTGTGATCACTGGAGCTGTGGTCGCTGCCGTGATGTGGAGGAGGAAGAGCTCAGATAGAAAAGGAGGG<br>AGTTACACTCAGGCTGCAAGCAGTGACAGTGCCCAGGGCTCTGATGTGTCCCTCACAGCTTGTAAAGTG<br>TGATAA
**LLC neoantigen mRNA sequence**
ATGGCCGTCATGGCGCCCCGAACCCTCCTCCTGCTACTCTCGGGGGCCCTGGCCCTGACCCAGACCTGGG<br>CGGGCTCCCCCGGCGCCGCCCCCGTGGTGGTGGCCCACGCCCGGCTGAGCCGGGTGCTGCGGGGCCGGA<br>AGCGGCGGAGCCCCTACAAGTGCACCAAGTGCGGCCGGGCCTTCACCCGGAGCAGCACCCTGCGGGGC<br>CGGAAGCGGCGGAGCAACCTGACCCTGCACTACCGGACCCTGCTGGTGGACCGGCCCTACGACTGCCGG<br>GGCCGGAAGCGGCGGAGCGACCCCTGGGCCGGCTACCGGTACAGCGGCAAGCTGCGGCCCCACTACCC<br>CCGGGGCCGGAAGCGGCGGAGCGAGGAGGACACCGACACCAAGCAGATCTACTTCTACCTGTTCAAGC<br>TGCTGCGGGGCCGGAAGCGGCGGAGCCTGTTCGGCCTGATCAACGTGACCCCCAACATGCTGCCCCTGG<br>TGAAGAAGCGGGGCCGGAAGCGGCGGAGCGCCGGCAACCGGCACATCAAGTTCTGCTACCTGGACGAC<br>AGCAAGACCAGCCGGGGCCGGAAGCGGCGGAGCATCATTGCTGGCCTGGTTCTCCTTGGAGCTGTGATC<br>ACTGGAGCTGTGGTCGCTGCCGTGATGTGGAGGAGGAAGAGCTCAGATAGAAAAGGAGGGAGTTACAC<br>TCAGGCTGCAAGCAGTGACAGTGCCCAGGGCTCTGATGTGTCCCTCACAGCTTGTAAAGTGTGATAA

Note: The blue font represents the signal peptide and MITD structure, the red font represents the neoantigen sequence, and the green font represents the Furin sequence.
